# Polyphenol Utilization Proteins in the Human Gut Microbiome

**DOI:** 10.1128/aem.01851-21

**Published:** 2022-02-08

**Authors:** Bo Zheng, Yinchao He, Pengxiang Zhang, Yi-Xin Huo, Yanbin Yin

**Affiliations:** a Key Laboratory of Molecular Medicine and Biotherapy, School of Life Sciences, Beijing Institute of Technology, Beijing, China; b Nebraska Food for Health Center, Department of Food Science and Technology, University of Nebraska—Lincoln, Lincoln, Nebraska, USA; c Department of Computer Science and Engineering, University of Nebraska—Lincoln, Lincoln, Nebraska, USA; d Department of Computer Science and Engineering, University of California, San Diego, California, USA; University of Naples Federico II

**Keywords:** polyphenol, polyphenol utilization proteins, PUP, PUP gene clusters, microbiome, gut microbiota

## Abstract

Dietary polyphenols can significantly benefit human health, but their bioavailability is metabolically controlled by human gut microbiota. To facilitate the study of polyphenol metabolism for human gut health, we have manually curated experimentally characterized polyphenol utilization proteins (PUPs) from published literature. This resulted in 60 experimentally characterized PUPs (named seeds) with various metadata, such as species and substrate. Further database search found 107,851 homologs of the seeds from UniProt and UHGP (unified human gastrointestinal protein) databases. All PUP seeds and homologs were classified into protein classes, families, and subfamilies based on Enzyme Commission (EC) numbers, Pfam (protein family) domains, and sequence similarity networks. By locating PUP homologs in the genomes of UHGP, we have identified 1,074 physically linked PUP gene clusters (PGCs), which are potentially involved in polyphenol metabolism in the human gut. The gut microbiome of Africans was consistently ranked the top in terms of the abundance and prevalence of PUP homologs and PGCs among all geographical continents. This reflects the fact that dietary polyphenols are consumed by the African population more commonly than by other populations, such as Europeans and North Americans. A case study of the Hadza hunter-gatherer microbiome verified the feasibility of using dbPUP to profile metagenomic data for biologically meaningful discovery, suggesting an association between diet and PUP abundance. A Pfam domain enrichment analysis of PGCs identified a number of putatively novel PUP families. Lastly, a user-friendly web interface (https://bcb.unl.edu/dbpup/) provides all the data online to facilitate the research of polyphenol metabolism for improved human health.

**IMPORTANCE** Long-term consumption of polyphenol-rich foods has been shown to lower the risk of various human diseases, such as cardiovascular diseases, cancers, and metabolic diseases. Raw polyphenols are often enzymatically processed by gut microbiome, which contains various polyphenol utilization proteins (PUPs) to produce metabolites with much higher bioaccessibility to gastrointestinal cells. This study delivered dbPUP as an online database for experimentally characterized PUPs and their homologs in human gut microbiome. This work also performed a systematic classification of PUPs into enzyme classes, families, and subfamilies. The signature Pfam domains were identified for PUP families, enabling conserved domain-based PUP annotation. This standardized sequence similarity-based PUP classification system offered a guideline for the future inclusion of new experimentally characterized PUPs and the creation of new PUP families. An in-depth data analysis was further conducted on PUP homologs and physically linked PUP gene clusters (PGCs) in gut microbiomes of different human populations.

## INTRODUCTION

Polyphenols are one of the largest groups of secondary metabolites in plants with highly variable structure and occurrence. These phytonutrients are naturally found in a wide range of fruits, vegetables, and cereals, as well as plant-based food products (1–3). Polyphenols are often glycosylated and closely interact with carbohydrates in plant cell walls. Due to the ubiquity of polyphenols in the daily diet of humans and animals, numerous epidemiologic studies have found that the long-term consumption of polyphenol-rich foods can lower the risk of various human diseases, such as cardiovascular diseases, cancers, and metabolic syndromes (4–6). Therefore, the research of polyphenols, their synthesis in plants, and their degradation in microbes has gained tremendous attention in the past few years (7).

Raw polyphenols from plants have low bioavailability in the human digestive system. However, the gut microbiota can use various polyphenol utilization proteins (PUPs) to enzymatically process the raw polyphenols to produce smaller polyphenol metabolites with higher bioaccessibility to human gastrointestinal cells. These processed metabolites are much easier to absorb through the human digestive system (8, 9) and benefit human health. In the past, microbially produced PUPs have been experimentally characterized from various environments, including human gut, hot spring, and soils (10–15). However, these experimentally characterized PUPs are dispersed in the literature, which hinders systematic and comparative studies of polyphenol catabolism in various ecosystems using the large amounts of ever-increasing microbial genome and metagenome data in the databases.

The highly complex structures of polyphenols require various microbial PUP enzymes for different catabolic processes, such as hydrolysis, cleavage, and reduction reactions. A comprehensive online repository, which collates and classifies PUPs with known substrates and products from literature, will certainly enhance experimental characterization of new PUPs. To this end, we have developed dbPUP (https://bcb.unl.edu/dbpup/) for experimentally verified PUPs from literature and their homologs in the UniProt database and the database of unified human gastrointestinal protein (UHGP) catalog (16). The only available database for polyphenol research is Phenol-Explorer (http://phenol-explorer.eu/) (17). However, it provides statistics of polyphenol content only in foods, while our dbPUP focuses on providing a comprehensive classification of experimentally verified PUPs and their sequence homologs.

## RESULTS AND DISCUSSION

### Data curation found 60 experimentally characterized PUPs (seeds).

**FIG 1:**
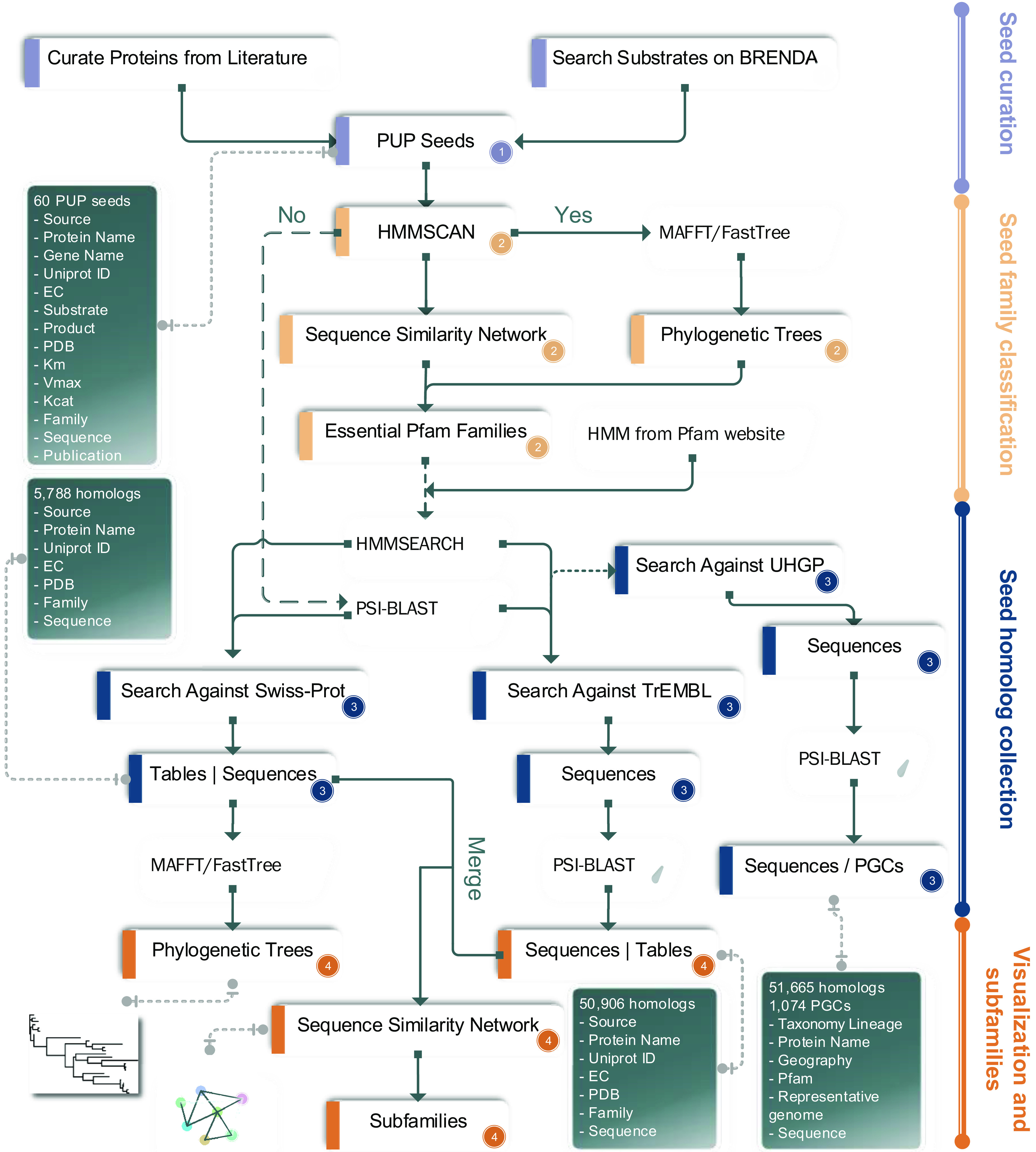
Workflow of the development of dbPUP. The four major tasks are shown as vertical bars (right): (1) seed protein curation, (2) seed sequence analysis for class and family level classification, (3) data expansion to include seed homologs from UniProt and UHGP, and (4) homolog data visualization and classification. The detailed data, methods, and tools are provided in the workflow.

**FIG 2:**
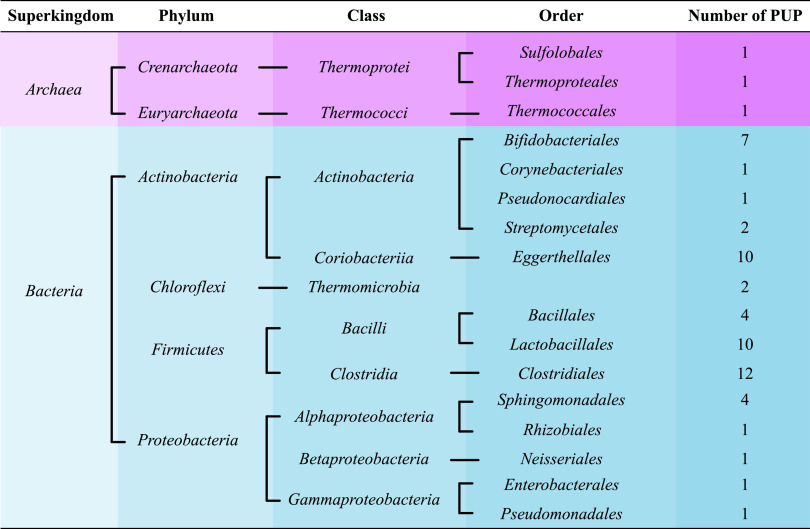
Taxonomic distribution of the 60 seed PUPs.

Our PUP collection pipeline (Fig. 1) starts with a literature curation. We obtained in total 60 PUPs from 91 published papers and the BRENDA database. These seed proteins were assigned to 16 different taxonomic orders of 6 phyla (Fig. 2). A majority of the 60 PUPs (26 proteins, 43%) belong to *Firmicutes*, followed by *Actinobacteria* (21 sequences, 35%). All these 60 seed proteins were kept after manual curation according to two criteria: (i) protein was from prokaryote (*Bacteria* or *Archaea*) and (ii) substrates and products were experimentally verified by enzymatic assay.

### The 60 PUP seeds were classified into 26 protein families of 6 enzyme classes.

**FIG 3:**
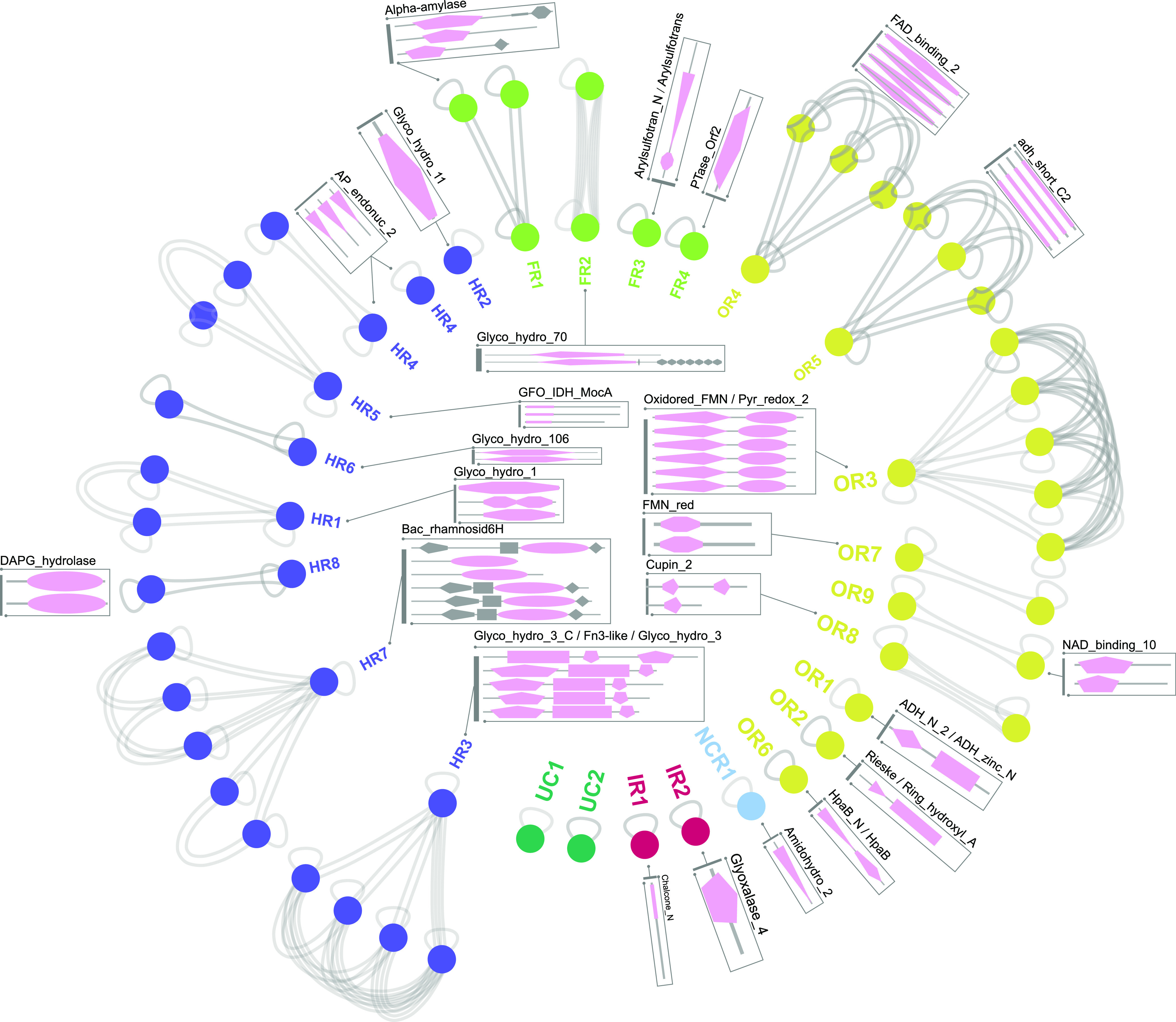
Sequence similarity networks of 60 PUP seed proteins. Gray lines mean that two connected proteins (nodes) are similar to each other with an E value of <10^−5^. Domain architecture for each PUP is shown in a box beside each family. Protein nodes are colored according to their enzyme class assignment (see Results and Discussion). Domains shown in pink are signature Pfam domains.

Figure 3 and Fig. S1 showed that the 60 PUP seeds (except for I5AX49 and Q65JC2) contain in total 40 unique Pfam protein domains. According to a set of criteria (see Materials and Methods) and the sequence similarity network (SSN) analysis (Fig. 3), the 58 PUPs with Pfam domains were classified into 24 families, each with a signature Pfam domain or multidomain combination (Table 1). The two PUPs (I5AX49 and Q65JC2) without Pfam domains were defined as two unclassified families. Therefore, dbPUP contains in total 26 protein families of six enzymatic classes according to their enzyme commission (EC) numbers at the first level (i.e., the chemical reaction they catalyze). (i) Oxidation/reduction reactions (OR): this class contains 9 families. (ii) Functional group transfer reactions (FR): this class contains 4 families. (iii) Hydrolysis reactions (HR): this class contains 8 families. (iv) Nonhydrolytic cleaving reactions (NCR): this class contains 1 family. (v) Isomerization reactions (IR): this class contains 2 families. (vi) Unclassified (UCs): this class contains 2 families. The family and class classifications were investigated by both SSN analysis (Fig. 3) and phylogenetic analysis (Fig. S1).

**TABLE 1:** Signature Pfam domains of 26 PUP families

Family	Signature Pfam domains	No. of seeds	No. of homologous proteins found with:		
			Swiss-Prot	TrEMBL	UHGP
FR1	Alpha-amylase	3	184	2,537	4,385
FR2	Glyco_hydro_70	2	6	758	1,534
FR3	Arylsulfotrans+Arylsulfotran_N	1	1	226	2,183
FR4	PTase_Orf2	1	5	70	0
OR1	ADH_zinc_N+ADH_N_2	1	706	4,350	1,991
OR2	Rieske+Ring_hydroxyl_A	1	40	2,456	64
OR3	Oxidored_FMN+Pyr_redox_2	6	8	3,588	2,729
OR4	FAD_binding_2	4	189	1,585	2,521
OR5	adh_short_C2	4	944	1,233	80
OR6	HpaB+HpaB_N	1	12	3,373	3,335
OR7	FMN_red	2	2	1,185	4,859
OR8	Cupin_2	2	252	3,671	1,526
OR9	NAD_binding_10	2	223	702	768
HR1	Glyco_hydro_1	3	197	1,326	1,386
HR2	Glyco_hydro_11	1	86	3,701	306
HR3	Glyco_hydro_3+Fn3-like+Glyco_hydro_3_C	5	105	4,825	3,979
HR4	AP_endonuc_2	3	518	1,235	2,245
HR5	GFO_IDH_MocA	3	191	1,190	4,785
HR6	Glyco_hydro_106	2	1	999	4,462
HR7	Bac_rhamnosid6H	6	0	3,715	3,950
HR8	DAPG_hydrolase	2	1	348	155
IR1	Glyoxalase_4	1	35	12	27
IR2	Chalcone_N	1	0	78	30
NCR1	Amidohydro_2	1	20	3,048	953
UC1		1	0	1,275	346
UC2		1	2,062	3,420	2,558

### We found 56,694 UniProt proteins to be homologs of the 60 PUP seeds.

To recruit homologs of the 60 seed proteins, we conducted a homology search against Swiss-Prot and TrEMBL databases (two components of UniProt; Materials and Methods). In total, 5,788 Swiss-Prot and 50,906 TrEMBL proteins were found to be homologs of the 60 PUP seeds. Table 1 shows the breakdown numbers of homologs for each of the 26 PUP families in the two databases. As these homologs were recruited as members of the 26 PUP families based on homology search (share either signature Pfam domains or full-length protein sequence similarity), they are putative candidates for future experimental characterization of new proteins in polyphenol utilization.

The Swiss-Prot proteins are highly reliable, as they have been curated by UniProt staff and often have experimental evidence to support their existence as real proteins and their functional annotation (18). To study the sequence relatedness among PUP seeds and their Swiss-Prot hits, we reconstructed a phylogeny for each of the 26 PUP families. The 26 PUP family phylogenies containing Swiss-Prot proteins are provided in Data set S1 and on our website. In contrast, the TrEMBL database contains >180 million computer-predicted proteins translated from coding sequences, and thus the confidence of TrEMBL proteins’ existence and annotation is low, as there is no evidence about their presence and functions. Moreover, the numbers of TrEMBL hits in the 26 PUP families are too big (Table 1) for reliable phylogeny reconstructions.

**FIG 4:**
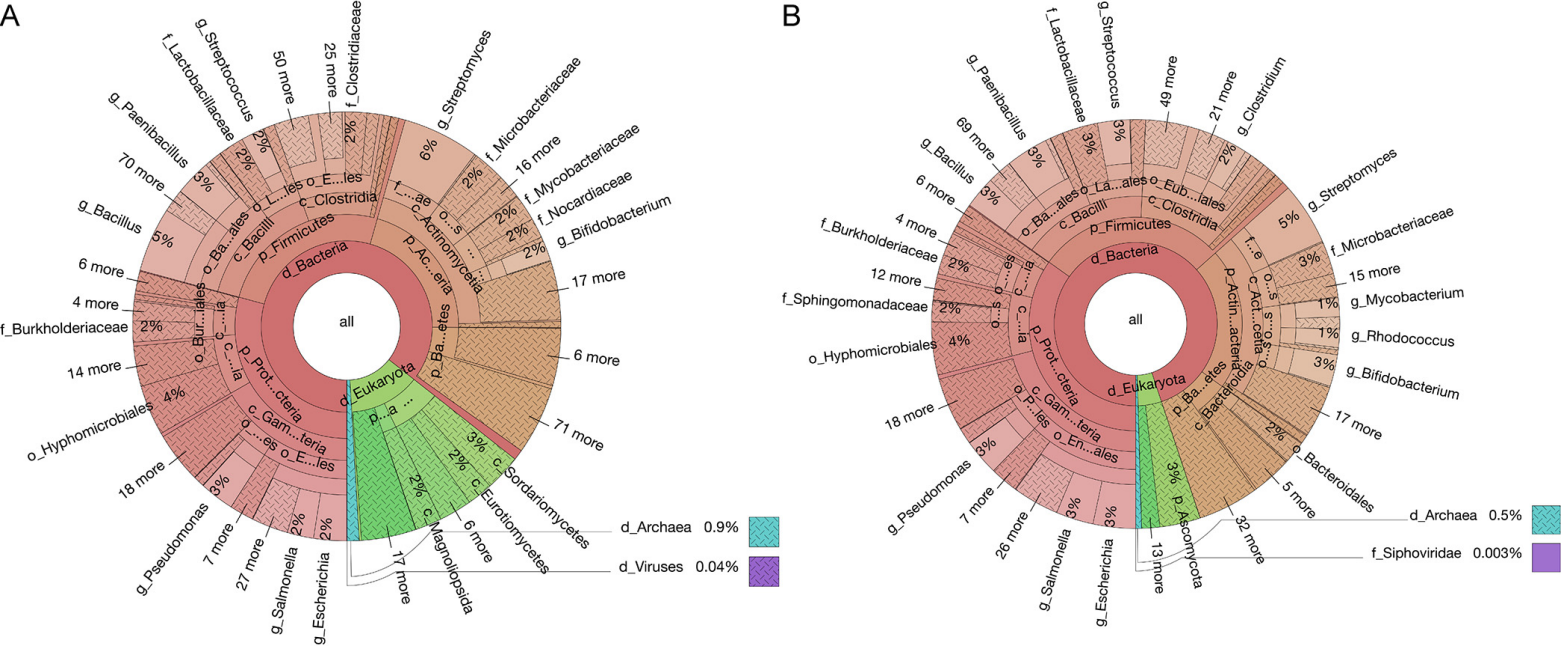
Krona taxonomy distribution plots of PUP homologs in UniProt. (A) UniProt homologs of all the 26 PUP families collectively. (B) UniProt homologs of 28 seed-containing subfamilies collectively.

To visualize the relatedness of TrEMBL hits to Swiss-Prot hits and PUP seeds, we built SSN for each family (see Materials and Methods). SSN graphs of the 26 PUP families containing Swiss-Prot plus TrEMBL plus seed proteins are provided in Data set S2 and on our website. Figure 4A shows the Krona taxonomy distribution (19) of all the 26 PUP families together, while the plots for each of the 26 families are provided in Data set S3. Although the 60 seed proteins are from only *Bacteria* and *Archaea* (Fig. 2), their UniProt homologs are also found in *Eukarya* (Fig. 4A). The four phyla that contain the most UniProt homologs are *Proteobacteria*, *Firmicutes*, *Actinobacteria*, and *Bacteroidetes* (this phylum is not found in the 60 PUP seeds, though; Fig. 2).

Studying the taxonomic distribution of the nodes (proteins) in SSNs revealed the phyletic distribution of the 26 PUP families: 22 PUP families were found in all life kingdoms (*Bacteria*, *Archaea*, and *Eukarya*) and thus are the most widely distributed. In contrast, PUP families FR2, FR3, and IR2 were found only in *Bacteria*. Among the three PUP families, family FR2 is mostly present in the *Lactobacillales* order of the *Firmicutes* phylum.

SSNs also allowed us to further classify UniProt proteins in each PUP family into subfamilies (Data set S2 and see Materials and Methods). In total, 168 subfamilies (containing >10 proteins) were defined using SSNs based on the selected E value thresholds for each family (Table S1), covering 47,738 (84.37%) proteins of, in total, 56,694 Swiss-Prot plus TrEMBL plus seed proteins in all 26 families. Among these subfamilies, the most interesting ones are those that contain seed proteins. In total, 28 subfamilies were found to contain 40 (66.67%) of the 60 PUP seeds; the rest (20) of the seeds were not found in subfamilies, as they were found in SSN clusters with <10 proteins or their families were not clustered into subfamilies. These 28 subfamilies contain the most confident PUP candidates, as they share significant sequence similarities with PUP seeds. The Krona taxonomic distribution plot of the 28 seed-containing subfamilies is shown in Fig. 4B. Although the 28 subfamilies contain only 19,849 (35.01%) of the 56,694 proteins, their taxonomic distribution is very similar to that of all the 56,694 proteins of the 26 families (Fig. 4A).

### We found 51,157 UHGP proteins to be homologs of the 60 PUP seeds.

A total of 51,157 PUP homologs were found in the UHGP-100 database (16). As mentioned in Materials and Methods, UHGP-100 proteins are predicted from genomes in the UHGG database, which contains 10,648 isolate genomes and 276,349 metagenome-assembled genomes (MAGs) from human gut microbiome. Each UHGG genome is linked to very useful metadata, particularly the taxonomic and geographical information, which allowed us to infer associations between the abundance and prevalence of PUP homologs and species taxonomy and host location (an indication of diet preference).

**FIG 5:**
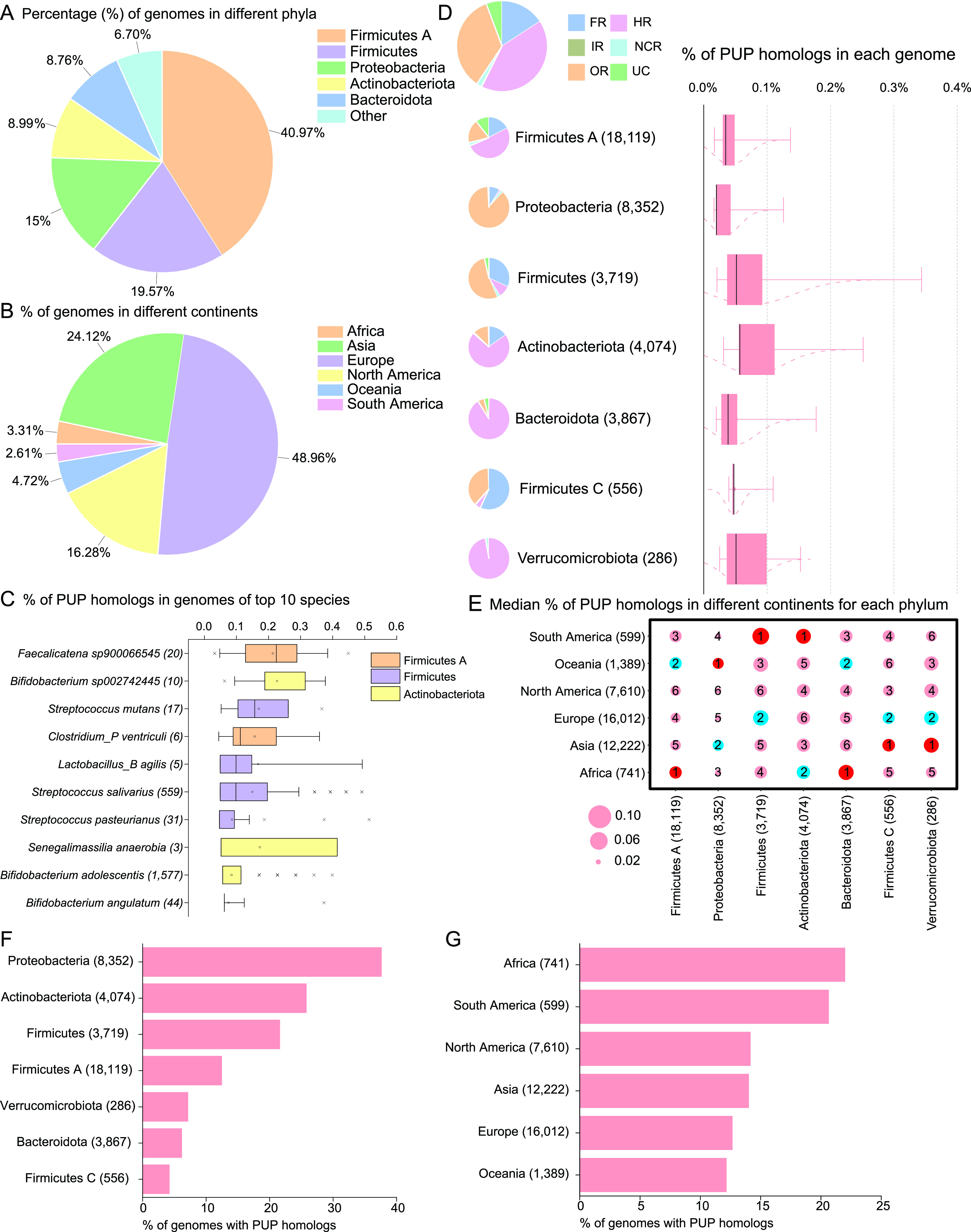
Analysis of 51,157 PUP homologs from UHGG. (A) Phylum-level taxonomic distribution and (B) continent-level geographical distribution of 39,296 PUP-containing genomes. (C) Top 10 UHGG species by the percentage of PUP homologs in the genomes of the species; UHGG species names are followed by the number of genomes in parentheses. (D) Pie charts and boxplots. Pie charts show the relative fraction of PUPs in different classes (class full names are provided in the main text); box plot shows the percentage of PUP homologs in genomes of different phyla. The numbers in parentheses are the numbers of genomes. (E) Bubble plot of the percentage of PUP homologs per genome in different phyla (*x* axis) across continents (*y* axis). The size of the bubbles represents the median of the percentage of PUP homologs per genome from specific phyla. The number inside the bubbles is the size rank in each column (phylum). The numbers in parentheses of *x* and *y* labels are the numbers of genomes. (F) The percentage of genomes containing PUP homologs across seven major phyla. (G) The percentage of genomes containing PUP homologs across different continents. The numbers in all the parentheses are the numbers of genomes.

The 51,157 PUP homologs are found in 39,296 UHGG genomes. Figure 5A shows that among these genomes, the most abundant phyla are *Firmicutes A* (40.97%), *Firmicutes* (19.57%), and *Proteobacteria* (15%). *Firmicutes A* to *Firmicutes I* are new phyla recently separated from the *Firmicutes* phylum by the Genome Taxonomy Database (GTDB) (21) and adopted by the UHGG database. Figure 5B shows the continent-level geographical distribution of these 39,296 genomes, with the top continents being Europe (48.96%), Asia (24.12%), and North America (16.28%).

Figure 5C shows the top 10 ranked UHGG species in terms of the percentage of PUP homologs in their genomes. These species are from *Firmicutes A*, *Firmicutes*, and *Actinobacteriota* phyla. Among the top 10 ranked species, *Faecalicatena sp900066545* has the highest median of percentage of PUP homologs in genomes (0.22%), followed by *Bifidobacterium sp002742445* with a median of 0.19%.

Figure 5D shows that the top phyla with the highest median PUP homolog percentages are *Actinobacteria*, *Firmicutes*, and *Verrucomicrobiota*. The pie charts also show that different phyla differ in terms of the dominant enzyme classes. For example, the hydrolysis reactions (HR) class is the major PUP class in *Actinobacteria*, *Bacteroidota* (synonym of *Bacteroidetes*), *Firmicutes A*, and *Verrucomicrobiota*, while the oxidation/reduction reactions (OR) class is dominant in *Proteobacteria* and *Firmicutes*.

To study which continent tends to have genomes with a higher percentage of PUP homologs, a bubble plot was made in Fig. 5E to show the median of the percentages of PUP homologs in genomes of different continents given a specific phylum. The corresponding boxplots are provided in Data set S4. It is interesting to note that Africa, Asia, and South America are each ranked the first in two phyla in terms of the percentages of PUP homologs in UHGG genomes. Oceania is ranked the first in one phylum, while in no phylum are North America and Europe ranked the first. This observation fits our expectation in that people in North America and Europe tend to have diets that are less plant derived than those of people in other continents.

Furthermore, Fig. 5F and G are made to study the prevalence of PUP-containing genomes. Prevalence is defined as the number of genomes containing at least one PUP homolog divided by the total number of genomes in each phylum or continent. In Fig. 5F, *Proteobacteria* has the highest prevalence, with 37.62% of its genomes having PUP homologs. In Fig. 5G, Africa and South America have the highest prevalence, with more than 20% of their genomes having PUP homologs. Taking consideration of both Fig. 5E and G, we conclude that the microbiome of African population has the highest genome prevalence and PUP abundance, which is expected given that African diets are richer in dietary polyphenols than are those of other continents.

### We found that 1,074 PGCs contained 2,742 physically linked PUP homologous genes in 989 UHGG genomes.

With all PUP homologs identified in UHGP-100 and located in the UHGG genomes (16), we have identified physically linked PUP gene clusters (PGCs) in the gut microbiome. The concept of PGCs is inspired by the success of polysaccharide utilization loci (PULs) (22) or CAZyme gene clusters (CGCs) for carbohydrate utilization (23, 24). The idea is that for more efficient polyphenol utilization, PUP-encoding genes might be clustered with each other in the microbial genomes to form an operon or physically linked gene clusters for coordinated gene expression. Clustered PUP genes have been reported (25–31), but the term “PUP gene clusters” (PGCs) has never appeared in the literature.

**FIG 6:**
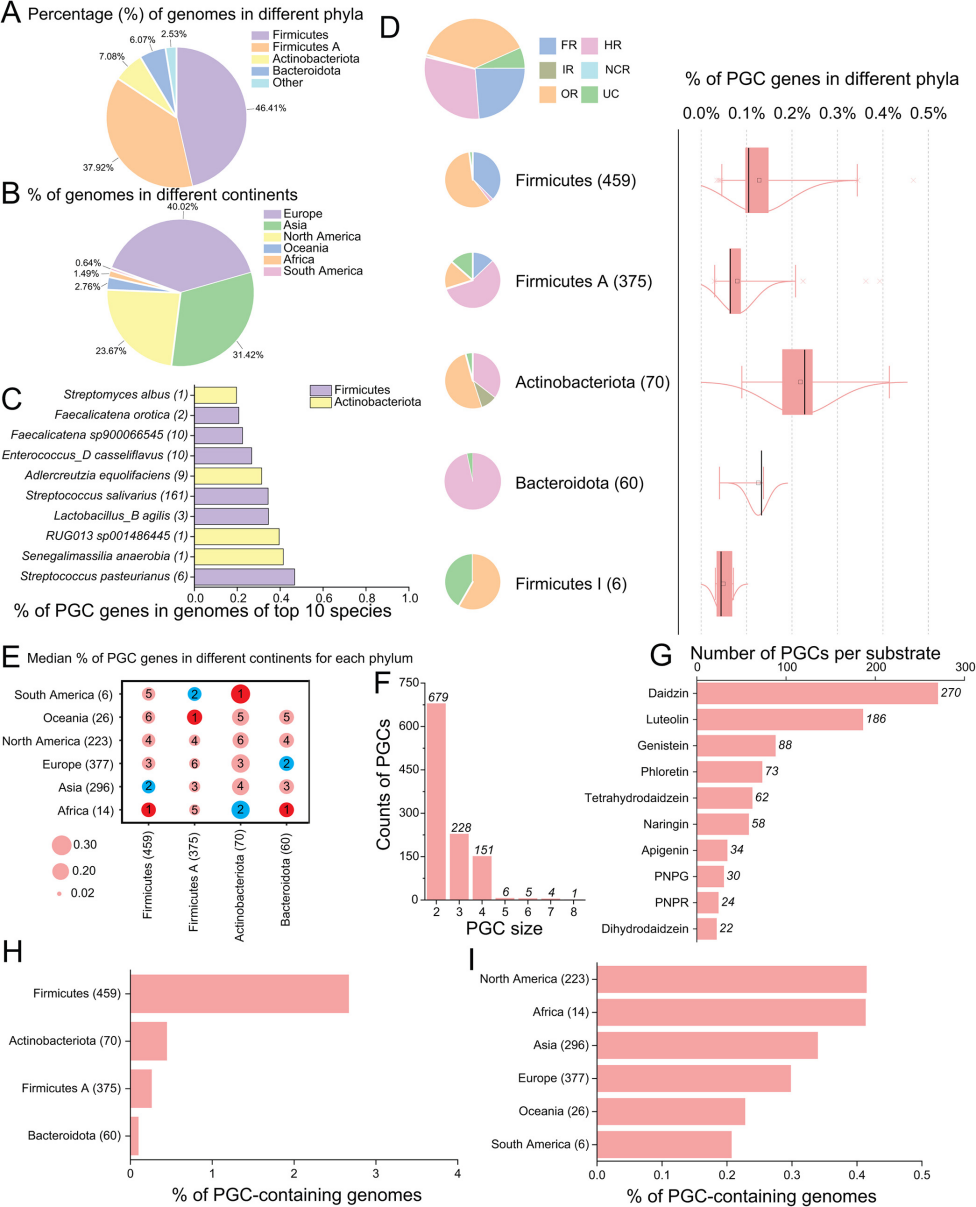
Analysis of 1,074 PGCs (2,742 PUP homologs) from 989 UHGG genomes. (A) The phylum-level taxonomic distribution and (B) the continent-level geographical distribution of 989 PGC-containing genomes. (C) Top 10 UHGG species by the percentage of PGC genes per genome; species names are followed by the number of genomes in parentheses. (D) Pie charts and boxplots. Pie charts show the relative fractions of different PUP classes; box plot shows the percentage of PGC genes per genome in different bacteria phyla. The numbers in parentheses are the numbers of genomes. (E) Bubble plot of the percentage of PGC genes per genome in different phyla (*x* axis) across continents (*y* axis). The size of the bubbles represents the median of the percentage of PGC genes per genome from specific phyla. The number inside the bubbles is the size rank in each column (phylum). The numbers in parentheses of *x* and *y* labels are the numbers of genomes. (F) The size distribution of PGCs. (G) The predicted substrate distribution of PGCs. PNPG, *p*-nitrophenyl-β-d-glucopyranoside; PNPR, *p*-nitrophenyl-α-l-rhamnopyranoside. (H) The percentage of genomes containing PGC genes across seven major phyla. (I) The percentage of genomes containing PGC genes across different continents. The number in all the parentheses is the number of genomes.

Using the criteria described in Materials and Methods, we extracted 1,074 PGCs from 989 UHGG genomes (i.e., PGC-containing genomes). Altogether 2,742 (5.4% of 51,157) PUP homologs were found in these PGCs. Figure 6A is a pie chart to show the phylum-level taxonomic distribution of PGC-containing genomes. Interestingly, the majority of the 989 PGC-containing genomes are from *Firmicutes* (46.41%) and *Firmicutes A* (37.92%). However, no PGC-containing genomes are from *Proteobacteria*, although *Proteobacteria* accounts for 15% of UHGG genomes containing PUP homologs (Fig. 5A).

Figure 6B is a pie chart to show the continent-level distribution of PGC-containing genomes. The distribution is similar to that shown in Fig. 5B, with the majority of genomes from Europe, Asia, and North America. Figure 6C shows the top 10 ranked UHGG species in terms of the percentage of genes located in PGCs. The species with the highest percentage of PGC genes is Streptococcus pasteurianus; each of its 6 genomes (e.g., UHGG genome ID: GUT_GENOME208791) contains 10 PGCs with 26 PUP homologous genes.

Figure 6D shows the boxplot of the percentage of PGC genes across all the taxonomic phyla. The phyla with the highest median percentages are *Actinobacteria*, *Bacteroidetes*, and *Firmicutes*, which largely mirrors what is observed for the percentage of PUP homologs in Fig. 5D. The pie charts show that in PGCs of *Actinobacteria* and *Firmicutes*, the dominant PUP genes are of OR class; however, in PGCs of *Firmicutes A* and *Bacteroidetes*, the dominant PUP genes are of HR class. Overall, in the 1,074 PGCs, OR genes are the most abundant PUPs, followed by HR and FR. At the PUP family level, 23.71% of the PGC genes belong to family OR7, followed by family FR2 (14.51%).

Figure 6E shows the bubble plot of the median of the percentage of genes located in PGCs in different major phyla across all the continents. The boxplots of the percentage of genes located in PGCs in each major phylum are provided in Data set S5. Like what was found for the percentage of PUP homologs, Africa is the continent with the highest percentages of PGC genes in the gut bacterial genomes, followed by South America and Oceania. This implies the importance of PGCs to the ingestion of polyphenol-rich diets.

Figure 6F shows the size distribution of PGCs. Clearly the majority of PGCs contain ≤4 genes, although we allowed up to 3 non-PUP genes inserted between 2 neighboring PUP genes. The PGC with the most (five) PUP genes is from a *Faecalicatena* species (UHGG genome ID: GUT_GENOME199169), which belongs to the *Firmicutes A* phylum. The predicted substrate of this PGC is daidzin. As a comparison, the largest PGC contains in total eight genes; among them, four are PUP genes. This PGC is from Senegalimassilia anaerobia (UHGG genome ID: GUT_GENOME035778) of the *Actinobacteriota* phylum. The predicted substrates of this PGC include genistein, tetrahydrodaidzein, and dihydrodaidzein, which was successfully identified in other equol-forming bacteria (25).

To learn more about the putative functions of PGCs, we used a Pfam domain-based strategy and a simple majority voting algorithm to infer the substrates for all 1,074 PGCs (see Materials and Methods). Figure 6G shows that daidzin has the most PGCs (270), followed by luteolin (186 PGCs) and genistein (88 PGCs). Among the three substrates, daidzin and genistein are both isoflavones commonly found in the bean family of plants (e.g., soybean, cowpea, coffee). Isoflavones are known to have antioxidant and anthelmintic functions, and some isoflavones (e.g., genistein) can be used as angiogenesis inhibitors to treat cancers. Daidzin is well-studied and can be utilized by gut microbiota to produce the bioactive S-equol (32). The main soybean isoflavone, genistein, can be converted to 5-hydroxy-equol (25).

For the prevalence of PGC-containing genomes, Fig. 6H and I show the percentage of genomes containing PGC genes in major phyla and continents, respectively. *Firmicutes* stands out, with 2.66% of its genomes containing PGC genes, which is over 6 times higher than that percentage for the other phyla. As for geographical distribution, North America and Africa have the highest percentages of genomes containing PGC genes, which differs from what was observed for the PUP homologs distribution across continents (Fig. 5G).

It should be noted that the identification of PGCs depends on the quality of the genome assembly. PGCs could be missed in more-fragmented genomes. Additionally, polysaccharide utilization loci (PULs) or CAZyme gene clusters (CGCs) for carbohydrate utilization (23, 24) have been experimentally studied for many years, which led to some clear rules to define a PUL/CGC. However, experimental evidence for PGCs is limited and rules to define PUP gene clustering have never been reported. The term “PGC” that is defined in this study will certainly need to be validated by the community. The algorithm/rules we used here will evolve as our experimental knowledge of PUP gene clustering improves in the future.

### Novel Pfam families were identified in PGCs representing putative new PUPs that need experimental validation.

As non-PUP homologs are allowed in PGCs, it would be interesting to study what functions these non-PUP homologs have, and those that are enriched in PGCs could be candidates of novel PUPs. This idea is known as guilt by association: genes cooccurring in the same genomic context across many genomes are likely involved in the same biological process. This gene neighborhood idea has been widely used in genome mining of various important genes involved in, for example, bacterial defense system (33), natural product synthesis (34), and carbohydrate degradation (22).

To study what Pfam domains are enriched in non-PUPs of PGCs, we identified Pfam domains for all the 507 non-PUP proteins in PGCs via HMMSCAN against the Pfam database. Table 2 shows the top 10 Pfam domains ranked by their occurrence in non-PUPs. To statistically test their overrepresentation in the 1,074 PGCs, a hypergeometry test was conducted on each of the 10 Pfam domains, with proteins in PGCs as the foreground and all the proteins of the 989 PGC-containing genomes as the background, following a method described in our previous papers (35–38). All these 10 Pfam domains have very significant *P* values, suggesting that compared to the genome background, they tend to appear in PGCs (or colocalized with known PUP homologs). Therefore, these Pfam domains represent novel PUP families that have not been known for polyphenol catabolism and are good candidates for future experimental validation.

**TABLE 2:** Novel Pfam domains found in non-PUP proteins of PGCs

Pfam domain	No. of non-PUP proteins	Enrichment *P* value
Choline_bind_3	110	8.91e−114
Choline_bind_1	106	9.93e−124
GFO_IDH_MocA_C	87	3.06e−94
TetR_N	58	7.07e−36
Gly_kinase	58	5.61e−84
Bac_rhamnosid_C	43	3.95e−54
Bac_rhamnosid_N	41	4.27e−51
Bac_rhamnosid	41	2.31e−51
ROK	38	2.68e−24
Lactamase_B	36	6.45e−18

Among the top 10 Pfam families, GFO_IDH_MocA_C appears in 87 non-PUPs (Table 2). Note that GFO_IDH_MocA is the signature domain of HR5 PUP family and AP_endonuc_2 is the signature domain of HR4 family (Table 1). In fact, GFO_IDH_MocA_C often cooccurs with GFO_IDH_MocA and AP_endonuc_2 in PUP homologs: 945 UniProt proteins and 4,913 UHGG proteins have AP_endonuc_2 plus GFO_IDH_MocA_C or GFO_IDH_MocA plus GFO_IDH_MocA_C domain architecture. There are also 500 proteins with AP_endonuc_2 plus GFO_IDH_MocA plus GFO_IDH_MocA_C domain architecture in PGCs. This domain architecture has been investigated (26) in intestinal bacteria *Lachnospiraceae* strain CG19-1 and Eubacterium cellulosolvens, which are able to catalyze the *O*-deglycosylation of flavonoid *O*-glucosides (e.g., daidzin). This implies a conserved Pfam domain architecture for the deglycosylation of polyphenols. Choline_bind_3 and Choline_bind_1 (Table 2) are both choline-binding repeats and usually cooccur in the same proteins; they are commonly found in glucosyltransferases and within the glucan-binding domain (39). The Bac_rhamnosid domain (Table 2) is found in rhamnosidase A and B enzymes and is involved in the hydrolysis of glycosides, which is the first step for the conversion of polyphenols in human gut. Thus, all these discussed domains have strong evidence to be involved in polyphenol catabolism.

### dbPUP website provides data browsing and BLAST search utilities.

**FIG 7:**
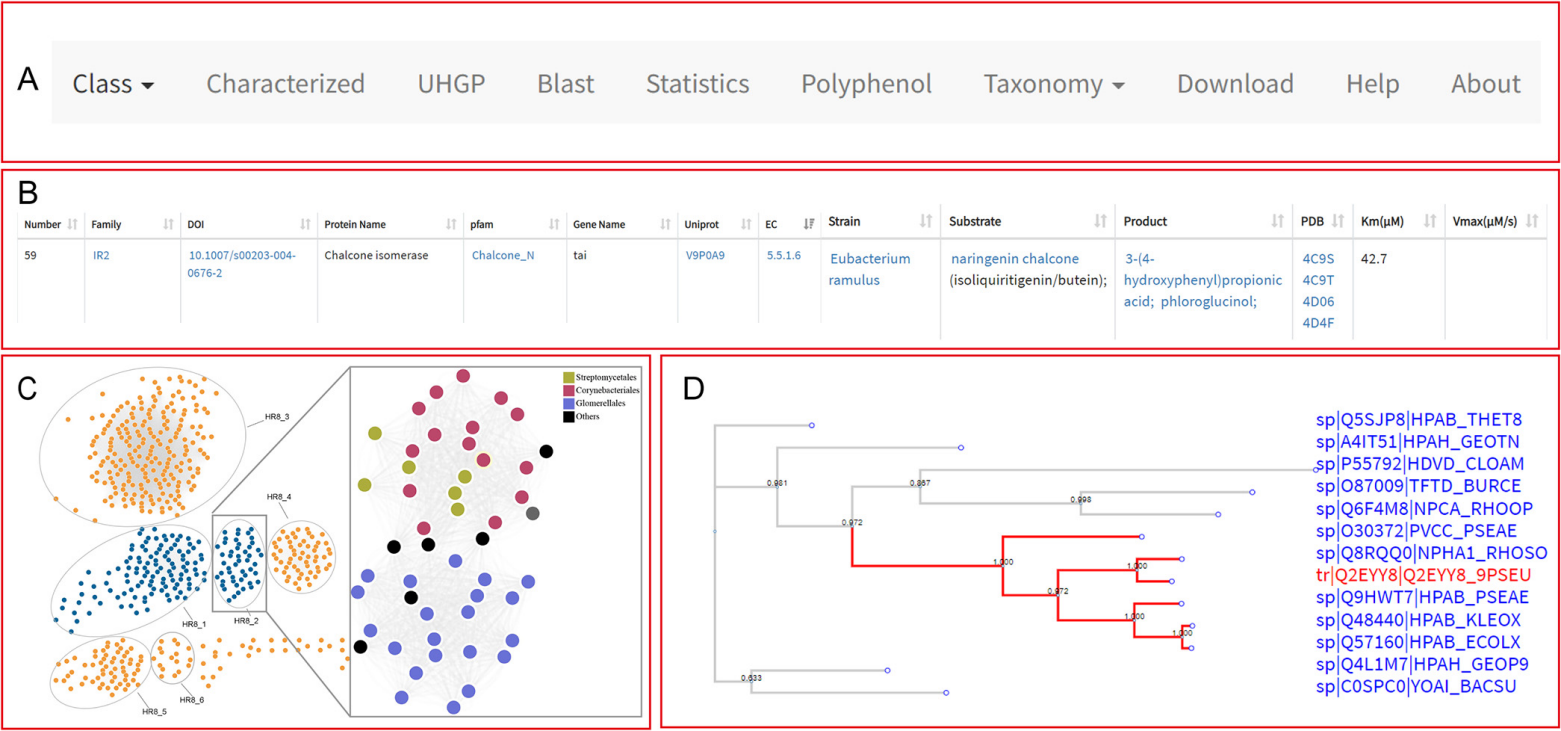
Overview of dbPUP website. (A) The navigation area provides quick links to different pages. (B) “Characterized” page (https://bcb.unl.edu/dbpup/characterized) of experimentally validated seed PUPs and associated metadata. (C) An example sequence similarity network (HR8 family: https://bcb.unl.edu/dbpup/network/HR8); subfamilies are shown in ovals, while nodes not in ovals are unclassified. Nodes in blue color indicate subfamilies containing seeds, while nodes in orange color indicate that there are no seed in the subfamily. A magnified view of subfamily HR8_2 is provided with nodes filled with different colors indicating their taxonomic groups at the order level. (D) Maximum-likelihood phylogeny (https://bcb.unl.edu/dbpup/tree/OR6) for Swiss-Prot homologs and seeds (red font).

The most important function of the dbPUP website (https://bcb.unl.edu/dbpup/) is to browse and search the experimentally verified PUP seed proteins and their homologs in UniProt and UHGG databases. The homepage of dbPUP website presents an overview about the definitions and terminology used in dbPUP, as well as the distribution of seeds among different enzyme classes. Through the navigation bar on the top of the homepage, users can be linked to different pages: (i) the pages of the six PUP classes, (ii) the characterized seed protein page, (iii) the page of the PUP homologs in UHGP, (iv) the BLAST page, (v) the data statistics page, (vi) the taxonomy distribution page of UHGP and UniProt homologs, (vii) the batch download page, (viii) the help page, and (ix) the about page (Fig. 7A).

Taking the “characterized” page as an example (Fig. 7B), users can access the experimentally validated seed PUPs in an interactive table, which includes all the important metadata of each protein and can be redirected to other pages. From the pages of the six PUP classes, users can find the detailed information for each enzyme class and further navigate to the webpage of each family (e.g., OR1) contained in the class and then to the webpage of each subfamily (e.g., OR1_1) contained in the family. From the family/subfamily pages, users can access the tables of UniProt homologs and view the SSN (Fig. 7C) and phylogeny (Fig. 7D) of each family.

The UHGP page is particularly useful for human microbiome researchers, where they can access the PUP homologs and PGCs in genomes of different continents (e.g., Africa or Europe). Lastly, from the BLAST page, users can query both nucleotide and protein sequences against all proteins in dbPUP. The results are returned as a webpage with a tabular report containing the hits in the databases, identity, E value, etc.

### The utility of dbPUP is supported by a case study.

**FIG 8:**
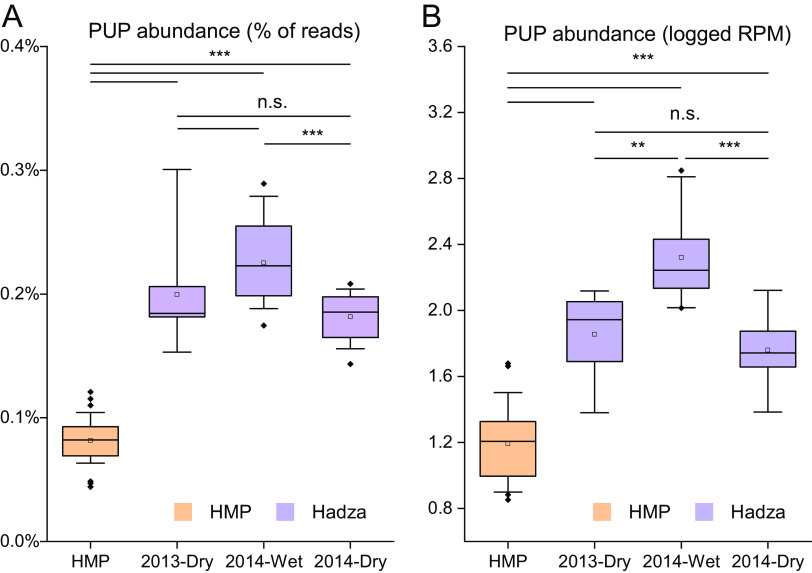
Comparing the PUP abundance between microbiomes of Hadza hunter-gatherers (different seasons) and those of Americans (HMP). (A) The PUP abundance is measured by the percentage of reads mapped to 60 experimentally characterized PUPs using BLASTX. (B) The PUP abundance is measured by the RPM values calculated by mapping reads to PUP homologs, which were identified by HMMSEARCH and PSI-BLAST search against metagenome-assembled genomes (MAGs). Details can be found in Materials and Methods. **, *P* < 0.01 and ***, *P* < 0.001 as determined by *t* tests; n.s., not significant.

Our global analysis of the UHGG MAGs (Fig. 5E and G) has suggested a potential link between the abundance of PUPs contained in human gut microbiome and the dietary choices of the host (using geographical location as an indicator). To further investigate this link and test the utility of dbPUP in analyzing metagenome data, we compared the PUP abundance between gut microbiome (non-Westernized diet) of African Hadza hunter-gatherers and gut microbiome (Westernized diet) of Americans (see Materials and Methods) (40). The PUP abundance was measured by using two different methods: (i) percentages of reads mapped to 60 experimentally characterized PUPs using BLASTX (Fig. 8A) and (ii) reads per kilobase per million mapped reads (RPM) values by mapping reads to PUP homologs identified by HMMSEARCH and PSI-BLAST (Fig. 8B). Regardless of methods, we observed that the Hadza hunter-gatherers possess a significantly greater functional capacity for utilization of polyphenols than the Americans due to different diet choices. Furthermore, within the Hadza hunter-gatherer samples, the abundance of PUPs was significantly higher in wet season samples than in dry season samples. This can be perfectly explained by the dietary changes between seasons: the Hadza hunter-gatherers consume more berries (polyphenol-rich diet) in the wet season and more meat in the dry season. Overall, this case study verified the feasibility of using dbPUP to profile metagenomic data for biologically meaningful discovery and supported the association between the host dietary choices and the abundance of PUPs contained within the microbiome.

### Conclusions.

Characterizing new polyphenol utilization proteins (PUPs) in human gut microbiome can be assisted by a deeper understanding of known PUPs and their sequence homologs. dbPUP is a comprehensive database for polyphenol utilization proteins. It contains 60 experimentally characterized PUPs (seeds), which are classified into 26 PUP families of 6 classes. Over 100,000 sequence homologs are also collected for the 26 PUP families from UniProt and UHGP databases. The functional description and experimental characterization method for each seed PUP are extracted from literature and provided on a user-friendly website, where users can browse, search, and download all the data. A total of 1,074 physically linked PGCs are identified by examination of the genomic location of 51,157 PUP homologs in 989 human gut microbiome genomes. An in-depth analysis of these PUP homologs and PGCs reveals a higher abundance and prevalence in African populations, supporting the notion that dietary polyphenols play a significant role in modulation human gut microbiome composition and functional configuration. We plan to update dbPUP annually to include new biochemically characterized PUPs from the literature. We expect dbPUP to be of great interest to researchers working on experimentally validating new PUPs using candidates in dbPUP.

## MATERIALS AND METHODS

### PUP seed collection via literature curation.

Biochemically characterized PUPs (termed seeds) were retrieved from literature search via keyword queries of PubMed and BRENDA database (Fig. 1):

(i) Keyword query in PubMed: “(microorganism [Title/Abstract]) AND (polyphenols [Title/Abstract])” and “(flavonoids [Title/Abstract]) AND (microorganism [Title/Abstract])” and “(microorganism [Title/Abstract]) AND (non-flavonoids [Title/Abstract])”
(ii) Keyword query in BRENDA: Organism → select “Prokaryotes”), then search in text fields (substrate → type in SPECIFIC POLYPHENOL).

The substrate keywords for the “SPECIFIC POLYPHENOL” search in BRENDA are provided in the Appendix. As the result, we obtained 60 experimentally characterized PUP seeds. Papers and BRENDA webpages of these seeds were manually scrutinized to collect detailed information, such as species, gene name, UniProt ID, EC number, protein structure, and biochemical reaction kinetic parameters. All the 60 seeds must have biochemical evidence to support their activities on specific polyphenols (i.e., have known substrates and products).

### Classification of PUP seeds using EC class, Pfam domain, SSN, and phylogenetic analyses.

The 60 seed PUPs were first classified into six enzyme classes according to the first level (the first digit) of their EC numbers.

To further classify PUP classes into families, Pfam domains of the 60 seeds were analyzed (41). The HMMSCAN command of HMMER (42) was used to search the 60 seed proteins against the Pfam database to predict functional domains (Fig. 1). Domain hits with an E value of <1e−5 and coverage (alignment length/HMM length) of >0.3 were kept.

Next, an all-versus-all BLASTP search was performed for the 60 seed proteins. All the protein-protein pairs with E values of <1e−5 were used as the input to generate an SSN (sequence similarity network) (Fig. 3). This network contains all the 60 seed proteins as nodes and the connections between them as links, suggesting that they are protein-protein homologous pairs. Manually inspecting the SSN and considering the Pfam domain architecture allowed us to classify 58 seeds into 24 families (including singletons). Each family has a single Pfam domain or a multidomain combination as its signature (Table 1). Two seed proteins without Pfam domains were defined as two unclassified families. Therefore, the 60 seeds formed 26 families.

In addition, to depict the phylogenetic relationship among the 60 seed proteins, a multiple sequence alignment (MSA) of their full-length sequences was built by MAFFT (20). Then, FastTree was run on the MSA to build a phylogenetic tree (43). Even though not all the 60 proteins are homologous to each other, importantly, the full-length sequence phylogeny was used to assist the above SSN to group the 60 seed proteins into 26 families with their Pfam domain architectures shown alongside the phylogeny (Fig. S1).

To select the signature Pfam domain or multidomain combination to represent each seed family, the following criteria were used:

(i) If a seed protein contains only one single Pfam domain, that domain is selected as the signature
(ii) If a seed protein contains multiple Pfam domains and its other family members also contain the same multidomain architecture, that shared multidomain combination is selected as the signature
(iii) If a seed protein contains multiple Pfam domains but its other family members contain only one shared domain, this single shared domain is selected as the signature
(iv) In all these cases, keep only the Pfam domains that are described as enzymatic domains

### PUP homologs in UniProt and UHGP.

Once the 26 PUP families were defined, each was further expanded to include sequence homologs of seeds from the UniProt database (consists of Swiss-Prot and TrEMBL) (18) and the database of unified human gastrointestinal protein (UHGP) catalog (16). Specifically, for the 24 families with signature Pfam domain or multidomain combination (Table 1), homologs were collected by HMMSEARCH of Pfam domain models against UniProt and UHGP (E value < 1e−5, domain coverage > 0.6); for the two families without Pfam domains, PSI-BLAST of the seed proteins against UniProt and UHGP was performed instead (E value < 0.001, iterate until convergence). For families with the multidomain combination as their signatures, all the domains in the combination must be present in the homologs.

Additionally, because the TrEMBL database (unlike Swiss-Prot) is a database of protein sequences (>180 million proteins) computer-translated from the EMBL nucleotide database and contains numerous sequence redundancies, homologs from TrEMBL were further filtered to remove those that do not share significant homology with the seed proteins. Specifically, for each seed family, all its TrEMBL homologs (from Pfam search) were used as the database for a PSI-BLAST search with the family’s seed proteins as the query.

PUP homologs in UHGP-100 (>170 million proteins) were identified using the same process as that described above for TrEMBL homologs.

### PGCs in UHGP.

The UHGP database was derived from the unified human gastrointestinal genome (UHGG) database, which contains 204,938 nonredundant prokaryotic genomes from the human gut microbiome (16). UHGG genomes were built from different data sets (16, 44–51) and contain not only isolate genomes but also metagenome-assembled genomes (MAGs). UHGP contains all the proteins predicted in UHGG genomes. By locating the PUP homologs of UHGP in the genomes, we have identified PGCs in the gut microbiome, which are potentially involved in polyphenols utilization in human gut in a synergistic manner. PGCs are defined using the following algorithm: (i) at least two PUP homologs in the gene cluster, (ii) no more than three other genes are allowed between two adjacent PUP homologs, and (iii) all the intergenic lengths are less than 1 kb.

To infer substrates for PGCs, for each PGC, we first looked at its PUP genes, whose family membership we know according to the Pfam domain assignment. If we know its family, we know what seed proteins the family has (Table 1). Seed proteins have known substrates, so we can assign the substrates to PUP families and then to all PUP genes in the PGC. As mentioned above, one PGC must contain at least two PUP genes. If the PUP genes are from the same substrate group, the PGC is inferred to target that substrate. If the PUP genes are from multiple substrate groups, we look at which substrate has the most PUPs in the PGC, and then PGC is inferred to target that substrate. If two substrates have an equal number of PUPs, the PGC is inferred to target both substrates.

### Visualization and subfamily classification of UniProt homologs.

For each family, PUP homologs from Swiss-Prot were aligned by MAFFT, and then the resulting MSA was used to construct a phylogenetic tree by FastTree. For many families, the numbers of TrEMBL homologs are too large (Table 1) to visualize as phylogenies. Therefore, sequence similarity network (SSN) was used instead. Specifically, for each family, all the seeds plus Swiss-Prot plus TrEMBL homologs were subject to an all-versus-all BLASTP search. Depending on families, different E value thresholds were applied to filter the BLAST results (ranging from 10^−5^ to 10^−180^; Table S1). This is because different families have different levels of sequence heterogeneity, meaning using a same E value threshold will lead to some families not being able to be separated into clusters while others separate into too many clusters.

Using the filtered BLAST results as the inputs, SSNs for each family were built and visualized by Cytoscape using the yFiles organic layout (52). From the SSNs, subfamilies (clusters) were manually identified: a sequence cluster with at least 10 well-connected sequences was defined as a subfamily.

### Web development.

The user-friendly web interface of dbPUP was designed using MySQL (version 5.7.22), Flask (version 1.1.2), and Bootstrap (version 3.3.7), hosted on an Apache HTTP server (version 2.4.27) on a Ubuntu 17.10 computer. Users can browse the webpage (https://bcb.unl.edu/dbpup/) using Google Chrome, Mozilla Firefox, and Safari web browsers for a better experience.

### dbPUP utility case study.

We downloaded the assembled MAGs and the raw metagenomic reads of the gut microbiome of African Hadza hunter-gatherers (40) from https://opendata.lifebit.ai/table/SGB (47) under the study name SmitsSA_2017 (total 35 samples). As the control, we also downloaded MAGs and raw reads of 32 American Human Microbiome Project (HMP) metagenomic samples, which were also analyzed in SmitsSA_2017 (40) for carbohydrate metabolism. We performed two types of searches for PUP annotation, (i) BLASTX of metagenomic cleaned reads against the 60 experimentally characterized PUPs to calculate the percentages of reads mapped to PUPs and (ii) HMMSEARCH and PSI-BLAST search MAGs for PUP homologs, and then map cleaned reads back to PUP homologs to calculate the RPM values (reads per kilobase per million mapped reads) using Bowtie2 and SAMtools (53, 54).

## Appendix

The following compounds were used as the substrates in BRENDA database search: anthocyan, apigenin, apiin, astilbin, astragalin, avicularin, baicalein, bavachin, biochanin, bonannione, butein, catechin, chalcone, chrysin, chrysoeriol, cinnamtannin, cirsilineol, cirsimaritin, citrifolioside, cyanidin, daidzein, daidzeol, daidzin, delphinidin, didymin, diosmetin, diosmin, distylin, ellagic acid, eriocitrin, eriodictyol, eupatorin, flavane, flavanol, flavanone, flavin, flavone, flavonoid, flavonol, flavylium, formononetin, galangin, gardenin, genistein, genisteol, genistin, geraldone, glycitein, glycitin, hesperetin, hesperidin, hispidulin, hyperin, hyperoside, ideain, jaceidin, jaceosidin, kaempferide, kaempferol, keracyanin, kuromanin, luteolin, malvidin, malvin, mimulone, morin, myricetin, naringenin, narirutin, neochanin, nepetin, nicotiflorine, nobiletin, orientin, patuletin, pebrellin, pelargonidin, peonidin, petunidin, phloretin, phloridzin, pigment, pinocembrin, pinoresinol, pinoresinol, pinotin, poncirin, pratensol, pratol, prunetol, prunin, quercetin, quercetrin, quercitrin, resveratrol, reynoutrin, rhamnetin, rhoifolin, rutin, sakuranetin, scolymoside, scutellarein, sinensetin, skrofullein, sophoricol, spinacetin, spiraeoside, Substrate, tangeretin, taxifolin, tetramerd, trifolin, vicenin, vitexin, vitisin, xanthohumol.
