# Supplementary material for: Polyphenol Utilization Proteins in the Human Gut Microbiome
Source: Appl Environ Microbiol. 2022 Feb 8;88(3):e01851-21. doi: 10.1128/aem.01851-21 (PMC8824206; doi:10.1128/aem.01851-21)
Supplement: Supplemental file 1 — Table S1, Fig. S1, Data S1 to S5. Download aem.01851-21-s0001.pdf, PDF file, 12.3 MB [file aem.01851-21-s0001.pdf]

Table S1: E-value thresholds for subfamily classification

Family ID	Signature Pfams	E-value threshold
FR1	Alpha-amylase	10^{-120}
FR2	Glyco_hydro_70	10^{-120}
FR3	Arylsulfotrans + Arylsulfotran_N	10^{-120}
FR4	PTase_Orf2	10^{-5}
OR1	ADH_zinc_N + ADH_N_2	10^{-120}
OR2	Rieske + Ring_hydroxyl_A	10^{-120}
OR3	Oxidored_FMN + Pyr_redox_2	10^{-180}
OR4	FAD_binding_2	10^{-60}
OR5	adh_short_C2	10^{-40}
OR6	HpaB + HpaB_N	10^{-180}
OR7	FMN_red	10^{-50}
OR8	Cupin_2	10^{-50}
OR9	NAD_binding_10	10^{-120}
HR1	Glyco_hydro_1	10^{-120}
HR2	Glyco_hydro_11	10^{-100}
HR3	Glyco_hydro_3 + Fn3-like + Glyco_hydro_3_C	10^{-130}, identity > 50%
HR4	AP_endonuc_2	10^{-50}
HR5	GFO_IDH_MocA	10^{-50}
HR6	Glyco_hydro_106	10^{-120}
HR7	Bac_rhamnosid6H	10^{-150}, identity > 60%
HR8	DAPG_hydrolase	10^{-80}
IR1	Glyoxalase_4	10^{-120}
IR2	Chalcone_N	10^{-120}
NCR1	Amidohydro_2	10^{-90}
UC1	ND	10^{-50}
UC2	ND	10^{-150}

ND: not detected

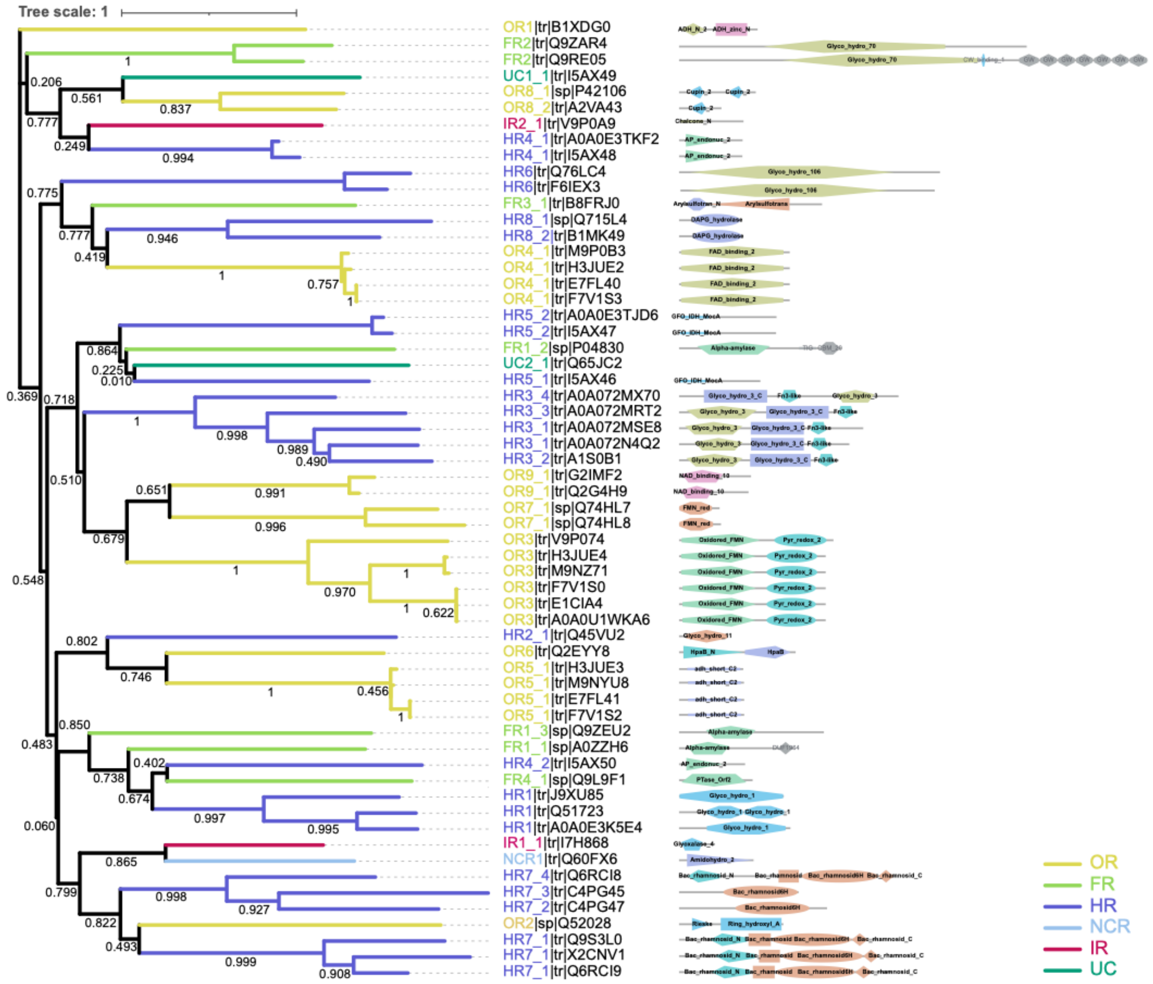

Figure S1. Phylogenetic relationship and domain architecture of seeds. The phylogeny is an approximately-maximum-likelihood tree reconstructed based on full-length sequence alignment of 60 seed proteins. Domain architecture was shown alongside the protein ID. Domains are represented by different shapes with different colors and grey means Pfam domains that are not signature domains.

Data S1 can be found at https://doi.org/10.6084/m9.figshare.17078294.v1

Data S2

From the SSNs, subfamilies (clusters) were manually identified: a sequence cluster with at least ten well-connected sequences was defined as a subfamily (e.g., FR1_1). For better presentation, subfamilies containing at least one seed protein were colored in blue, while those without seeds were colored in orange.

FR1

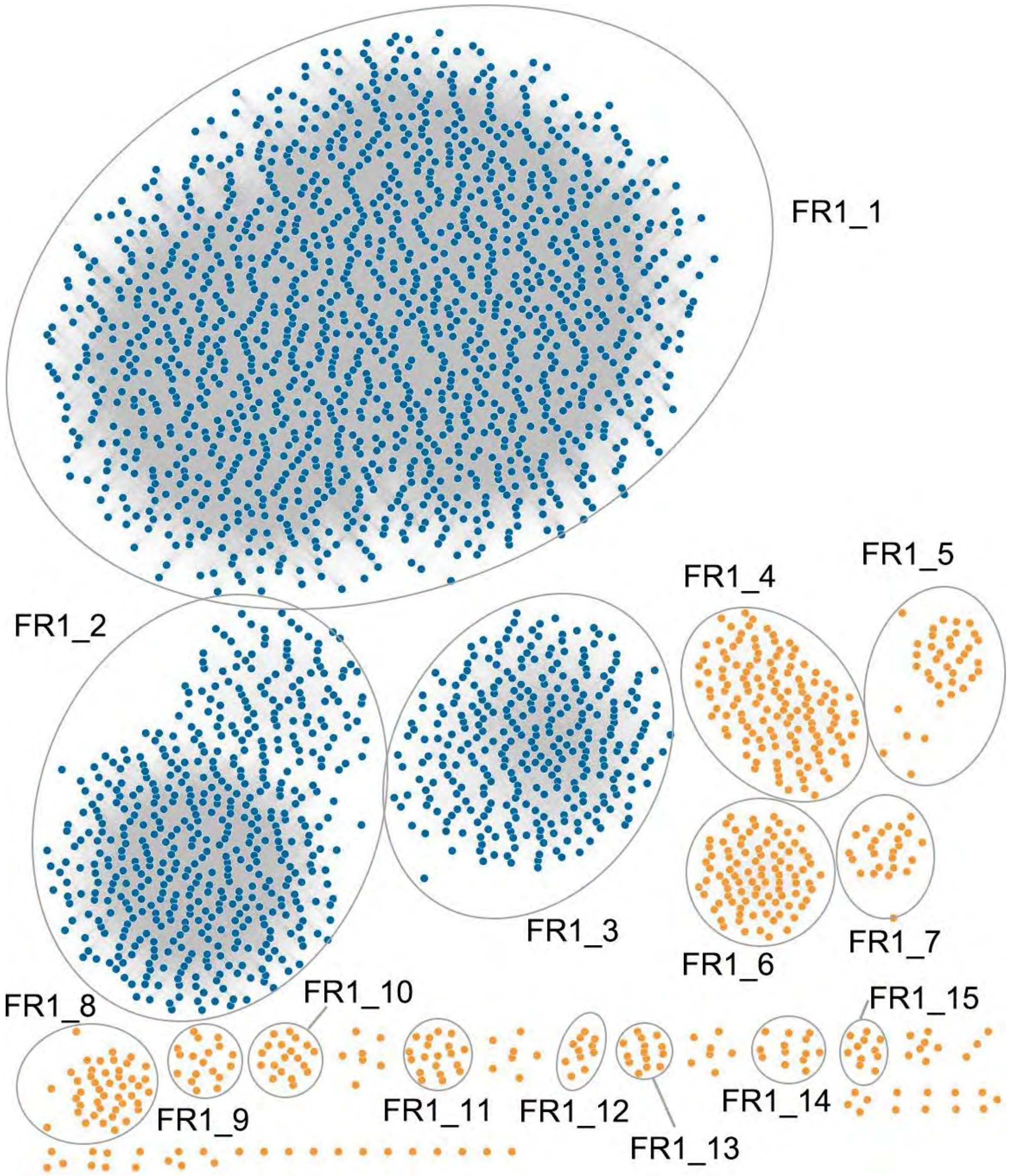
FR1_1
FR1_2
FR1_3
FR1_4
FR1_5
FR1_6
FR1_7
FR1_8
FR1_9
FR1_10
FR1_11
FR1_12
FR1_13
FR1_14
FR1_15

FR2

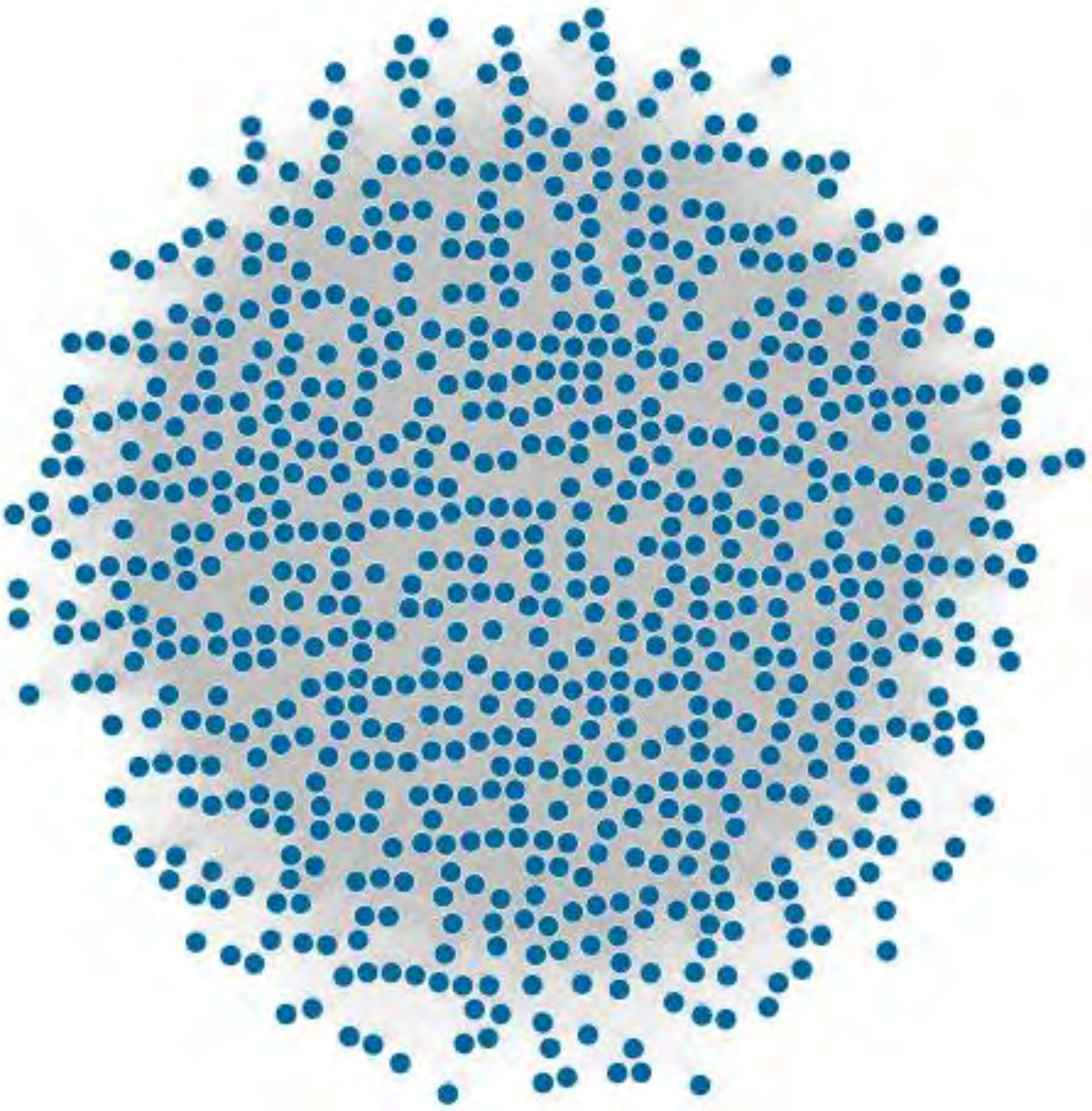

FR3

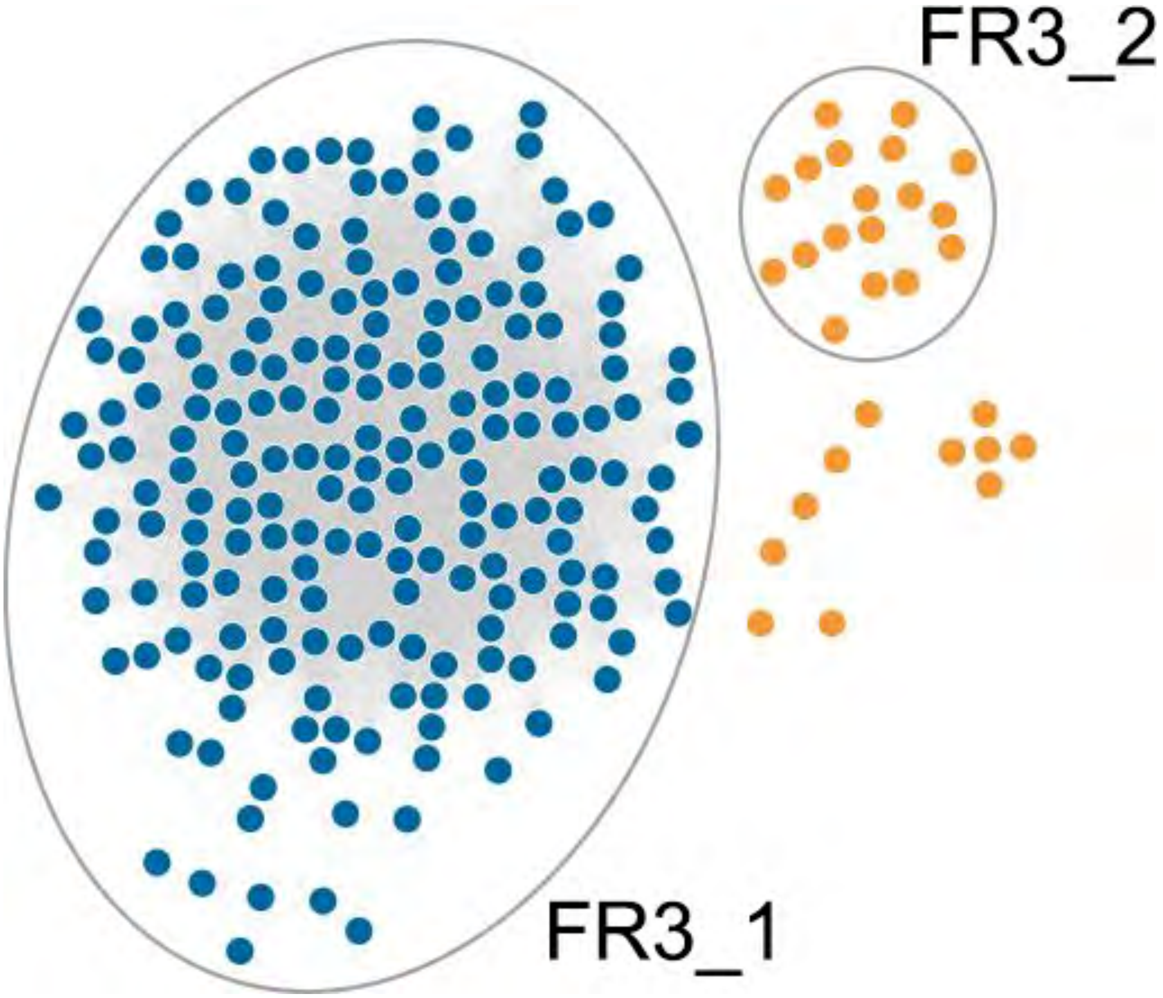

FR4

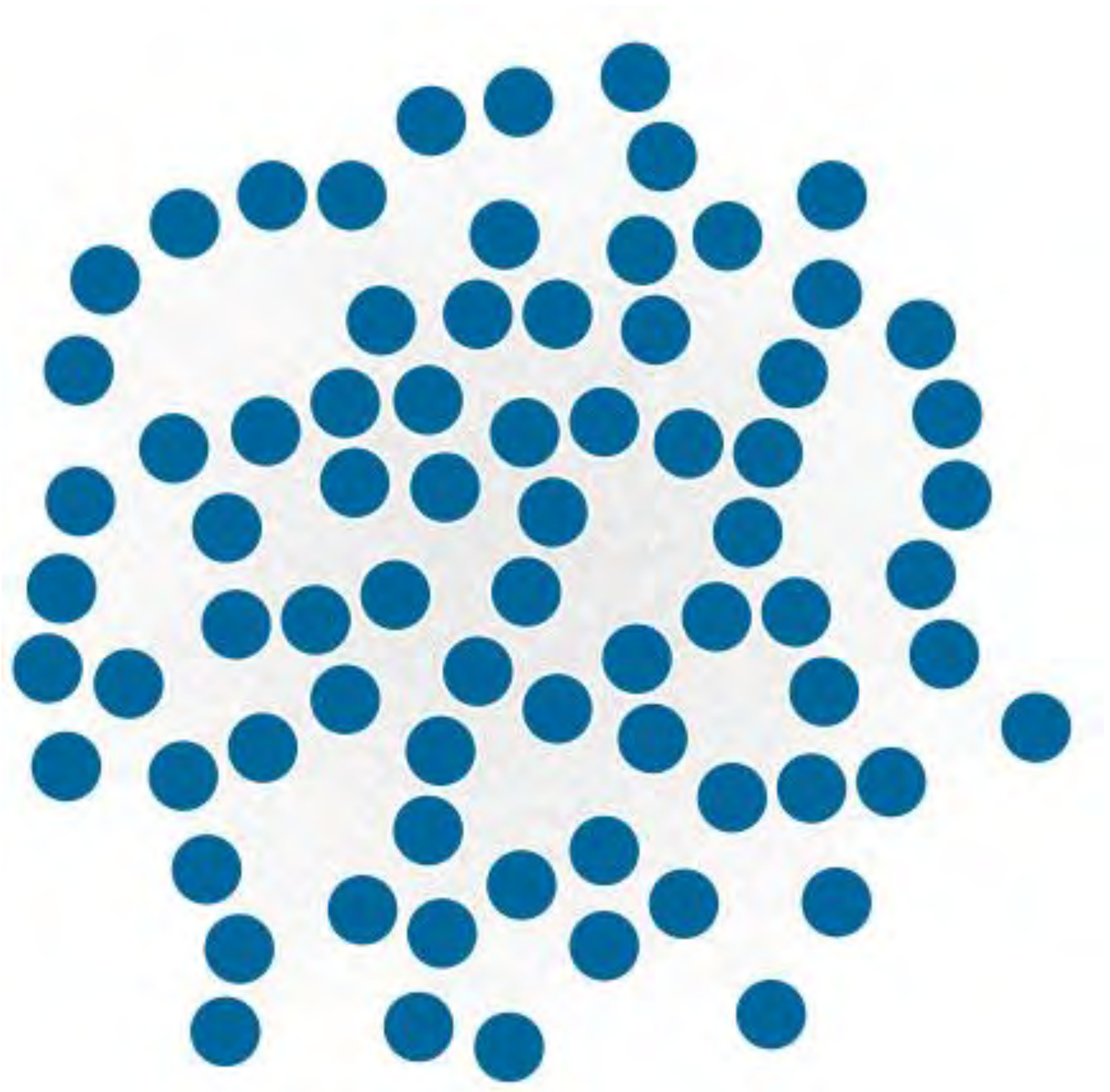

HR1

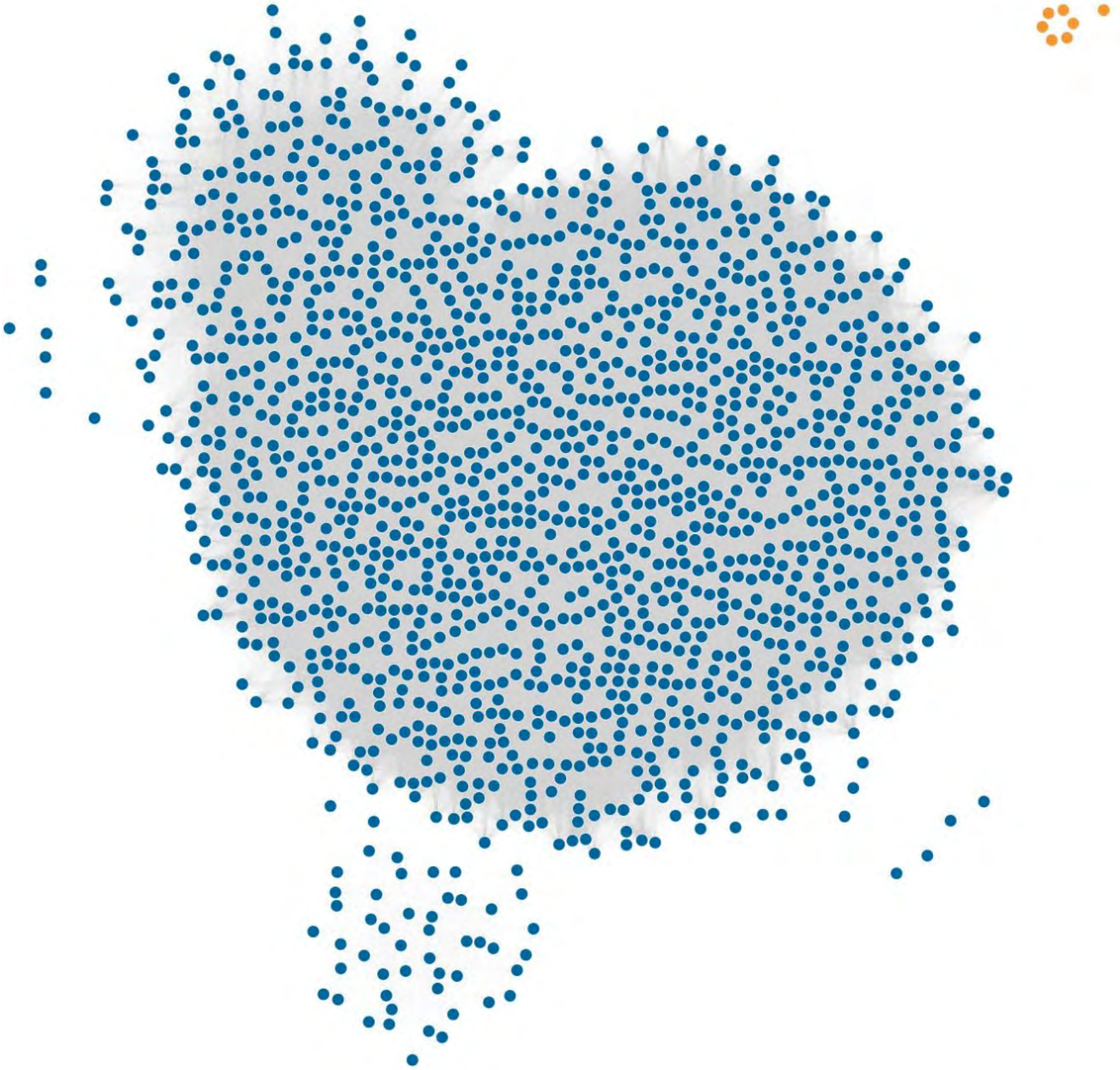

HR2

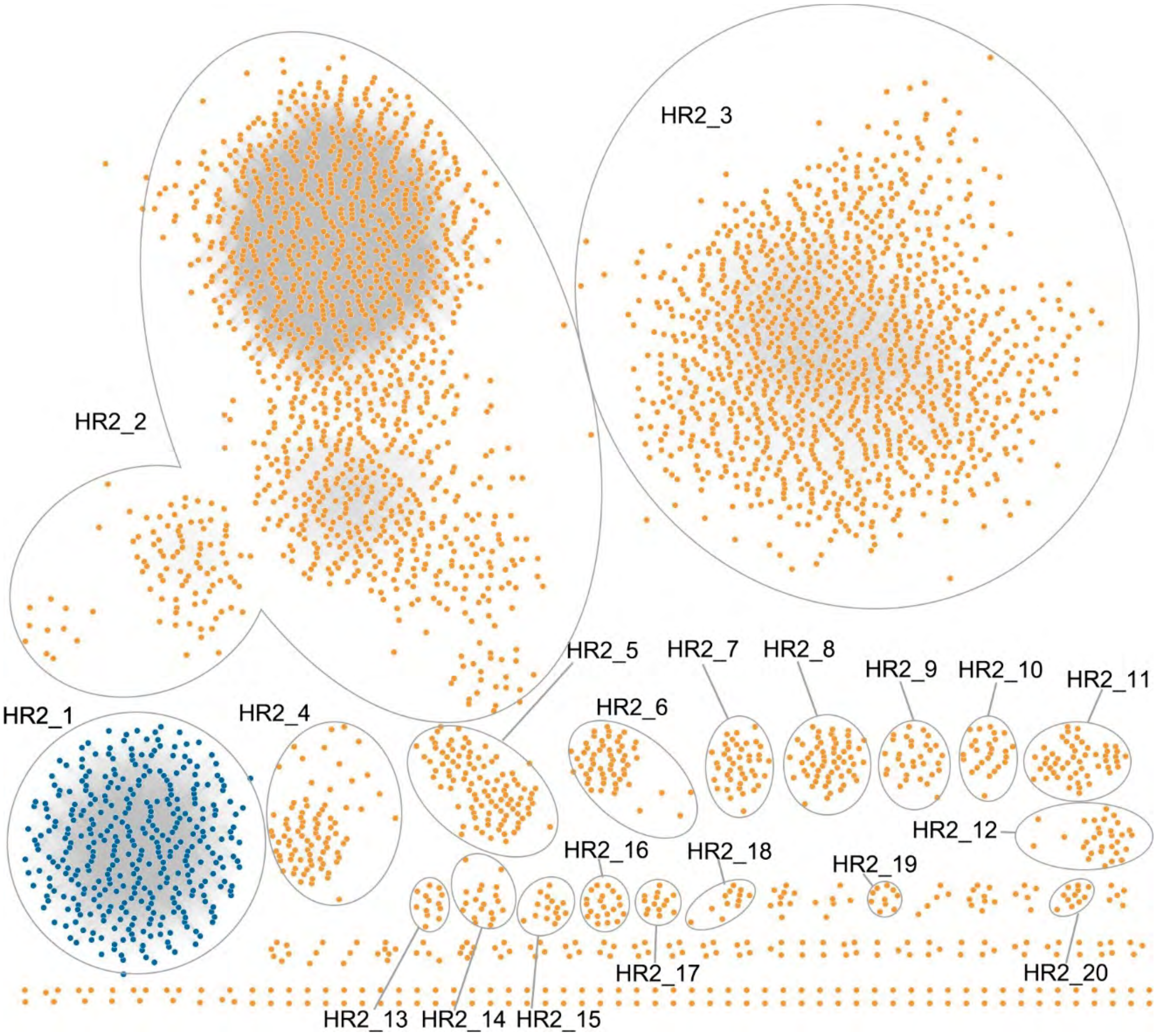

HR2_3
HR2_2
HR2_1
HR2_4
HR2_5
HR2_6
HR2_7
HR2_8
HR2_9
HR2_10
HR2_11
HR2_12
HR2_16
HR2_18
HR2_19
HR2_17
HR2_20
HR2_13
HR2_14
HR2_15

HR3

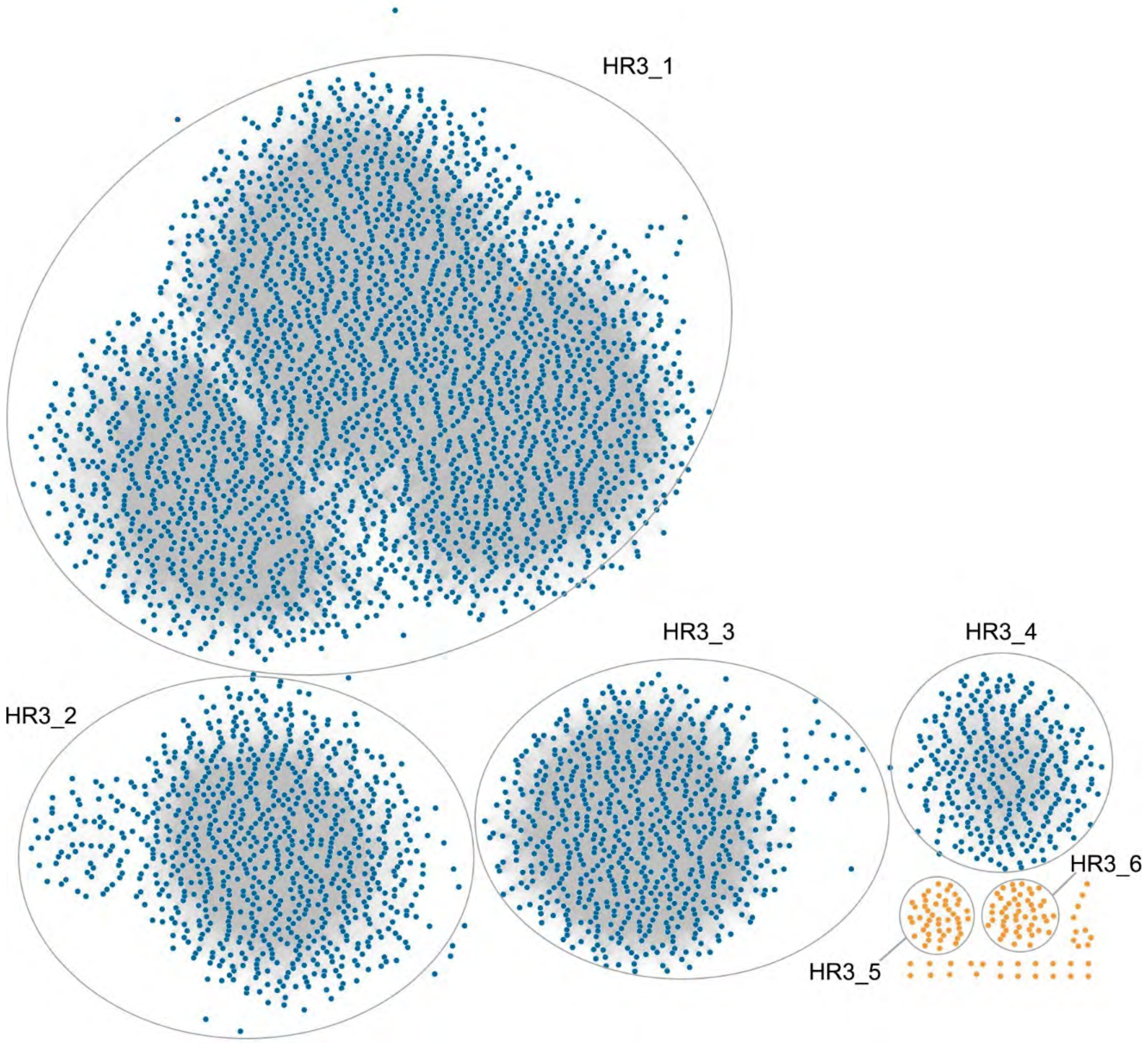

HR4

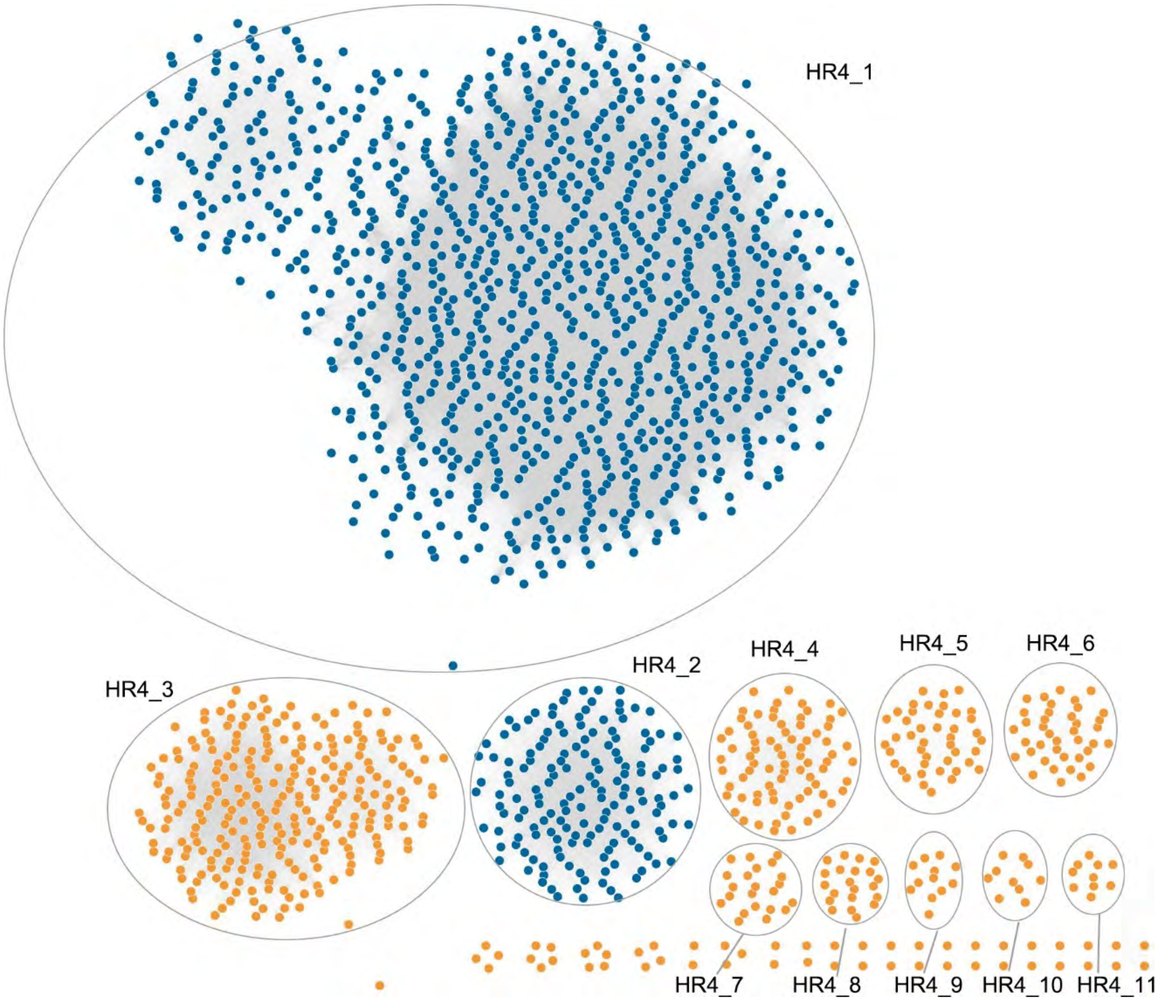

HR5

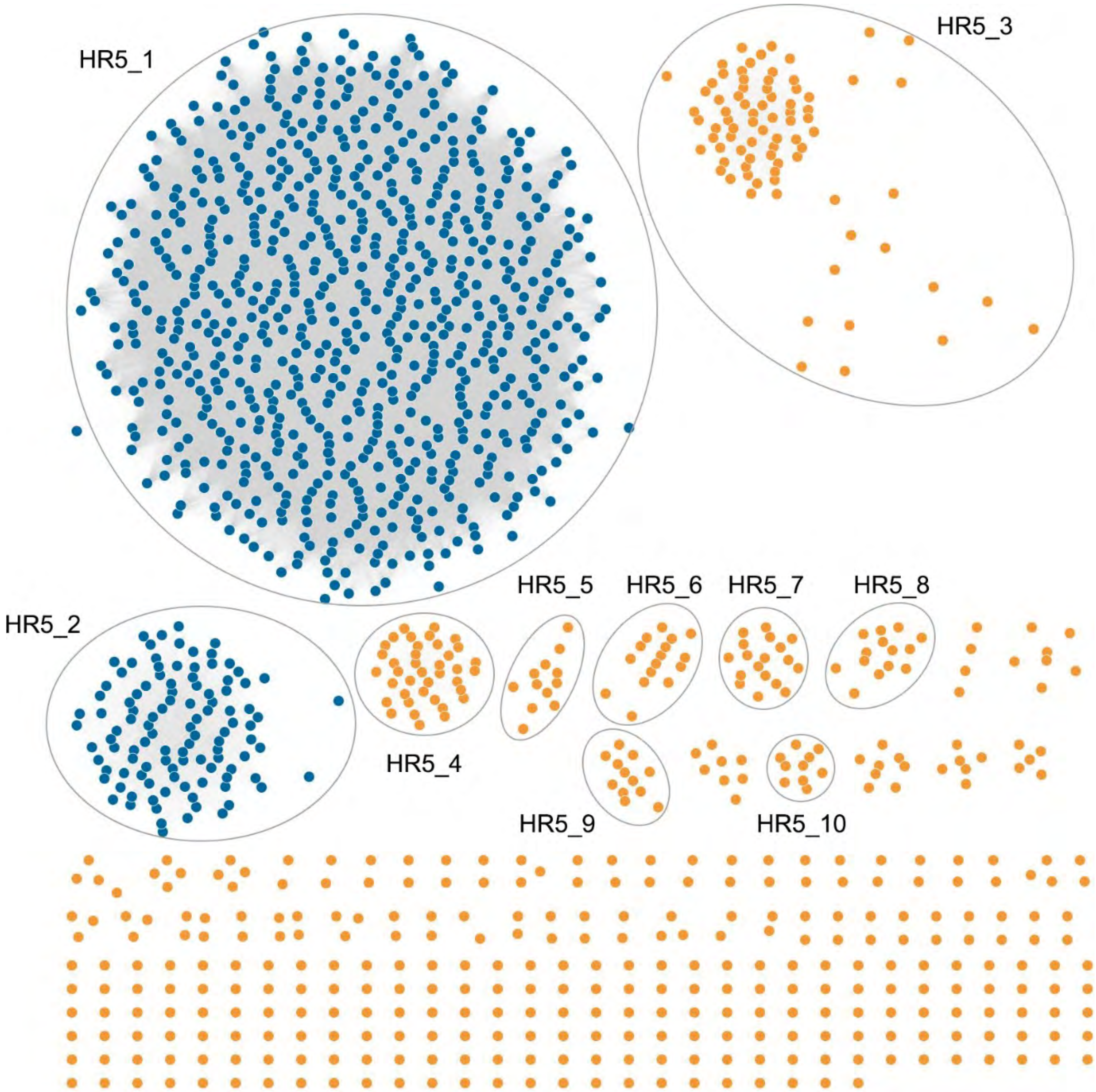

HR6

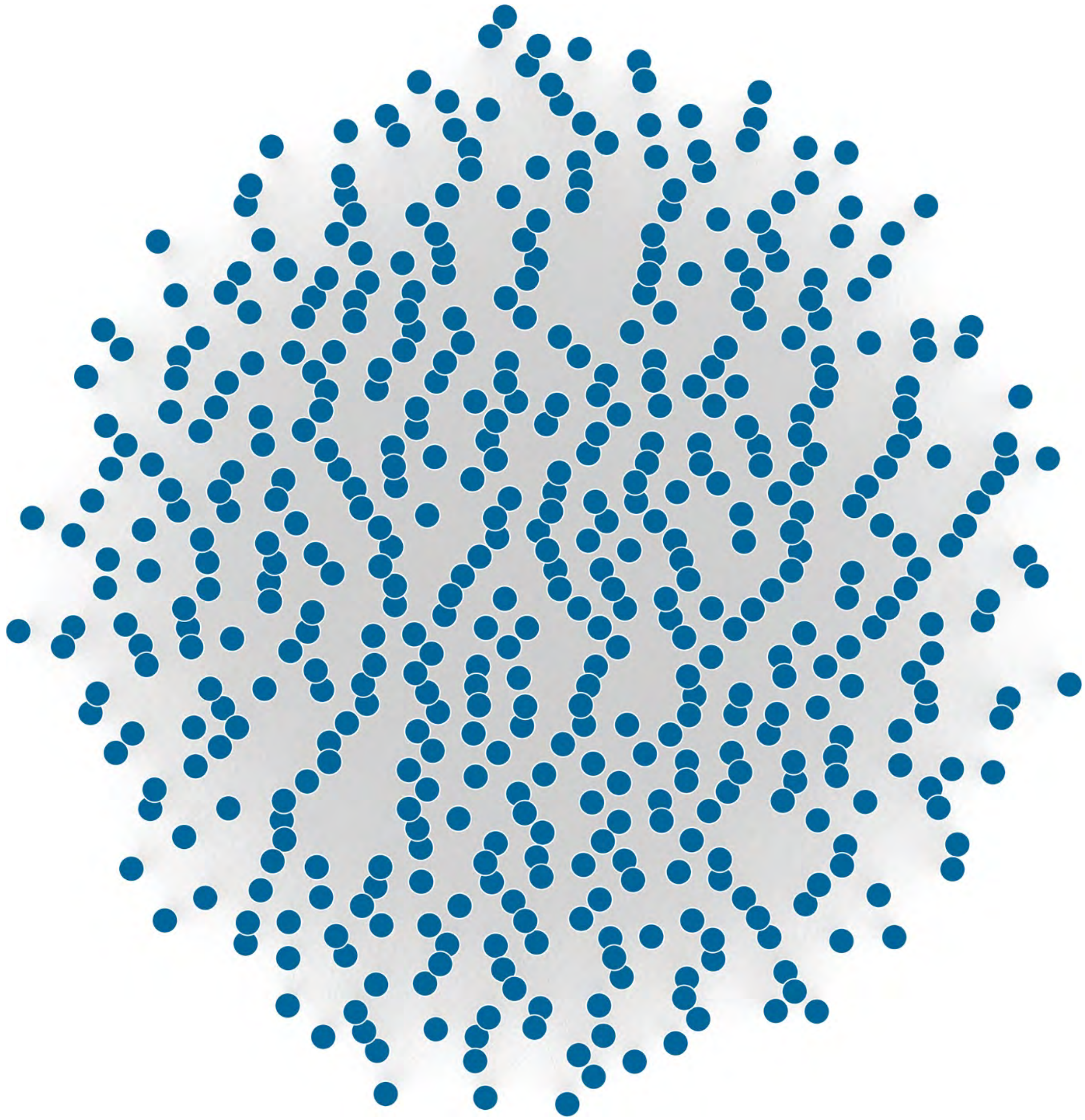

HR7

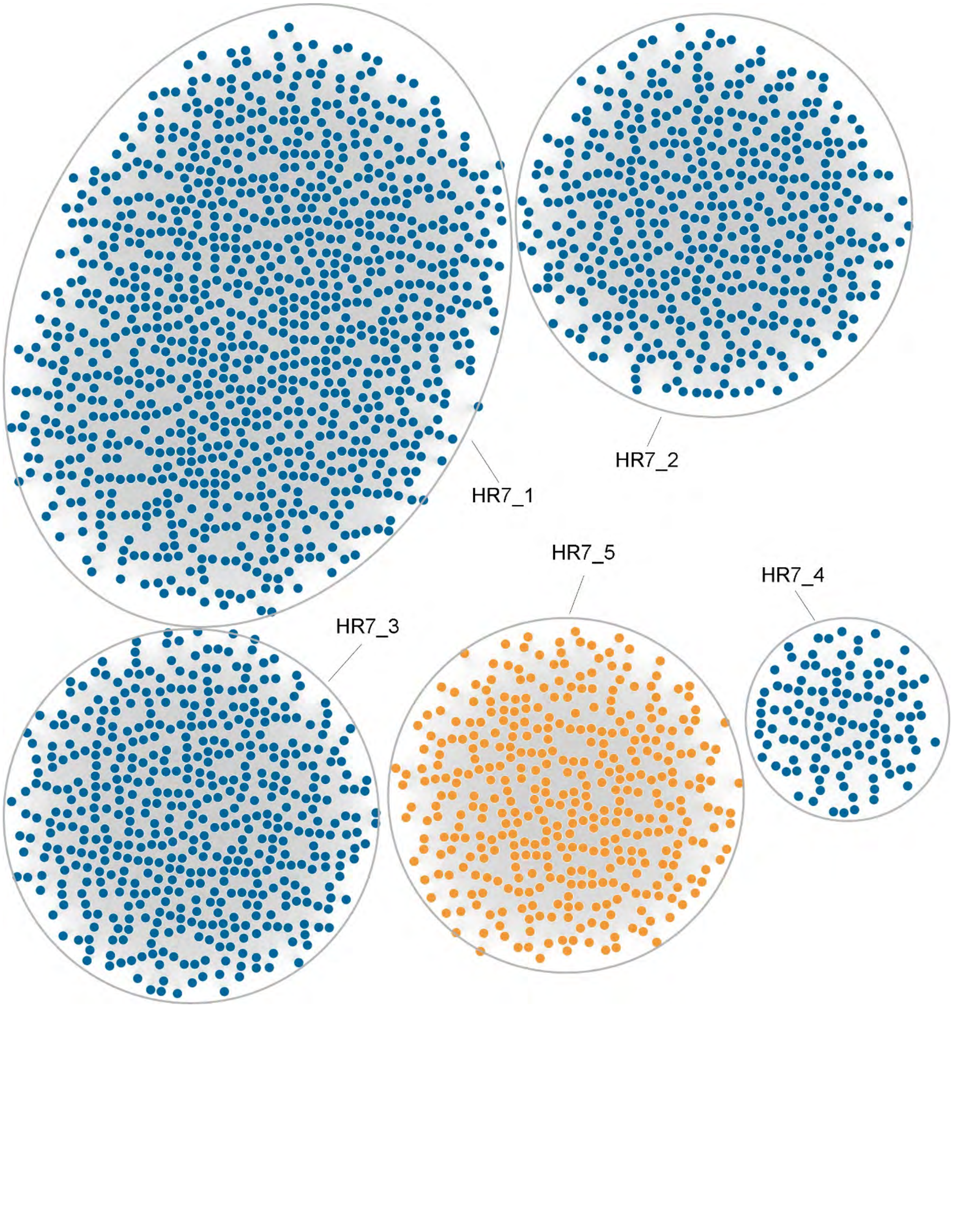

HR8

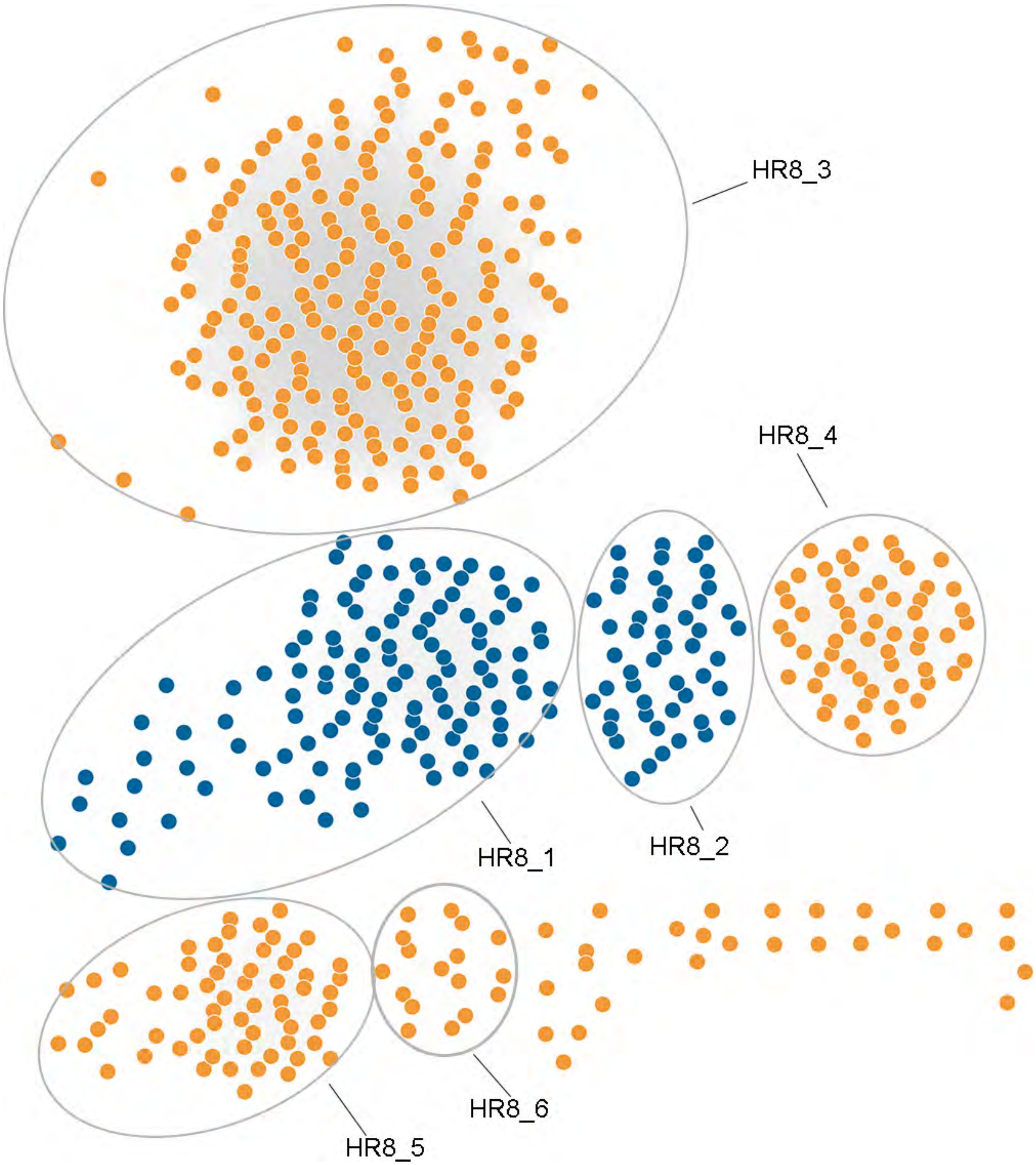

IR1

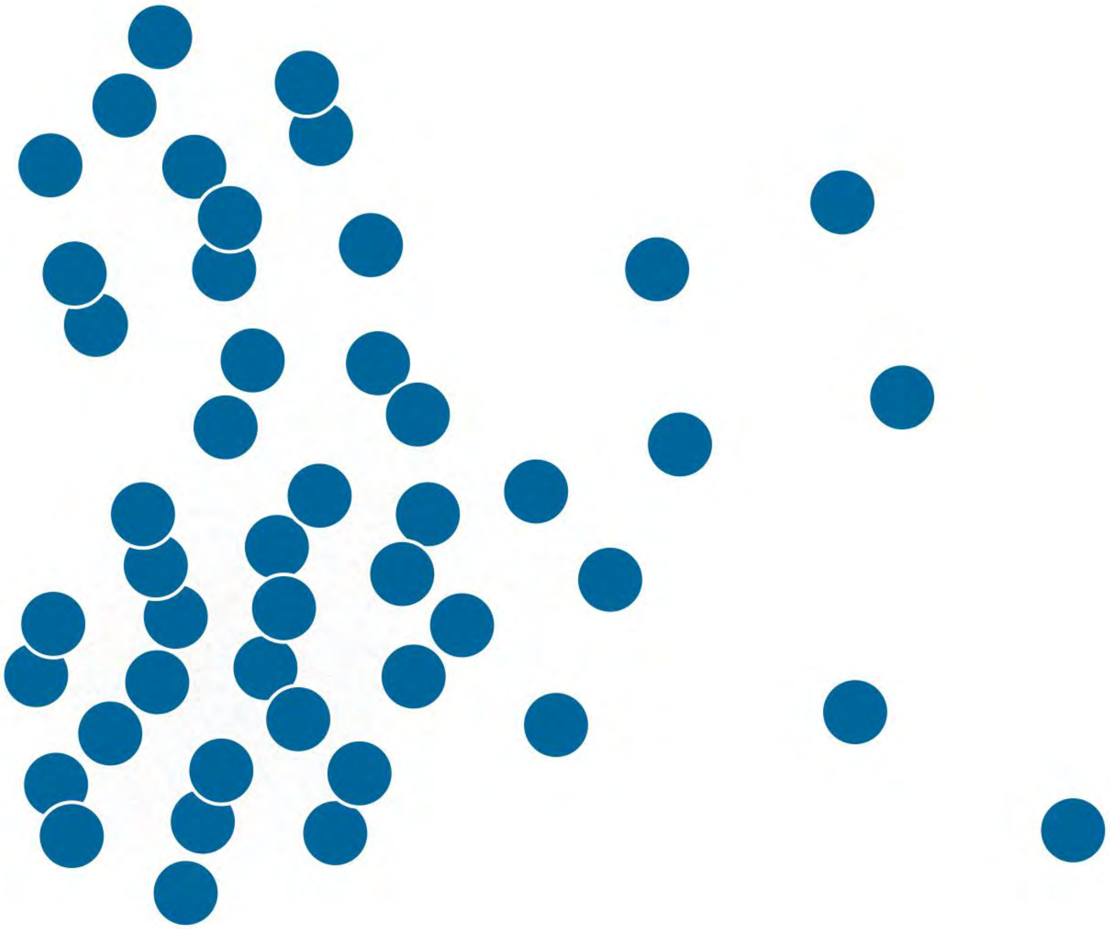

IR2

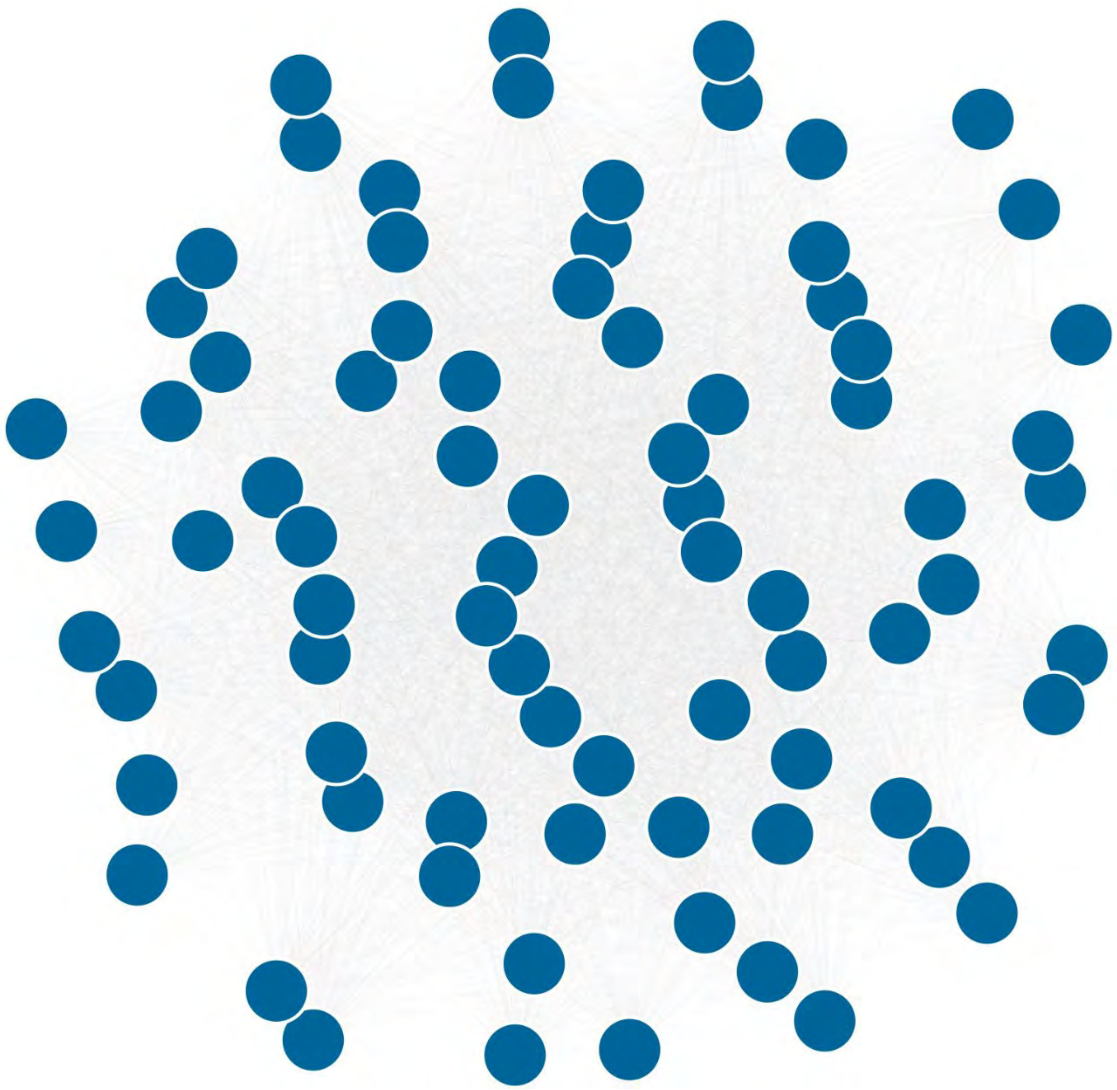

NCR1

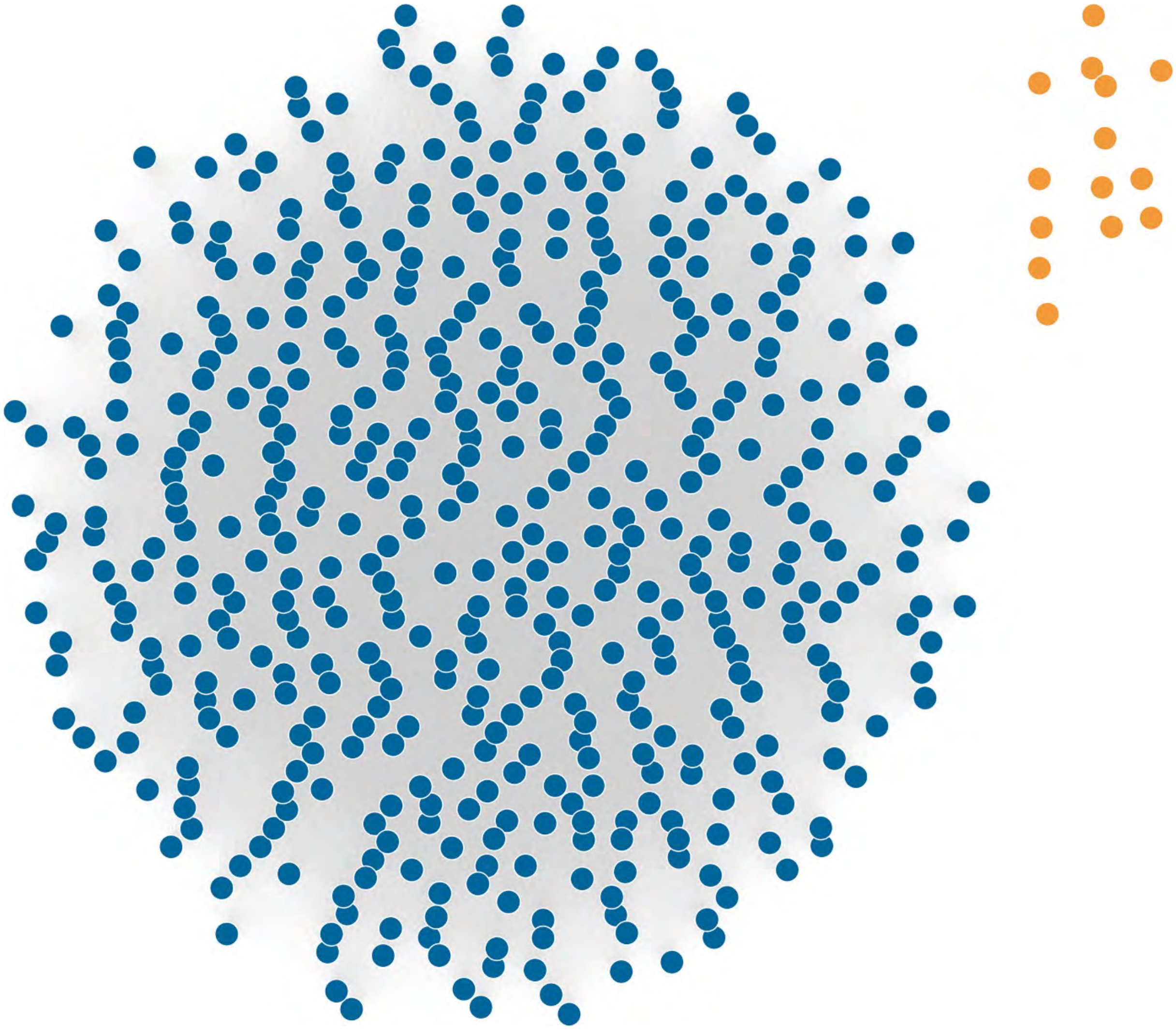

OR1

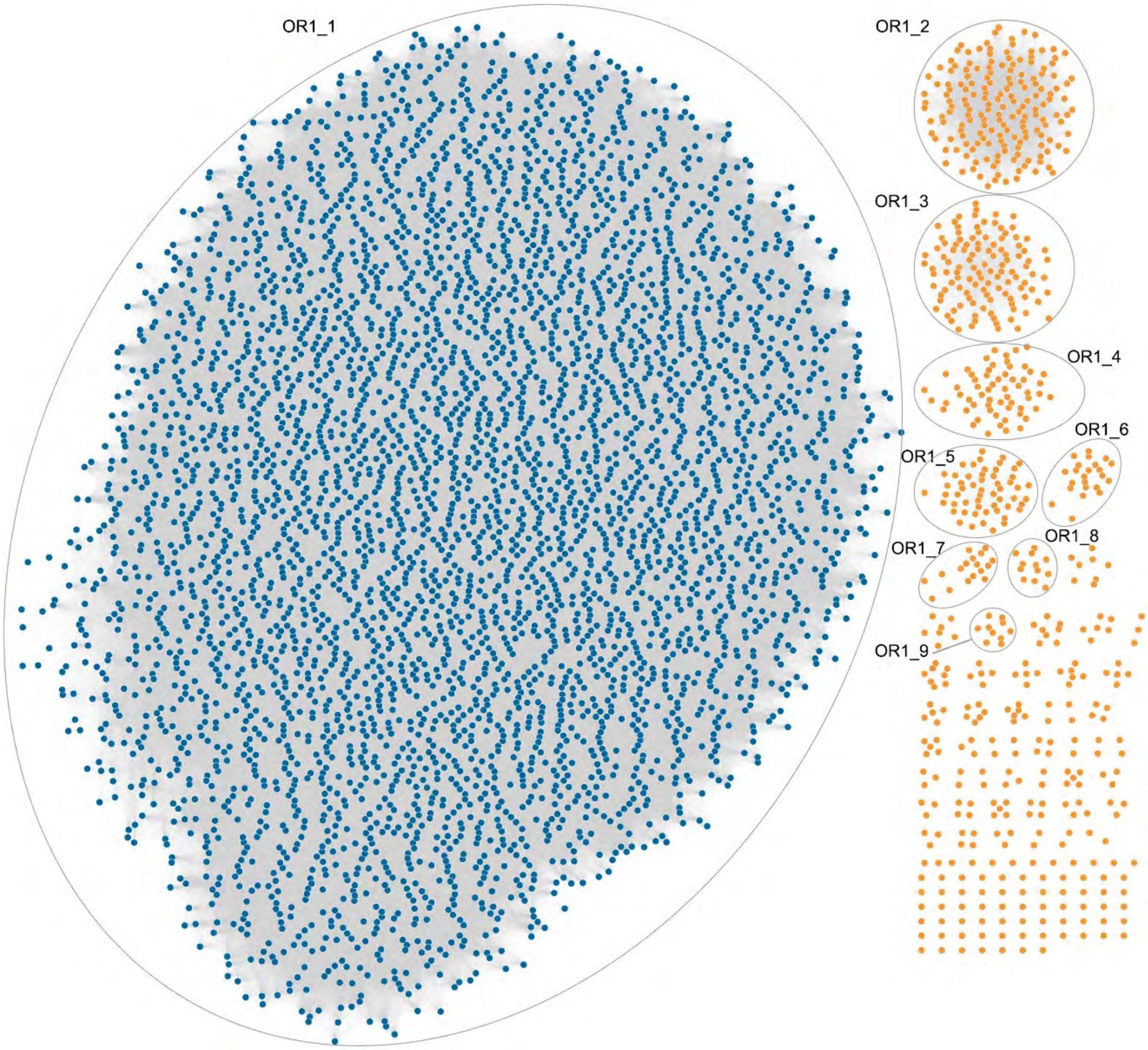

OR2

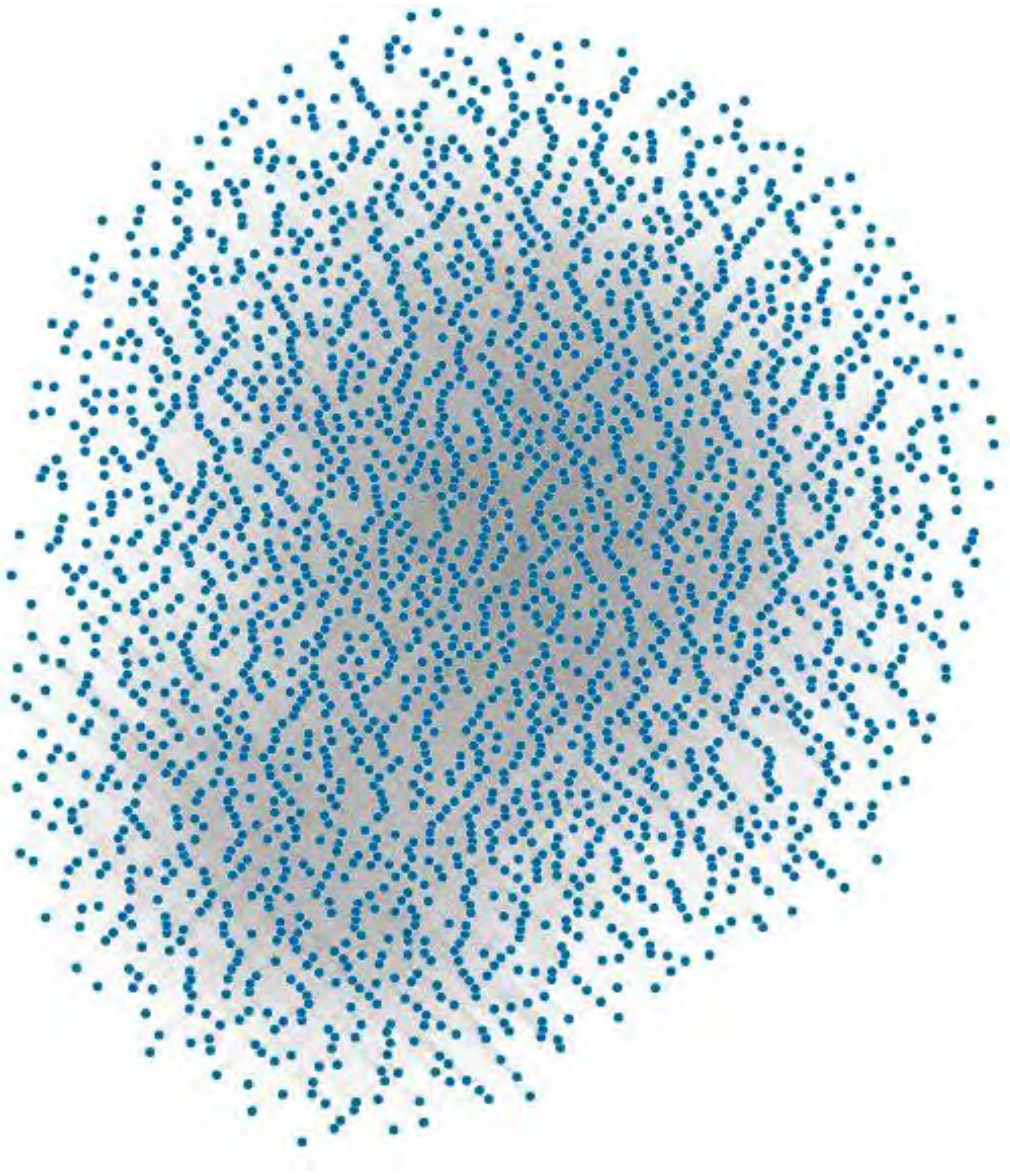

OR3

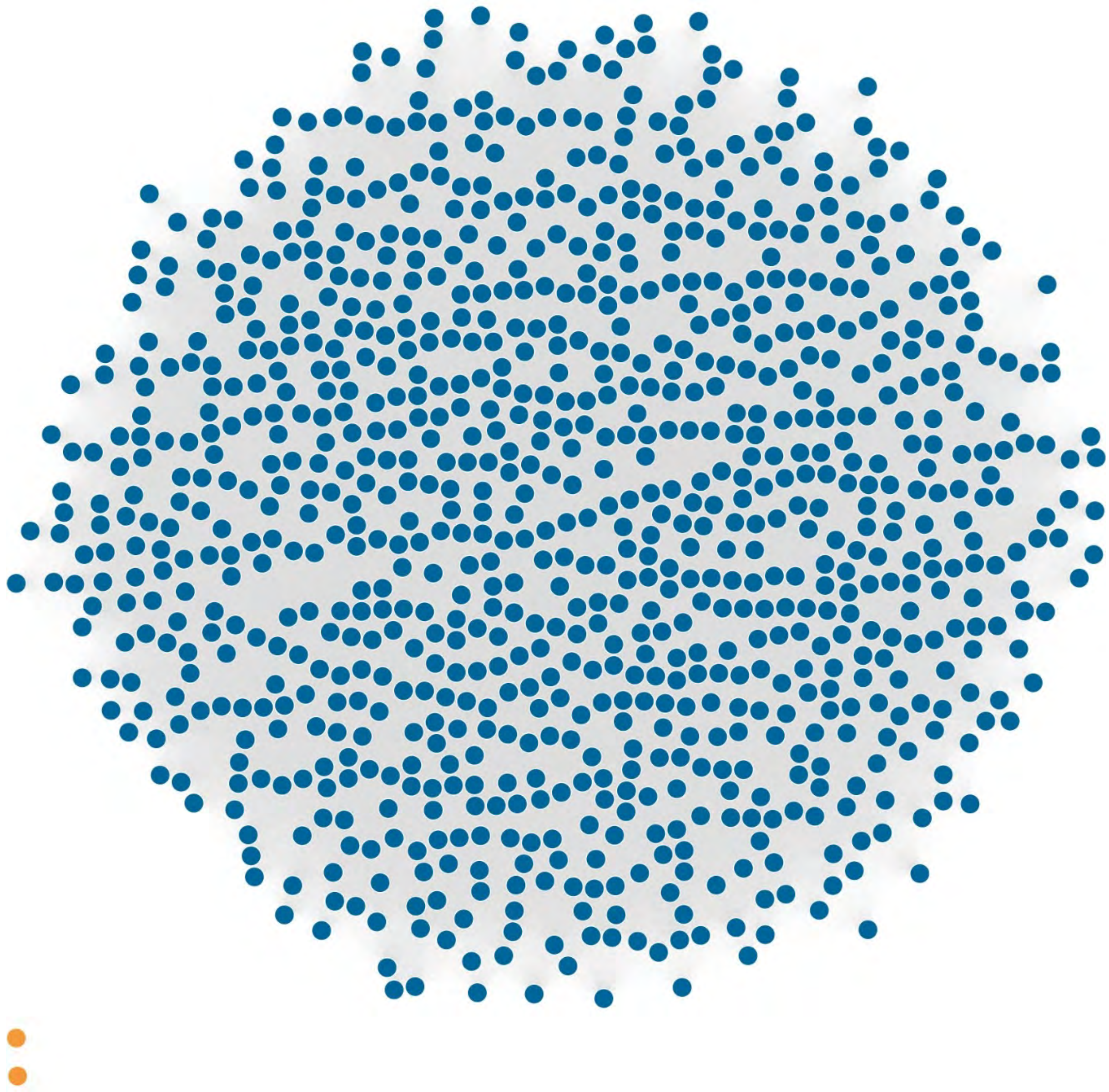

OR4

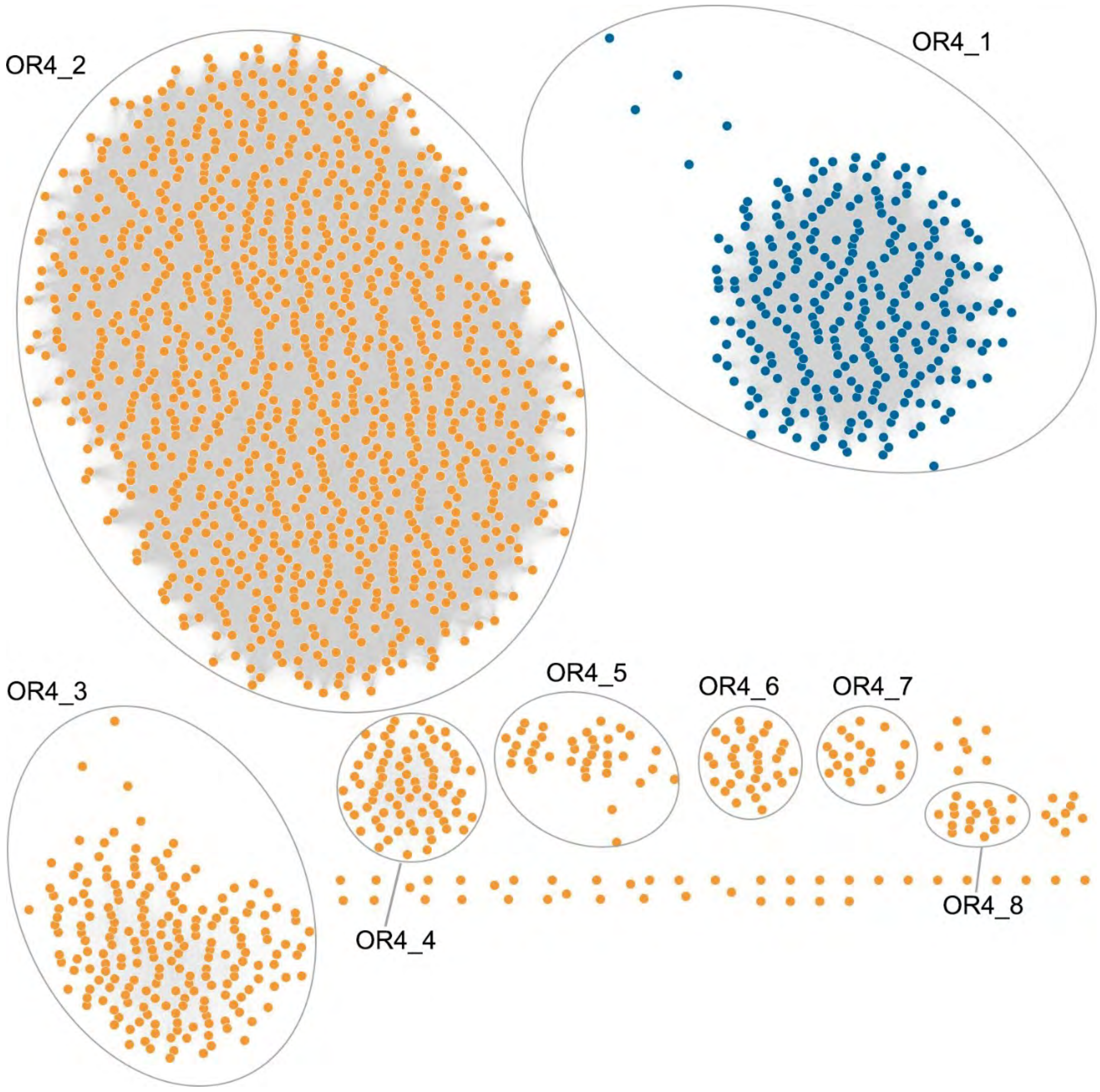

OR5

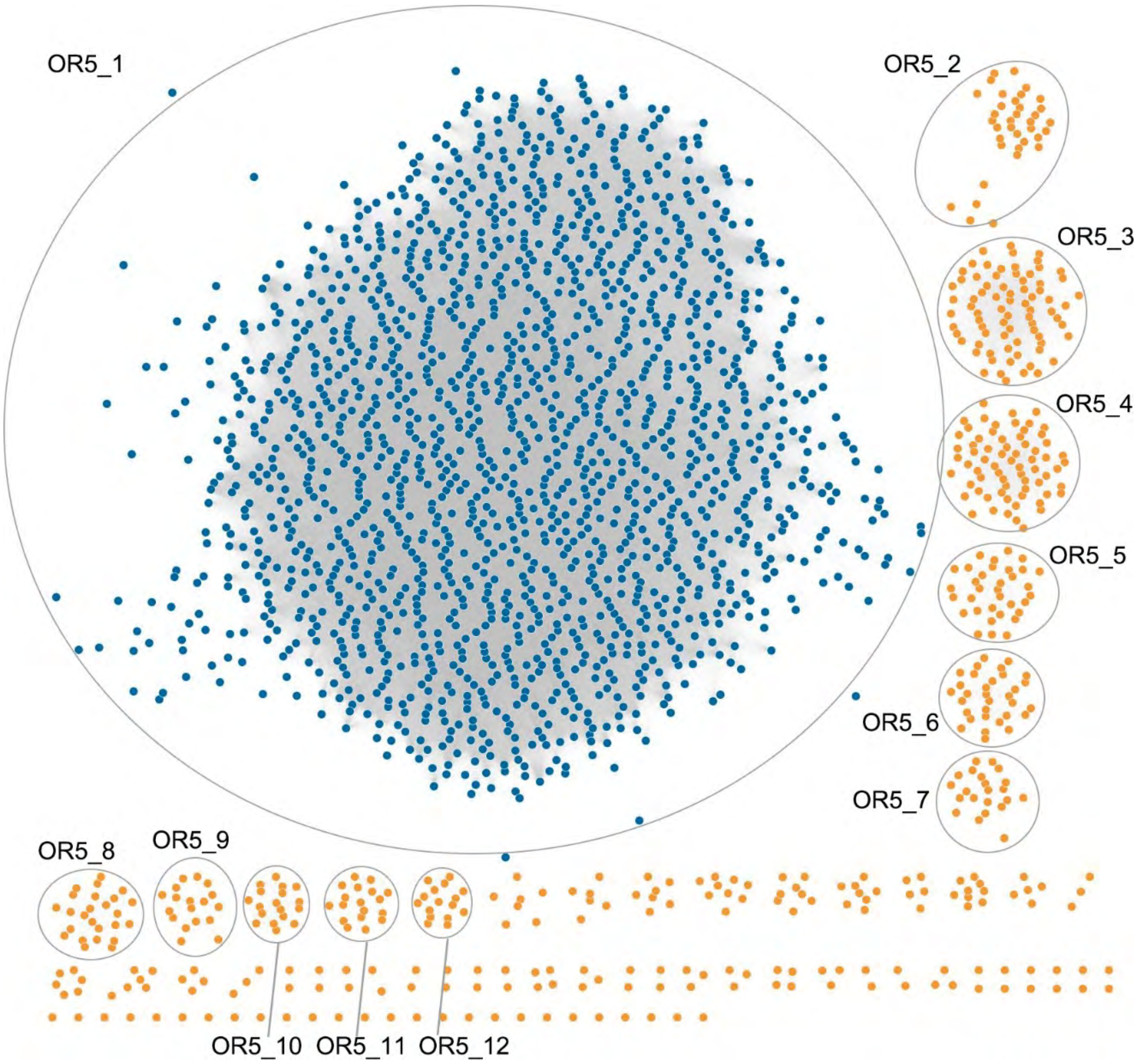

OR5_1
OR5_2
OR5_3
OR5_4
OR5_5
OR5_6
OR5_7
OR5_8
OR5_9
OR5_10
OR5_11
OR5_12

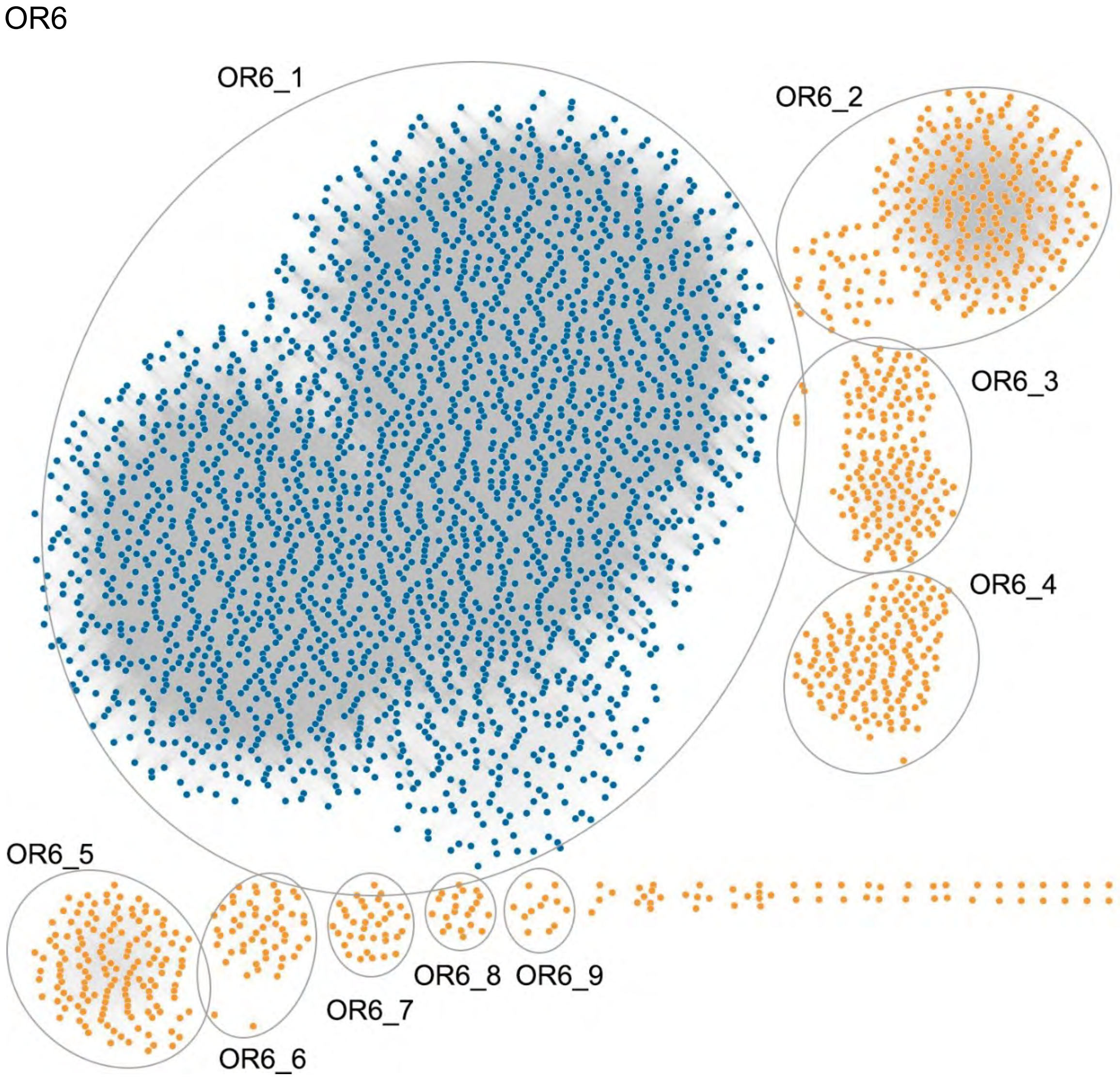
OR6
OR6_1
OR6_2
OR6_3
OR6_4
OR6_5
OR6_6
OR6_7
OR6_8
OR6_9

OR7

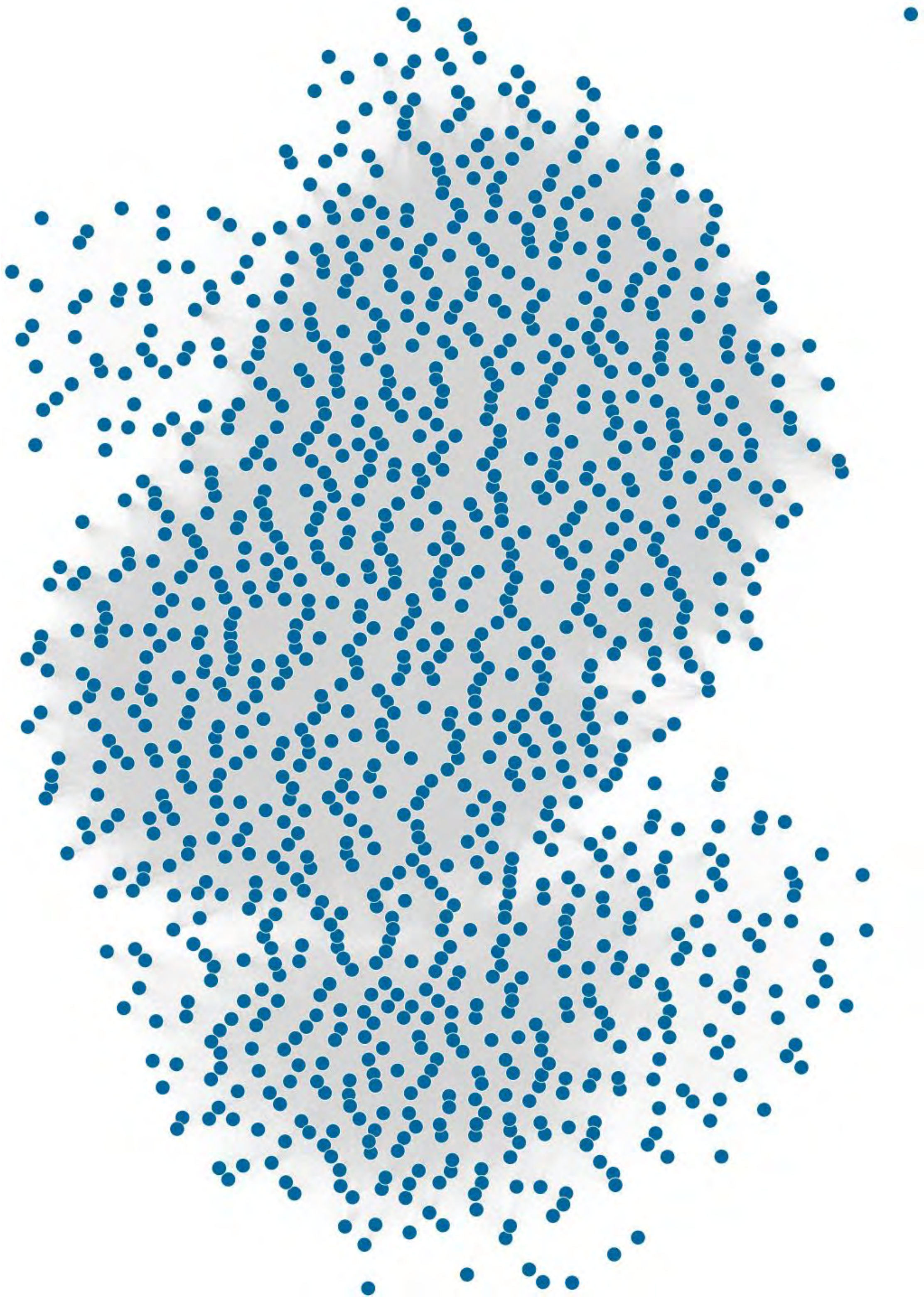

OR8

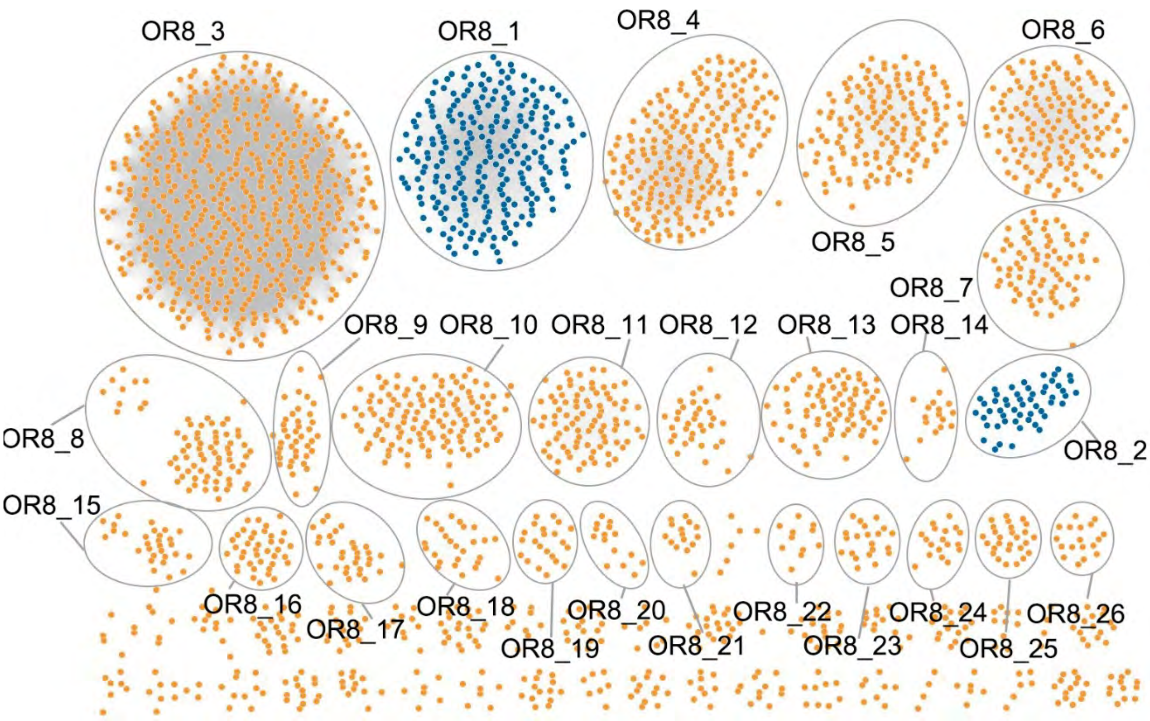
OR8_3
OR8_1
OR8_4
OR8_6
OR8_5
OR8_7
OR8_9
OR8_10
OR8_11
OR8_12
OR8_13
OR8_14
OR8_8
OR8_2
OR8_15
OR8_16
OR8_17
OR8_18
OR8_19
OR8_20
OR8_21
OR8_22
OR8_23
OR8_24
OR8_25
OR8_26

OR9

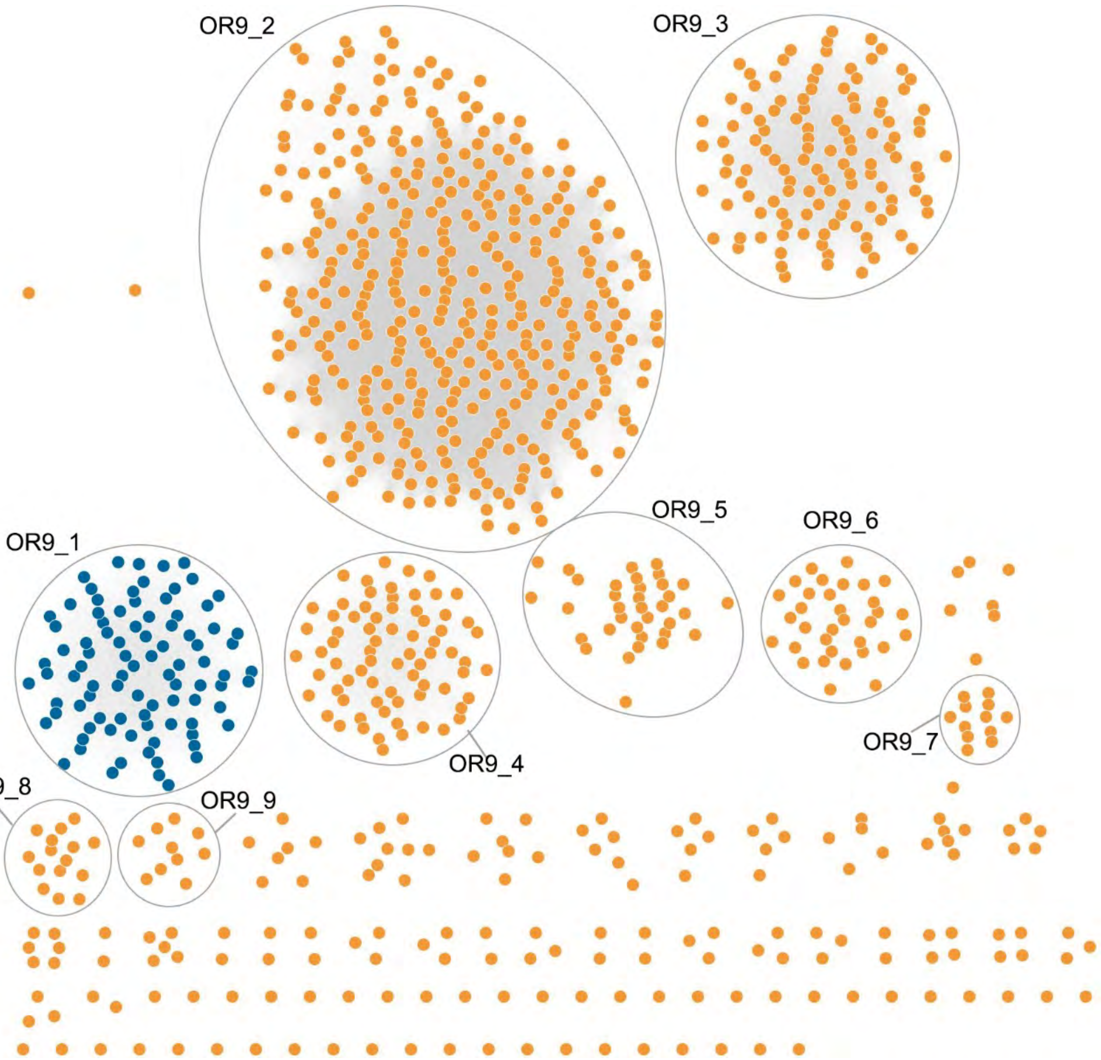

UC1

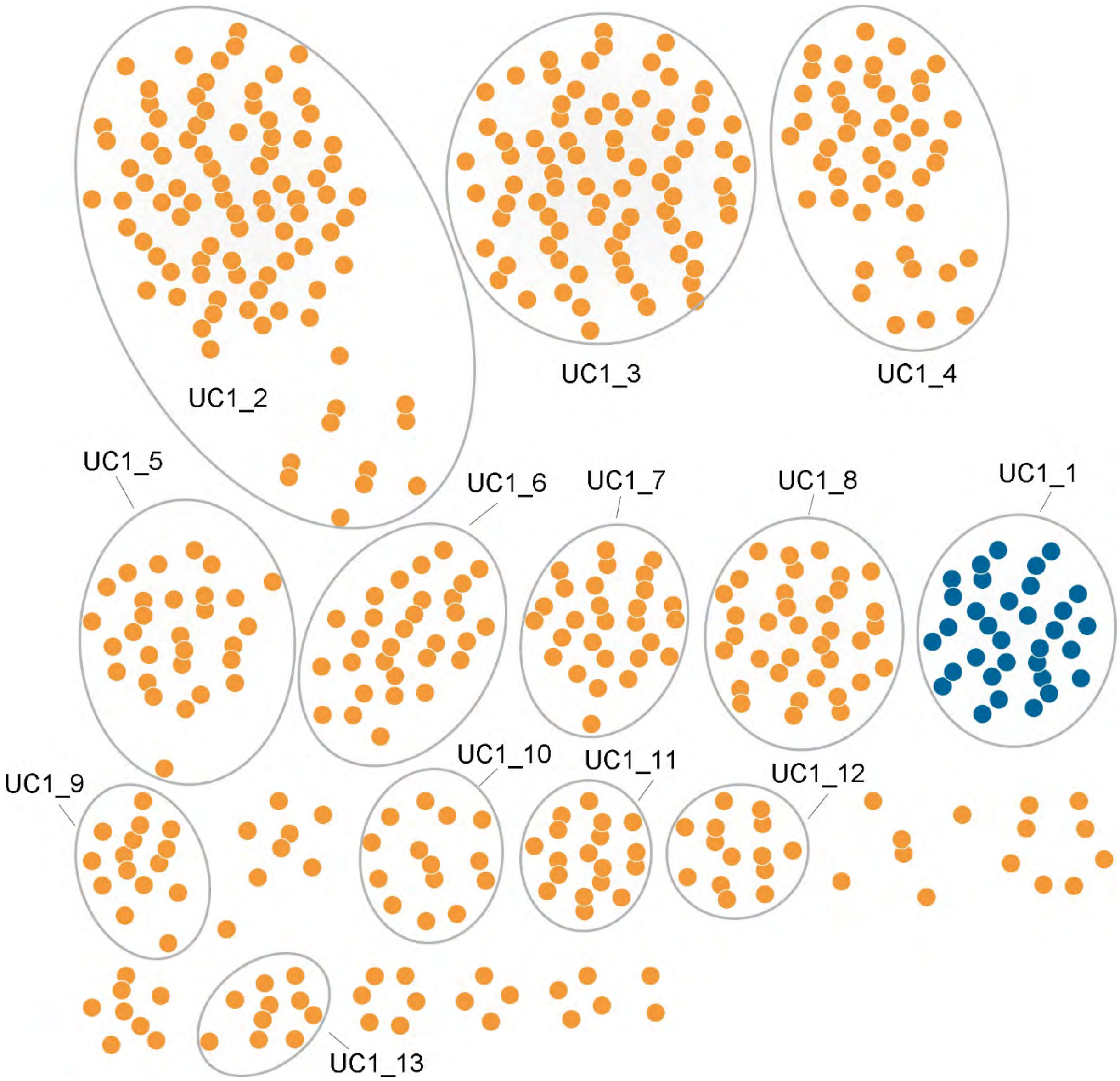

UC1_2
UC1_3
UC1_4
UC1_5
UC1_6
UC1_7
UC1_8
UC1_1
UC1_9
UC1_10
UC1_11
UC1_12
UC1_13

UC2

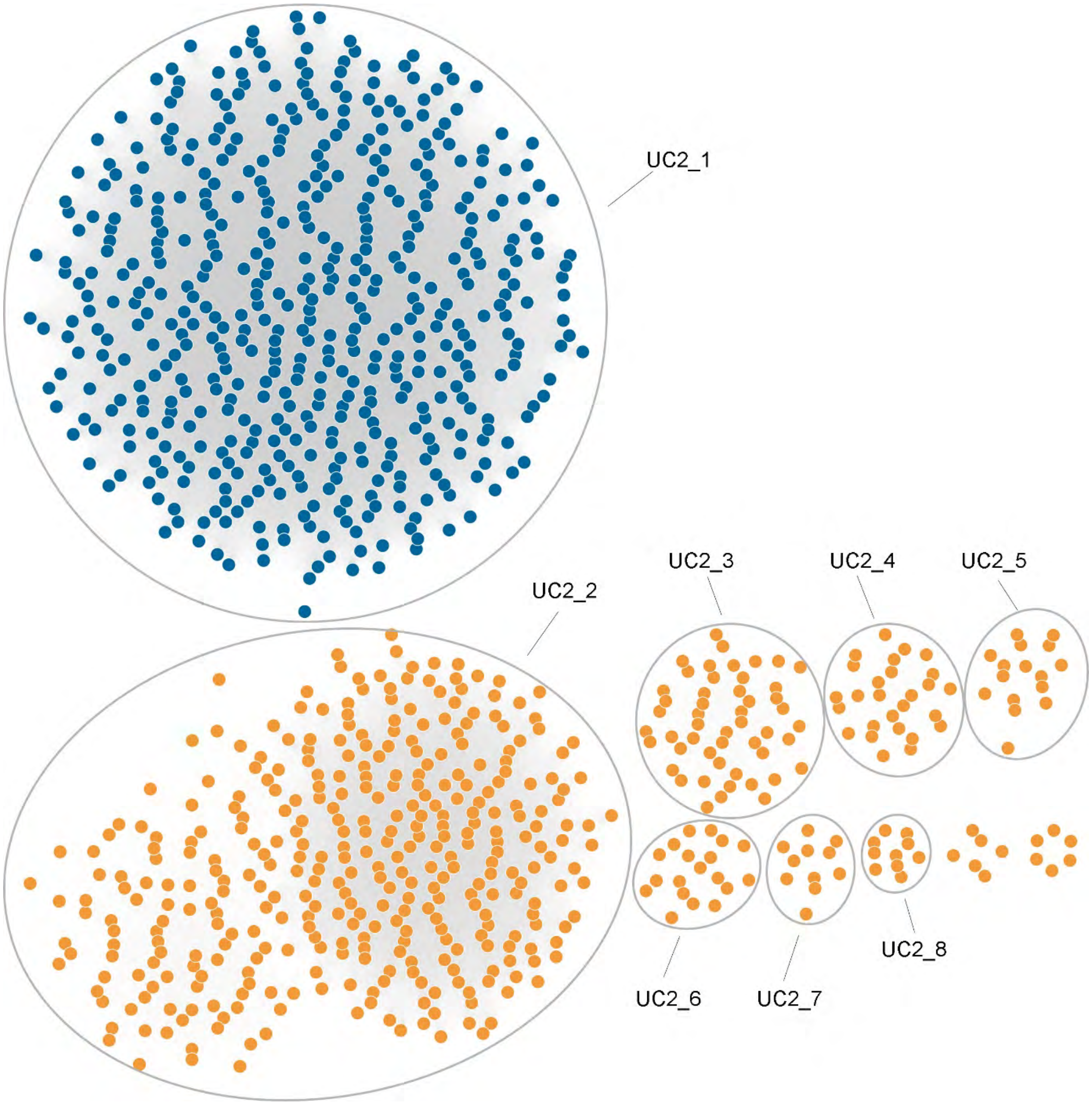

Data S3

Krona taxonomy distribution plots of PUP homologs in UniProt for each of the 26 PUP families

FR1

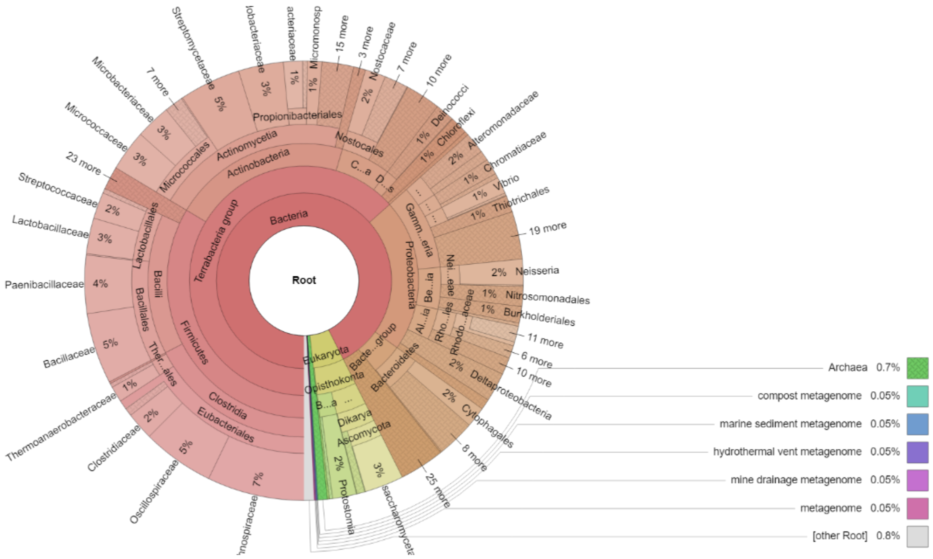

Root
Bacteria
Terrabacteria group
Actinobacteria
Actinomycetia
Propionibacteriales
Micrococcales
Firmicutes
Bacilli
Bacillales
Lactobacillales
Clostridia
Eubacteriales
Proteobacteria
Bacteroidetes
Eukaryota
Opisthokonta
Dikarya
Ascomycota
Streptomycetaceae 5%
Microbacteriaceae 3%
Micrococcaceae 3%
23 more
7 more
Streptococcaceae 2%
Lactobacillaceae 3%
Paenibacillaceae 4%
Bacillaceae 5%
Thermoanaerobacteraceae 1%
Clostridiaceae 2%
Oscillospiraceae 5%
Nostocales
Nostocaceae 2%
15 more
3 more
7 more
10 more
Deinococci 1%
Chloroflexi 1%
Alteromonadaceae 2%
Chromatiaceae 1%
Vibrio 1%
Thiotrichales 1%
19 more
Neisseria 2%
Nitrosomonadales 1%
Burkholderiales 1%
11 more
6 more
10 more
Deltaproteobacteria 2%
Cytophagales 2%
8 more
25 more
Protostomia 2%
Archaea 0.7%
compost metagenome 0.05%
marine sediment metagenome 0.05%
hydrothermal vent metagenome 0.05%
mine drainage metagenome 0.05%
metagenome 0.05%
[other Root] 0.8%

FR2

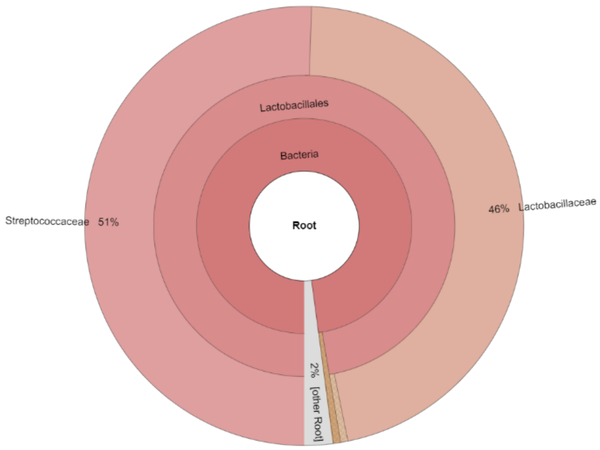

Lactobacillales
Bacteria
Root
Streptococcaceae 51%
46% Lactobacillaceae
2% [other Root]

FR3

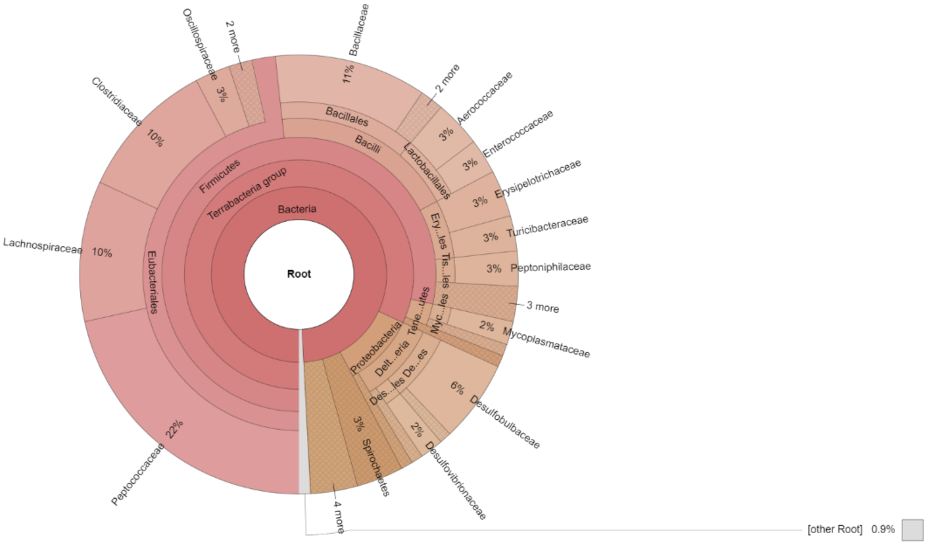
Root
Bacteria
Terrabacteria group
Firmicutes
Eubacteriales
Bacilli
Bacillales
Lactobacillales
Proteobacteria
Spirochaetes
Peptococcaceae 22%
Lachnospiraceae 10%
Clostridiaceae 10%
Oscillospiraceae 3%
2 more
Bacillaceae 11%
2 more
Aerococcaceae 3%
Enterococcaceae 3%
Erysipelotrichaceae 3%
Turicibacteraceae 3%
Peptoniphilaceae 3%
3 more
Mycoplasmataceae 2%
Desulfobulbaceae 6%
Desulfovibrionaceae 2%
3%
4 more
[other Root] 0.9%

FR4

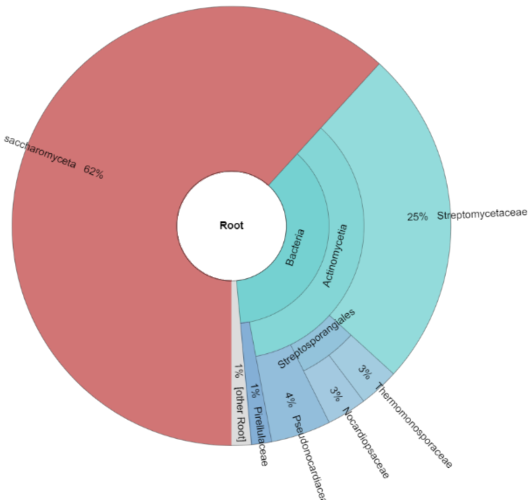

Root
saccharomyceta 62%
Bacteria
Actinomycetia
25% Streptomycetaceae
Streptosporangiales
3% Thermomonosporaceae
3% Nocardiopsaceae
4% Pseudonocardiaceae
1% Pirellulaceae
1% [other Root]

HR1

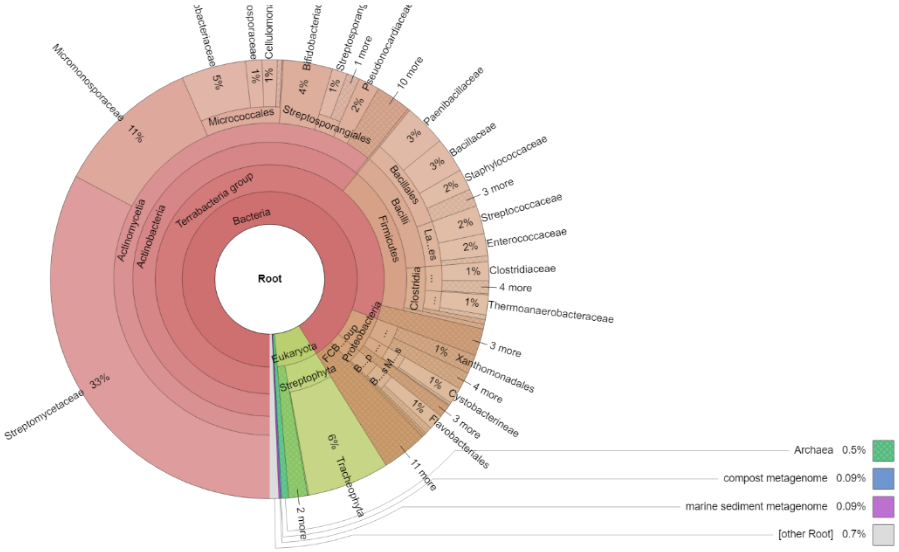

Root
Bacteria
Terrabacteria group
Actinobacteria
Actinomycetia
Micromonosporaceae
11%
Streptomycetaceae
33%
Micrococcales
5%
1%
1%
Cellulomon
Bifidobacteriac
4%
1%
Streptosporangiales
2%
1 more
Pseudonocardiaceae
10 more
Firmicutes
Bacilli
Bacillales
Paenibacillaceae
3%
Bacillaceae
3%
Staphylococcaceae
2%
3 more
La...es
Streptococcaceae
2%
Enterococcaceae
2%
Clostridia
Clostridiaceae
1%
4 more
Thermoanaerobacteraceae
1%
3 more
Proteobacteria
FCB...oup
Xanthomonadales
1%
4 more
Cystobacterineae
1%
3 more
Flavobacteriales
1%
11 more
Eukaryota
Streptophyta
Tracheophyta
6%
2 more
Archaea 0.5%
compost metagenome 0.09%
marine sediment metagenome 0.09%
[other Root] 0.7%

HR2

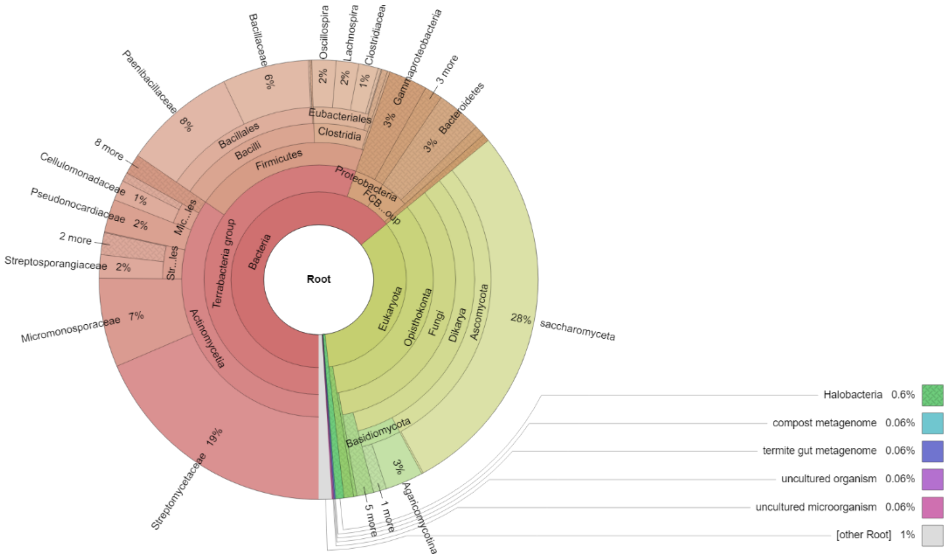

Root
Bacteria
Terrabacteria group
Actinomycetia
Firmicutes
Bacilli
Bacillales
Clostridia
Eubacteriales
Proteobacteria
Paenibacillaceae 8%
Bacillaceae 6%
Oscillospira 2%
Lachnospira 2%
Clostridiaceae 1%
Gammaproteobacteria 3%
3 more
Bacteroidetes 3%
8 more
Cellulomonadaceae 1%
Pseudonocardiaceae 2%
2 more
Streptosporangiaceae 2%
Micromonosporaceae 7%
Streptomycetaceae 19%
Eukaryota
Opisthokonta
Fungi
Dikarya
Ascomycota
saccharomyceta 28%
Basidiomycota
Agaricomycotina 3%
1 more
5 more
Halobacteria 0.6%
compost metagenome 0.06%
termite gut metagenome 0.06%
uncultured organism 0.06%
uncultured microorganism 0.06%
[other Root] 1%

HR3

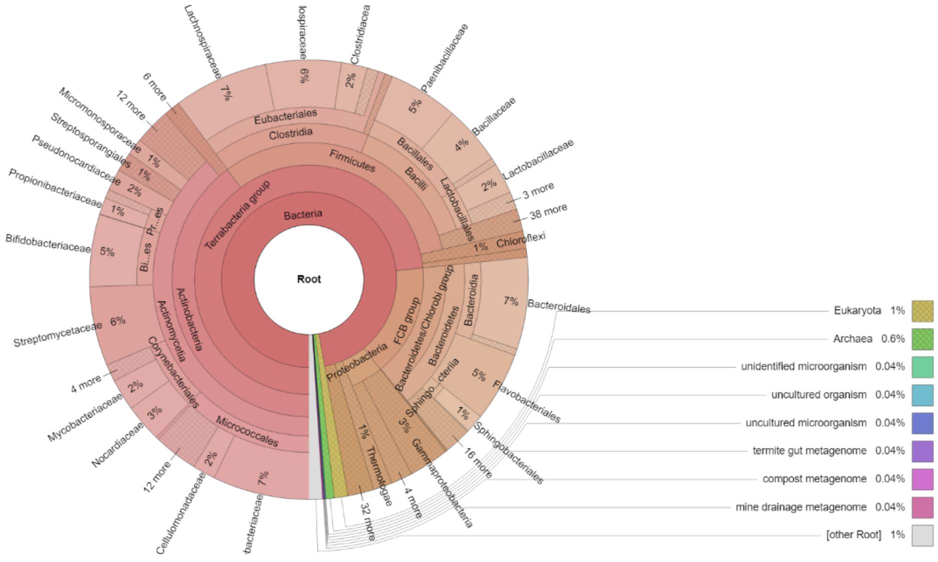

Root
Bacteria
Terrabacteria group
Actinobacteria
Actinomycetia
Firmicutes
Clostridia
Eubacteriales
Bacilli
Bacillales
Lactobacillales
Chloroflexi
FCB group
Bacteroidetes/Chlorobi group
Bacteroidetes
Bacteroidia
Bacteroidales
Flavobacteriales
Sphingobacteriales
Proteobacteria
Gammaproteobacteria
Thermotogae
Corynebacteriales
Micrococcales
Lachnospiraceae
Clostridiaceae
Paenibacillaceae
Bacillaceae
Lactobacillaceae
Micromonosporaceae
Streptosporangiales
Pseudonocardiaceae
Propionibacteriaceae
Bifidobacteriaceae
Streptomycetaceae
Mycobacteriaceae
Nocardiaceae
Cellulomonadaceae
Eukaryota 1%
Archaea 0.6%
unidentified microorganism 0.04%
uncultured organism 0.04%
uncultured microorganism 0.04%
termite gut metagenome 0.04%
compost metagenome 0.04%
mine drainage metagenome 0.04%
[other Root] 1%

HR4

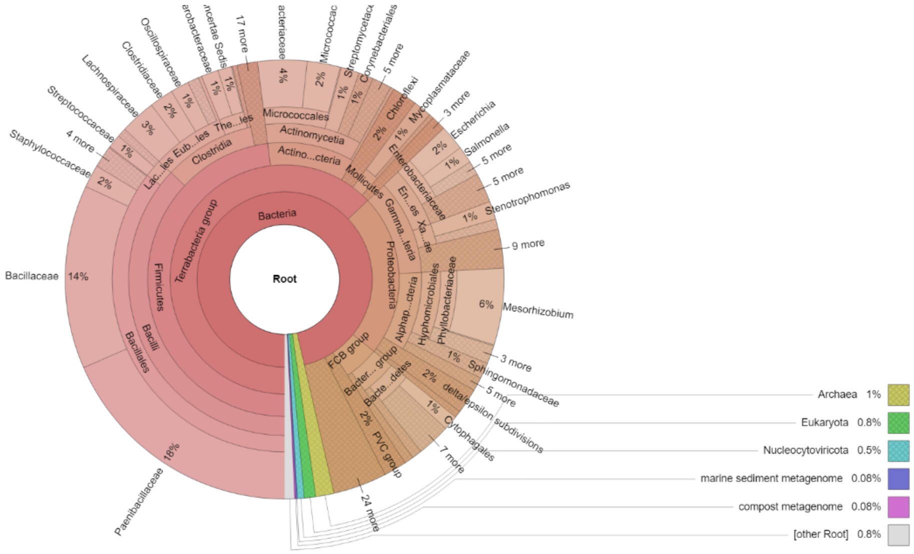
Root
Bacteria
Terrabacteria group
Firmicutes
Bacilli
Bacillales
Bacillaceae 14%
Paenibacillaceae 18%
Staphylococcaceae 2%
4 more
Streptococcaceae 1%
Lachnospiraceae 3%
Clostridiaceae 2%
Oscillospiraceae 1%
Clostridia
17 more
Micrococcales
Actinomycetia
Actino...cteria
Micrococcac 2%
Streptomycetac 1%
Corynebacteriales 1%
5 more
Chloroflexi 2%
Mycoplasmataceae 1%
Mollicutes
3 more
Escherichia 2%
Salmonella 1%
5 more
5 more
Stenotrophomonas 1%
Enterobacteriaceae
Proteobacteria
9 more
Mesorhizobium 6%
Phyllobacteriaceae
Hyphomicrobiales
Alphap...cteria
3 more
Sphingomonadaceae 1%
5 more
delta/epsilon subdivisions 2%
Cytophagales 1%
7 more
FCB group
PVC group 2%
24 more
Archaea 1%
Eukaryota 0.8%
Nucleocytoviricota 0.5%
marine sediment metagenome 0.08%
compost metagenome 0.08%
[other Root] 0.8%

HR5

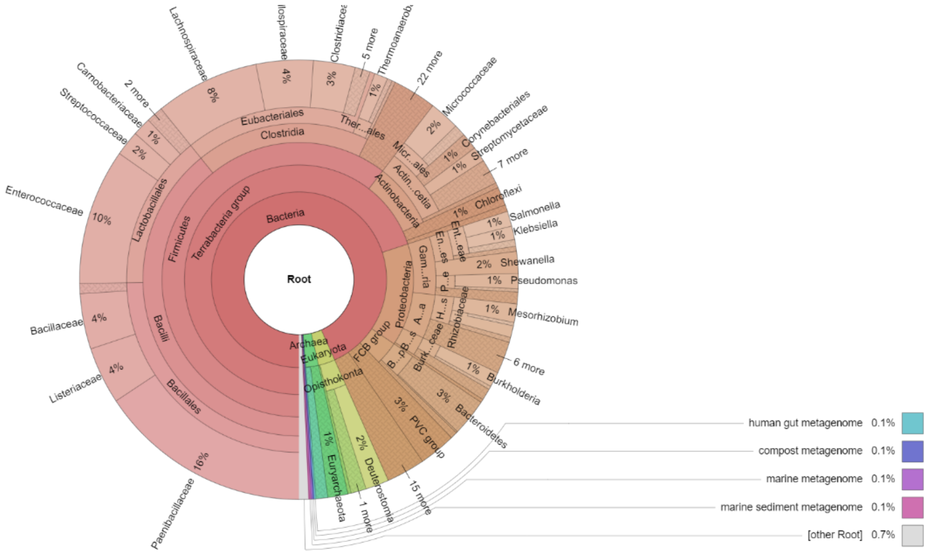

Root
Bacteria
Terrabacteria group
Firmicutes
Bacilli
Bacillales
Lactobacillales
Clostridia
Eubacteriales
Lachnospiraceae
8%
4%
Clostridiaceae
3%
5 more
Thermoanaerob
1%
Ther...ales
22 more
Micrococcaceae
2%
Corynebacteriales
Streptomycetaceae
1%
1%
7 more
Micr...ales
Actin...cetia
Actinobacteria
Chloroflexi
1%
Salmonella
1%
Klebsiella
1%
Shewanella
2%
Pseudomonas
1%
Mesorhizobium
1%
6 more
Burkholderia
1%
Bacteroidetes
3%
PVC group
3%
15 more
FCB group
Proteobacteria
Gam...ria
Rhizobiaceae
Burk...ceae
Archaea
Euryarchaeota
1%
1 more
Eukaryota
Opisthokonta
Deuterostomia
2%
Paenibacillaceae
16%
Listeriaceae
4%
Bacillaceae
4%
Enterococcaceae
10%
Streptococcaceae
2%
Carnobacteriaceae
1%
2 more
human gut metagenome 0.1%
compost metagenome 0.1%
marine metagenome 0.1%
marine sediment metagenome 0.1%
[other Root] 0.7%

HR6

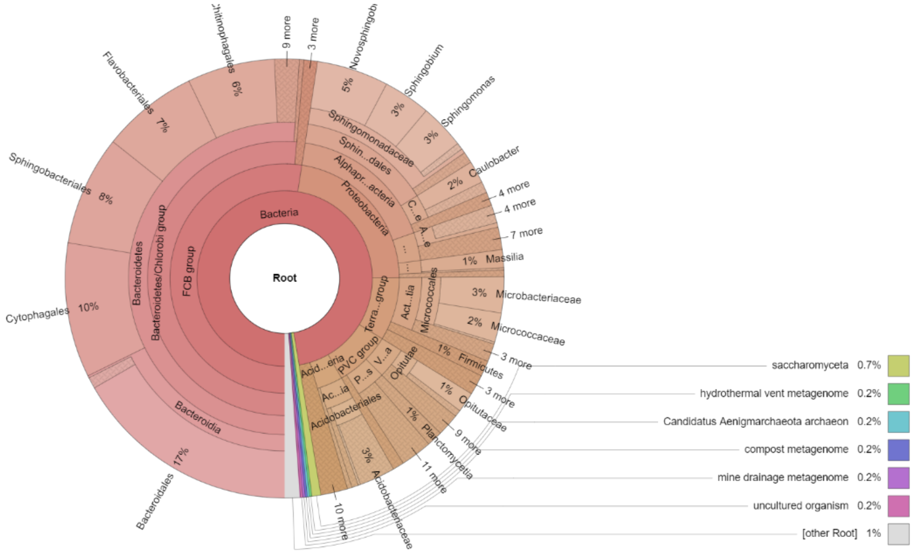

Root
Bacteria
FCB group
Bacteroidetes/Chlorobi group
Bacteroidetes
Bacteroidia
Bacteroidales 17%
Cytophagales 10%
Sphingobacteriales 8%
Flavobacteriales 7%
Chitinophagales 6%
9 more
3 more
Proteobacteria
Alphapr...acteria
Sphin...dales
Sphingomonadaceae
Novosphingobi
5%
Sphingobium 3%
Sphingomonas 3%
Caulobacter 2%
4 more
4 more
7 more
Massilia 1%
Terra...group
Act...tia
Micrococcales
Microbacteriaceae 3%
Micrococcaceae 2%
Firmicutes 1%
3 more
PVC group
V...a
P...s
Opitutae
Opitutaceae 1%
3 more
Planctomycetia 1%
9 more
11 more
Acid...eria
Ac...ia
Acidobacteriales
Acidobacteriaceae 3%
10 more
saccharomyceta 0.7%
hydrothermal vent metagenome 0.2%
Candidatus Aenigmarchaeota archaeon 0.2%
compost metagenome 0.2%
mine drainage metagenome 0.2%
uncultured organism 0.2%
[other Root] 1%

HR7

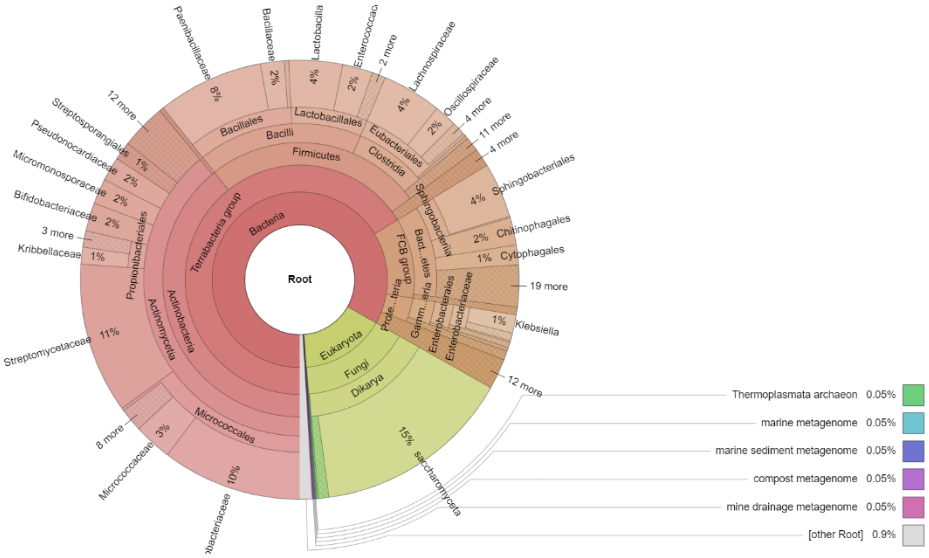

Root
Bacteria
Terrabacteria group
Firmicutes
Bacilli
Bacillales
Lactobacillales
Eubacteriales
Clostridia
Paenibacillaceae
8%
Bacillaceae
2%
Lactobacilla
4%
Enterococcac
2%
2 more
Lachnospiraceae
4%
Oscillospiraceae
2%
4 more
11 more
4 more
Sphingobacteriales
4%
Chitinophagales
2%
Cytophagales
1%
19 more
Klebsiella
1%
12 more
FCB group
Bact...etes
Sphingobacteriia
Prote...teria
Gamm...eria
Enterobacterales
Enterobacteriaceae
Eukaryota
Fungi
Dikarya
15%
saccharomyceta
Actinobacteria
Actinomycetia
Micrococcales
Propionibacteriales
12 more
Streptosporangiales
1%
Pseudonocardiaceae
2%
Micromonosporaceae
2%
Bifidobacteriaceae
2%
3 more
Kribbellaceae
1%
Streptomycetaceae
11%
8 more
Micrococcaceae
3%
10%
Thermoplasmata archaeon 0.05%
marine metagenome 0.05%
marine sediment metagenome 0.05%
compost metagenome 0.05%
mine drainage metagenome 0.05%
[other Root] 0.9%

HR8

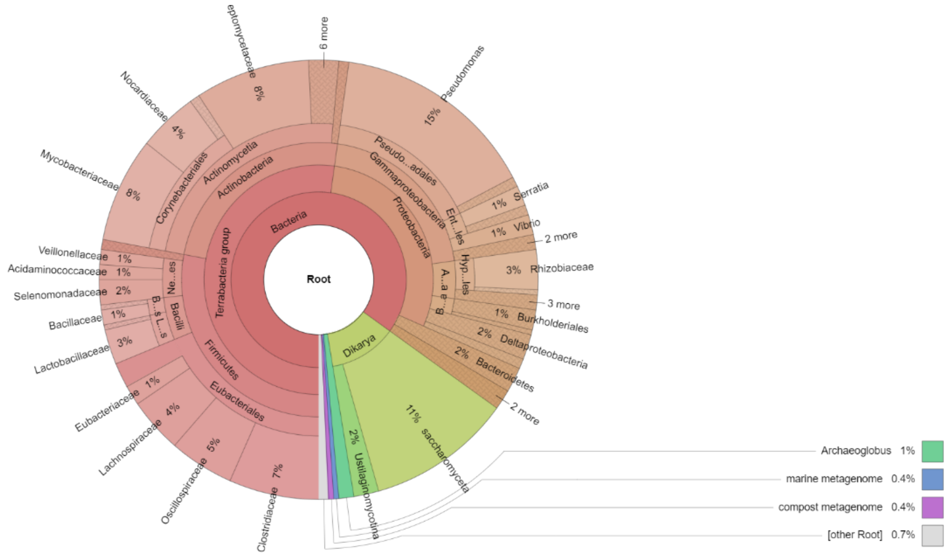
Root
Bacteria
Terrabacteria group
Actinobacteria
Actinomycetia
Corynebacteriales
Mycobacteriaceae 8%
Nocardiaceae 4%
Streptomycetaceae 8%
6 more
Pseudomonas 15%
Proteobacteria
Gammaproteobacteria
Pseudo...adales
Ent...les
Serratia 1%
Vibrio 1%
2 more
Hyp...les
A...a
B...
Rhizobiaceae 3%
3 more
Burkholderiales 1%
Deltaproteobacteria 2%
Bacteroidetes 2%
2 more
Dikarya
saccharomyceta 11%
Ustilaginomycotina 2%
Firmicutes
Eubacteriales
Clostridiaceae 7%
Oscillospiraceae 5%
Lachnospiraceae 4%
Eubacteriaceae 1%
Bacilli
Lactobacillaceae 3%
Bacillaceae 1%
Selenomonadaceae 2%
Acidaminococcaceae 1%
Veillonellaceae 1%
Ne...es
Archaeoglobus 1%
marine metagenome 0.4%
compost metagenome 0.4%
[other Root] 0.7%

IR1

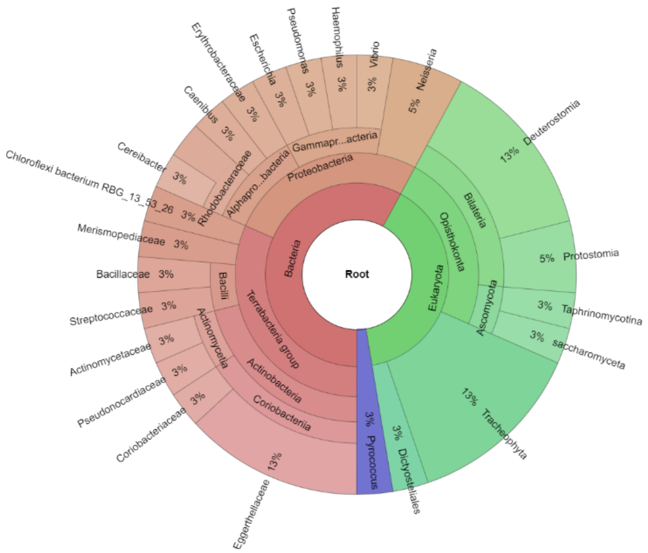
Root
Bacteria
Eukaryota
Proteobacteria
Terrabacteria group
Opisthokonta
Bilateria
Ascomycota
Gammapr...acteria
Alphapro...bacteria
Rhodobacteraceae
Actinobacteria
Coriobacteria
Actinomycetia
Bacilli
Erythrobacteraceae 3%
Escherichia 3%
Pseudomonas 3%
Haemophilus 3%
Vibrio 3%
Neisseria 5%
Deuterostomia 13%
Protostomia 5%
Taphrinomycotina 3%
saccharomyceta 3%
Tracheophyta 13%
Dictyosteliales 3%
Pyrococcus 3%
Eggerthellaceae 13%
Coriobacteriaceae 3%
Pseudonocardiaceae 3%
Actinomycetaceae 3%
Streptococcaceae 3%
Bacillaceae 3%
Merismopediaceae 3%
Chloroflexi bacterium RBG_13_53_26 3%
Cereibacter 3%
Caenibius 3%

IR2

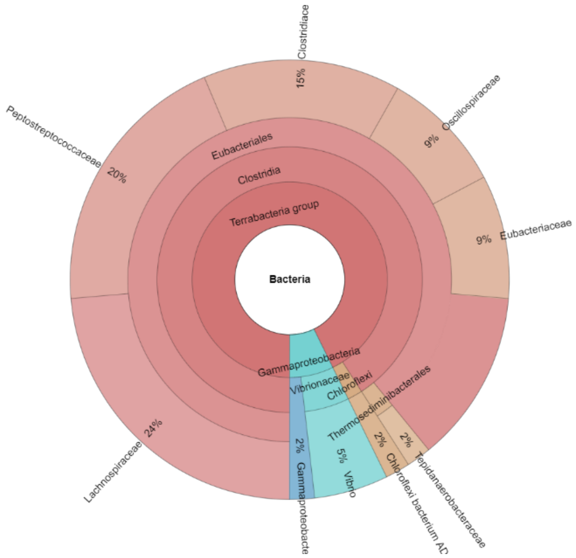

Bacteria
Terrabacteria group
Clostridia
Eubacteriales
Gammaproteobacteria
Vibrionaceae
Chloroflexi
Thermosediminibacterales
15% Clostridiace
9% Oscillospiraceae
9% Eubacteriaceae
20% Peptostreptococcaceae
24% Lachnospiraceae
2% Gammaproteobacte
5% Vibrio
2% Chloroflexi bacterium AD
2% Tepidanaerobacteraceae

NCR1

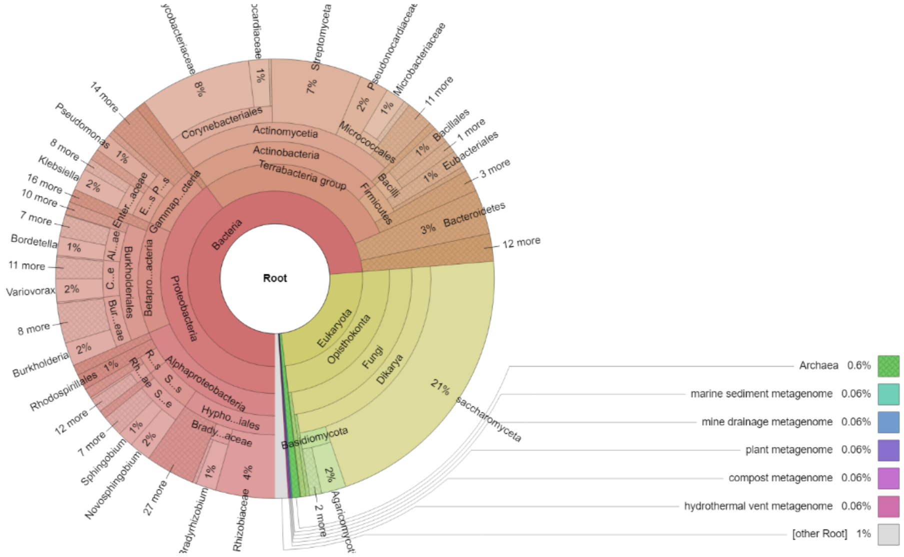
Root
Bacteria
Proteobacteria
Terrabacteria group
Actinobacteria
Actinomycetia
Corynebacteriales
8%
1%
Streptomyceta
7%
Pseudonocardiaceae
Microbacteriaceae
2%
1%
Micrococcales
11 more
Bacillales
1 more
Eubacteriales
Bacilli
Firmicutes
3 more
Bacteroidetes
3%
12 more
14 more
Pseudomonas
8 more
Klebsiella
16 more
10 more
7 more
Bordetella
11 more
Variovorax
8 more
Burkholderia
Rhodospirillales
12 more
7 more
Sphingobium
Novosphingobium
27 more
Bradyrhizobium
Rhizobiaceae
4%
Gammap...cteria
Betapro...acteria
Burkholderiales
Alphaproteobacteria
Hypho...iales
Brady...aceae
Eukaryota
Opisthokonta
Fungi
Dikarya
21%
Saccharomyceta
Basidiomycota
2 more
Archaea 0.6%
marine sediment metagenome 0.06%
mine drainage metagenome 0.06%
plant metagenome 0.06%
compost metagenome 0.06%
hydrothermal vent metagenome 0.06%
[other Root] 1%

OR1

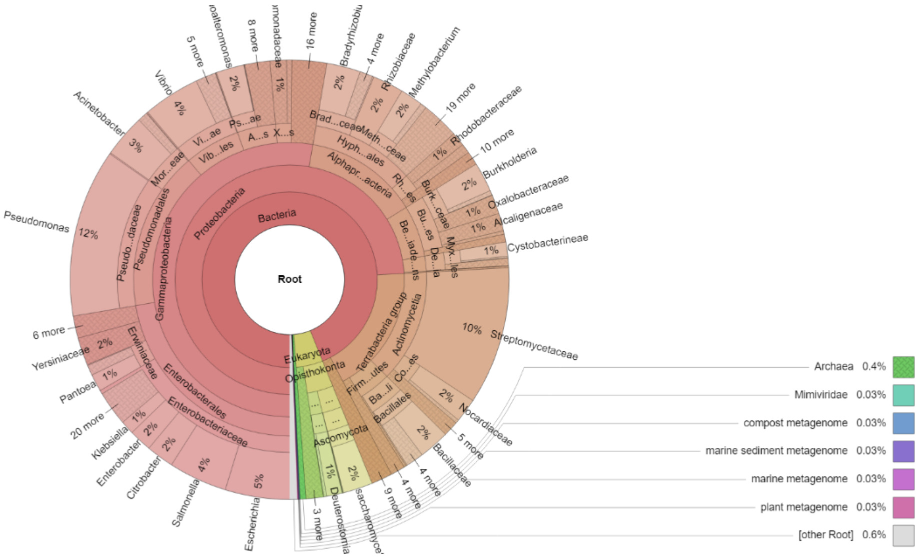

Root
Bacteria
Proteobacteria
Gammaproteobacteria
Pseudomonadales
Pseudomonas
12%
Acinetobacter
3%
Vibrio
4%
Enterobacterales
Enterobacteriaceae
Escherichia
5%
Salmonella
4%
Citrobacter
Enterobacter
Klebsiella
Pantoea
Yersiniaceae
Erwiniaceae
Alphaproteobacteria
Bradyrhizobium
Rhizobiaceae
Methylobacterium
Rhodobacteraceae
Burkholderia
Oxalobacteraceae
Alcaligenaceae
Cystobacterineae
Terrabacteria group
Actinomycetia
Streptomycetaceae
10%
Nocardiaceae
Bacillales
Bacillaceae
Eukaryota
Opisthokonta
Ascomycota
Deuterostomia
Archaea 0.4%
Mimiviridae 0.03%
compost metagenome 0.03%
marine sediment metagenome 0.03%
marine metagenome 0.03%
plant metagenome 0.03%
[other Root] 0.6%

OR2

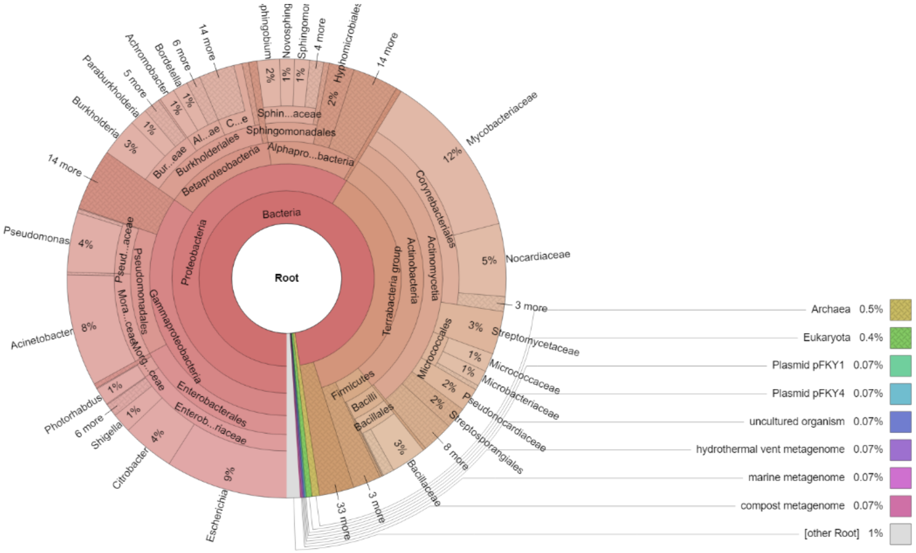
Root
Bacteria
Proteobacteria
Terrabacteria group
Actinobacteria
Actinomycetia
Corynebacteriales
Mycobacteriaceae
12%
Nocardiaceae
5%
3 more
Streptomycetaceae
3%
Micrococcales
Micrococcaceae
1%
Microbacteriaceae
1%
Pseudonocardiaceae
2%
Streptosporangiales
2%
8 more
Firmicutes
Bacilli
Bacillales
Bacillaceae
3%
3 more
33 more
Gammaproteobacteria
Enterobacterales
Enterob...riaceae
Escherichia
9%
Citrobacter
4%
Shigella
1%
6 more
Photorhabdus
1%
Pseudomonadales
Mora...ceae
Acinetobacter
8%
Pseud...aceae
Pseudomonas
4%
14 more
Betaproteobacteria
Burkholderiales
Bur...eae
Burkholderia
3%
Paraburkholderia
1%
5 more
Al...ae
Achromobacter
Bordetella
1%
6 more
1%
C...e
14 more
Alphapro...bacteria
Sphingomonadales
Sphin...aceae
2%
1%
Novosphing
1%
Sphingomon
4 more
Hyphomicrobiales
2%
14 more
Archaea 0.5%
Eukaryota 0.4%
Plasmid pFKY1 0.07%
Plasmid pFKY4 0.07%
uncultured organism 0.07%
hydrothermal vent metagenome 0.07%
marine metagenome 0.07%
compost metagenome 0.07%
[other Root] 1%

OR3

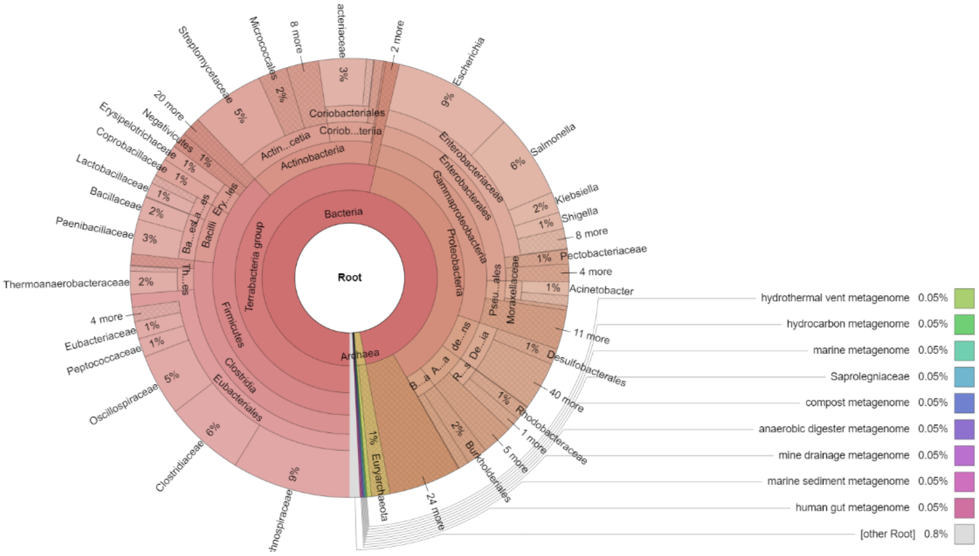

Root
Bacteria
Archaea
Terrabacteria group
Proteobacteria
Actinobacteria
Firmicutes
Clostridia
Eubacteriales
Bacilli
Gammaproteobacteria
Enterobacterales
Enterobacteriaceae
Escherichia 9%
Salmonella 6%
Klebsiella 2%
Shigella 1%
8 more
Pectobacteriaceae 1%
4 more
Acinetobacter 1%
Moraxellaceae
11 more
Desulfobacterales 1%
40 more
Rhodobacteraceae 1%
1 more
5 more
Burkholderiales 2%
24 more
Euryarchaeota 1%
Coriobacteriales
Coriob...teriia
Actin...cetia
Streptomycetaceae 5%
Micrococcales 2%
8 more
acteriaceae 3%
2 more
20 more
Negativicutes 1%
Erysipelotrichaceae 1%
Coprobacillaceae 1%
Lactobacillaceae 1%
Bacillaceae 2%
Paenibacillaceae 3%
Thermoanaerobacteraceae 2%
4 more
Eubacteriaceae 1%
Peptococcaceae 1%
Oscillospiraceae 5%
Clostridiaceae 6%
chnospiraceae 9%
hydrothermal vent metagenome 0.05%
hydrocarbon metagenome 0.05%
marine metagenome 0.05%
Saprolegniaceae 0.05%
compost metagenome 0.05%
anaerobic digester metagenome 0.05%
mine drainage metagenome 0.05%
marine sediment metagenome 0.05%
human gut metagenome 0.05%
[other Root] 0.8%

OR4

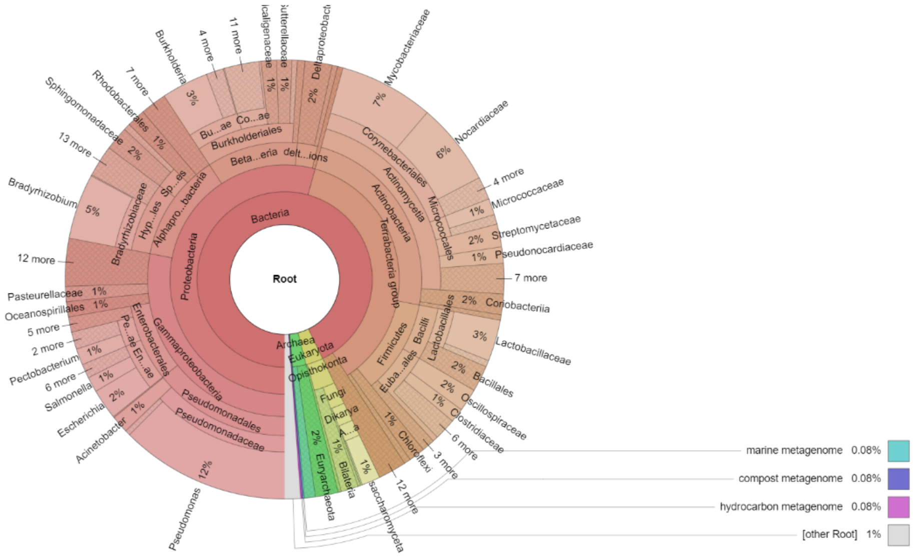
Root
Bacteria
Proteobacteria
Terrabacteria group
Actinobacteria
Actinomycetia
Corynebacteriales
Mycobacteriaceae
Nocardiaceae
Micrococcales
Micrococcaceae
Streptomycetaceae
Pseudonocardiaceae
Coriobacteriia
Firmicutes
Bacilli
Lactobacillales
Lactobacillaceae
Bacillales
Oscillospiraceae
Clostridiaceae
Chloroflexi
Gammaproteobacteria
Pseudomonadales
Pseudomonadaceae
Pseudomonas
Enterobacterales
Acinetobacter
Escherichia
Salmonella
Pectobacterium
Oceanospirillales
Pasteurellaceae
Bradyrhizobium
Bradyrhizobiaceae
Sphingomonadaceae
Rhodobacterales
Burkholderia
Burkholderiales
Deltaproteobacteria
Archaea
Euryarchaeota
Eukaryota
Opisthokonta
Fungi
Dikarya
Bilateria
saccharomyceta
marine metagenome 0.08%
compost metagenome 0.08%
hydrocarbon metagenome 0.08%
[other Root] 1%

OR5

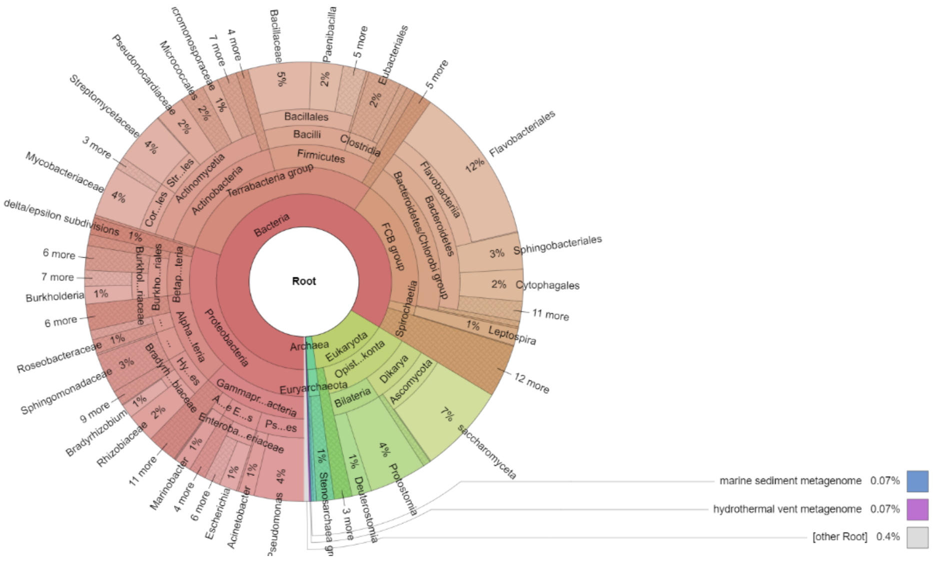

Root
Bacteria
Terrabacteria group
Firmicutes
Bacilli
Bacillales
Bacillaceae
5%
Paenibacilla
2%
5 more
Clostridia
Eubacteriales
2%
5 more
Actinobacteria
Actinomycetia
Str...les
Cor...les
Streptomycetaceae
4%
3 more
Mycobacteriaceae
4%
Pseudonocardiaceae
2%
Micrococcales
2%
7 more
1%
4 more
FCB group
Bacteroidetes/Chlorobi group
Bacteroidetes
Flavobacteriia
Flavobacteriales
12%
Sphingobacteriales
3%
Cytophagales
2%
11 more
Spirochaetia
Leptospira
1%
12 more
Proteobacteria
delta/epsilon subdivisions
1%
Betap...teria
Burkho...riales
Burkhol...riaceae
6 more
7 more
Burkholderia
1%
6 more
Alpha...teria
Hy...es
Roseobacteraceae
1%
Sphingomonadaceae
3%
9 more
Bradyrhizobium
1%
Rhizobiaceae
2%
11 more
Gammapr...acteria
Enteroba...eriaceae
Ps...es
Marinobacter
1%
4 more
6 more
Escherichia
1%
Acinetobacter
1%
Pseudomonas
4%
Archaea
Euryarchaeota
1%
Eukaryota
Opist...konta
Dikarya
Ascomycota
saccharomyceta
7%
Bilateria
Protostomia
4%
Deuterostomia
1%
3 more
marine sediment metagenome 0.07%
hydrothermal vent metagenome 0.07%
[other Root] 0.4%

OR6

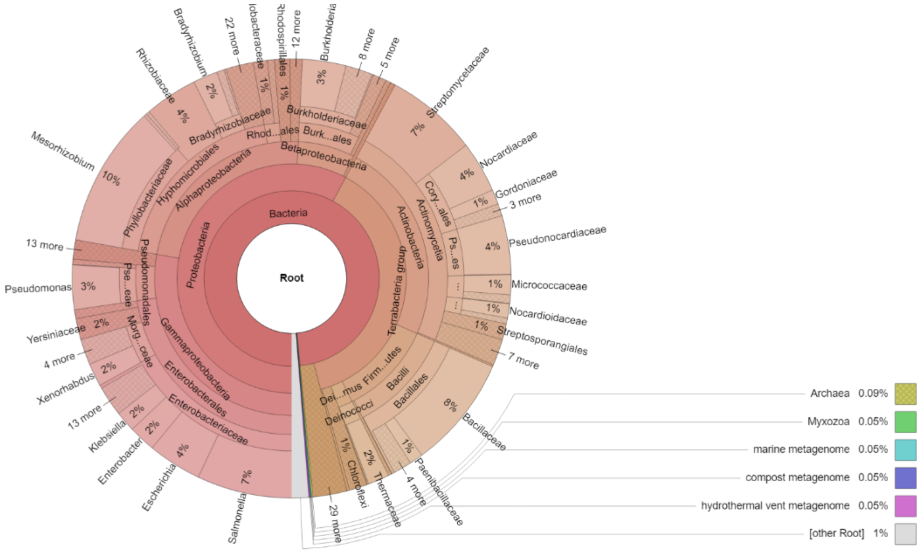

Root
Bacteria
Proteobacteria
Terrabacteria group
Actinobacteria
Alphaproteobacteria
Betaproteobacteria
Gammaproteobacteria
Mesorhizobium 10%
Salmonella 7%
Streptomycetaceae 7%
Bacillaceae 8%
Archaea 0.09%
Myxozoa 0.05%
marine metagenome 0.05%
compost metagenome 0.05%
hydrothermal vent metagenome 0.05%
[other Root] 1%

OR7

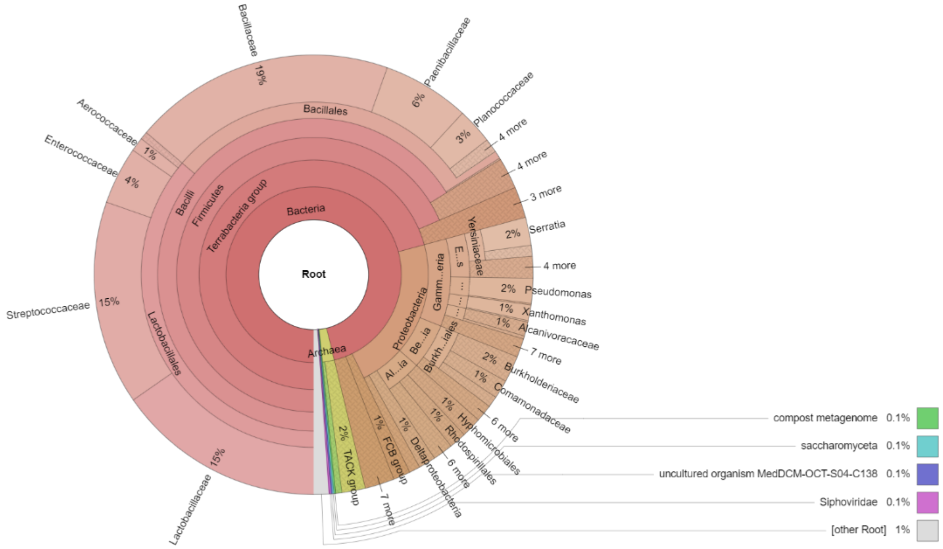
Root
Bacteria
Archaea
Terrabacteria group
Firmicutes
Bacilli
Bacillales
Lactobacillales
Bacillaceae 19%
Paenibacillaceae 6%
Planococcaceae 3%
4 more
Aerococcaceae 1%
Enterococcaceae 4%
Streptococcaceae 15%
Lactobacillaceae 15%
4 more
3 more
Proteobacteria
Gamm...eria
E...s
Yersiniaceae
Serratia 2%
4 more
Pseudomonas 2%
Xanthomonas 1%
Alcanivoracaceae 1%
7 more
Be...ia
Burkh...iales
Burkholderiaceae 2%
Comamonadaceae 1%
6 more
Al...ia
Hyphomicrobiales 1%
Rhodospirillales 1%
6 more
Deltaproteobacteria 1%
FCB group 1%
7 more
TACK group 2%
compost metagenome 0.1%
saccharomyceta 0.1%
uncultured organism MedDCM-OCT-S04-C138 0.1%
Siphoviridae 0.1%
[other Root] 1%

OR8

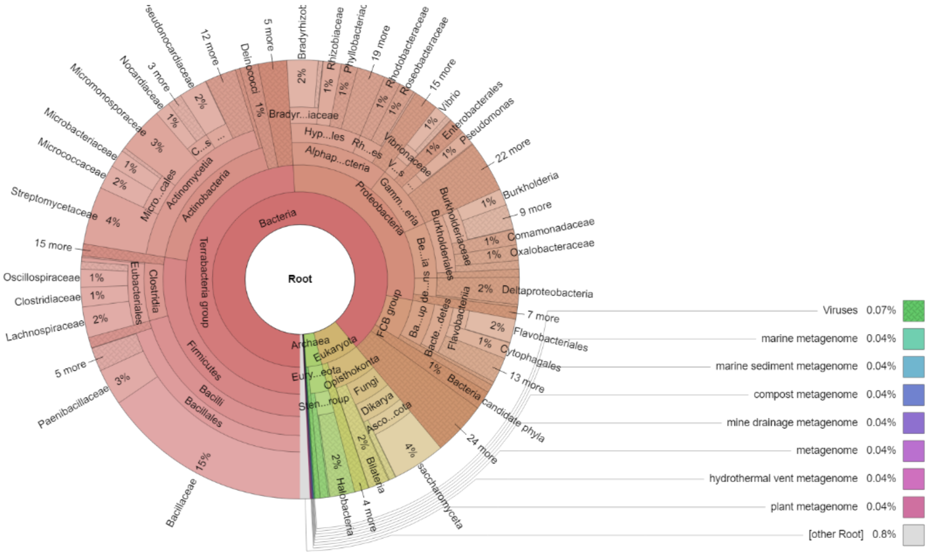
Root
Bacteria
Terrabacteria group
Actinobacteria
Actinomycetia
Firmicutes
Bacilli
Bacillales
Bacillaceae
15%
Paenibacillaceae
3%
5 more
Clostridia
Eubacteriales
Oscillospiraceae
Clostridiaceae
Lachnospiraceae
15 more
Streptomycetaceae
4%
Micrococcaceae
Microbacteriaceae
Micromonosporaceae
Nocardiaceae
3 more
12 more
Deinococci
5 more
Bradyrhizobiaceae
Rhizobiaceae
Phyllobacteriaceae
19 more
Rhodobacteraceae
Roseobacteraceae
15 more
Vibrio
Enterobacterales
Pseudomonas
22 more
Burkholderia
9 more
Comamonadaceae
Oxalobacteraceae
Deltaproteobacteria
7 more
Flavobacteriales
Cytophagales
13 more
Bacteria candidate phyla
24 more
Proteobacteria
Alphap...cteria
Gamm...eria
Burkholderiales
Burkholderiaceae
FCB group
Flavobacteriia
Archaea
Eukaryota
Opisthokonta
Fungi
Dikarya
Asco...cota
Saccharomyceta
Bilateria
4 more
Halobacteria
Viruses 0.07%
marine metagenome 0.04%
marine sediment metagenome 0.04%
compost metagenome 0.04%
mine drainage metagenome 0.04%
metagenome 0.04%
hydrothermal vent metagenome 0.04%
plant metagenome 0.04%
[other Root] 0.8%

OR9

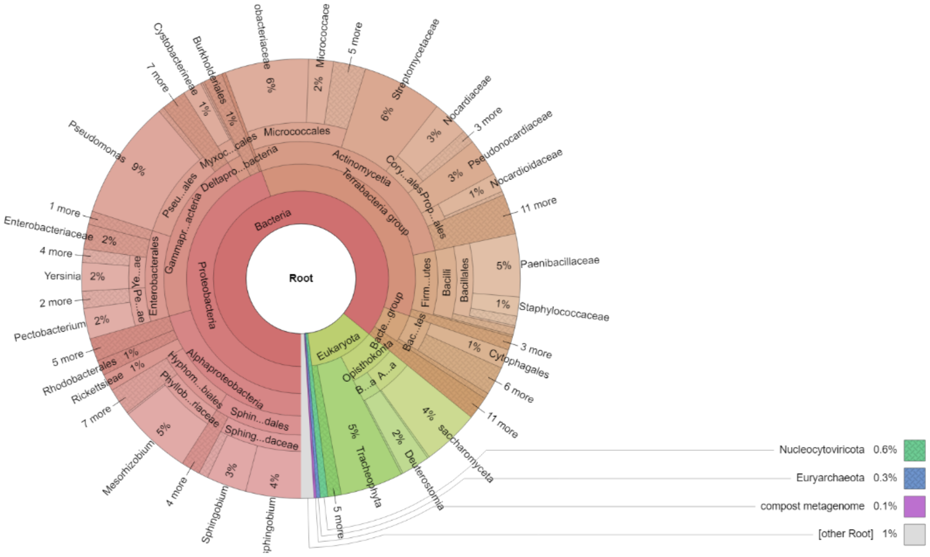
Root
Bacteria
Proteobacteria
Terrabacteria group
Actinomycetia
Micrococcales
Micrococcaceae
2%
5 more
Streptomycetaceae
6%
Nocardiaceae
3%
3 more
Pseudonocardiaceae
3%
Nocardioidaceae
1%
11 more
Cory...ales
Prop...ales
Firm...utes
Bacilli
Bacillales
Paenibacillaceae
5%
Staphylococcaceae
1%
3 more
Cytophagales
6 more
11 more
Bacte...group
Bac...tes
Eukaryota
Opisthokonta
B...a
A...a
4%
saccharomyceta
5%
Tracheophyta
2%
Deuterostomia
5 more
Nucleocytoviricota 0.6%
Euryarchaeota 0.3%
compost metagenome 0.1%
[other Root] 1%
Alphaproteobacteria
Sphin...dales
Sphing...daceae
4%
3%
Sphingobium
4 more
Hyphom...biales
Phyllob...riaceae
5%
Mesorhizobium
7 more
Rickettsieae
1%
Rhodobacterales
1%
5 more
Pectobacterium
2%
2 more
Yersinia
2%
4 more
Enterobacteriaceae
2%
1 more
Enterobacterales
Ye...ae
Pe...ae
Gammapr...acteria
Pseu...ales
Pseudomonas
9%
7 more
Cystobacterineae
1%
Burkholderiales
1%
Myxoc...cales
Deltapro...bacteria
6%

UC1

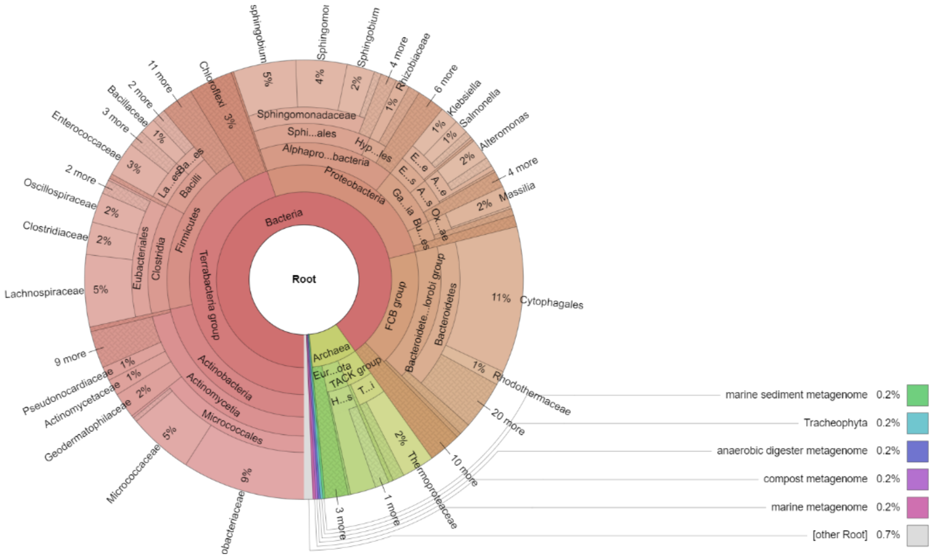

Root
Bacteria
Terrabacteria group
Proteobacteria
FCB group
Archaea
Firmicutes
Actinobacteria
Actinomycetia
Micrococcales
Alphapro...bacteria
Sphi...ales
Sphingomonadaceae
Bacteroidete...lorobi group
Bacteroidetes
Cytophagales
Rhodothermaceae
Thermoproteaceae
TACK group
Eur...ota
Clostridia
Eubacteriales
Bacilli
Lachnospiraceae
Clostridiaceae
Oscillospiraceae
Enterococcaceae
Bacillaceae
Chloroflexi
Micrococcaceae
Geodermatophilaceae
Actinomycetaceae
Pseudonocardiaceae
Sphingomonas
Sphingobium
Rhizobiaceae
Klebsiella
Salmonella
Alteromonas
Massilia
11 more
2 more
3 more
9 more
4 more
6 more
20 more
10 more
1 more
3 more
11%
9%
5%
4%
3%
2%
1%
marine sediment metagenome 0.2%
Tracheophyta 0.2%
anaerobic digester metagenome 0.2%
compost metagenome 0.2%
marine metagenome 0.2%
[other Root] 0.7%

UC2

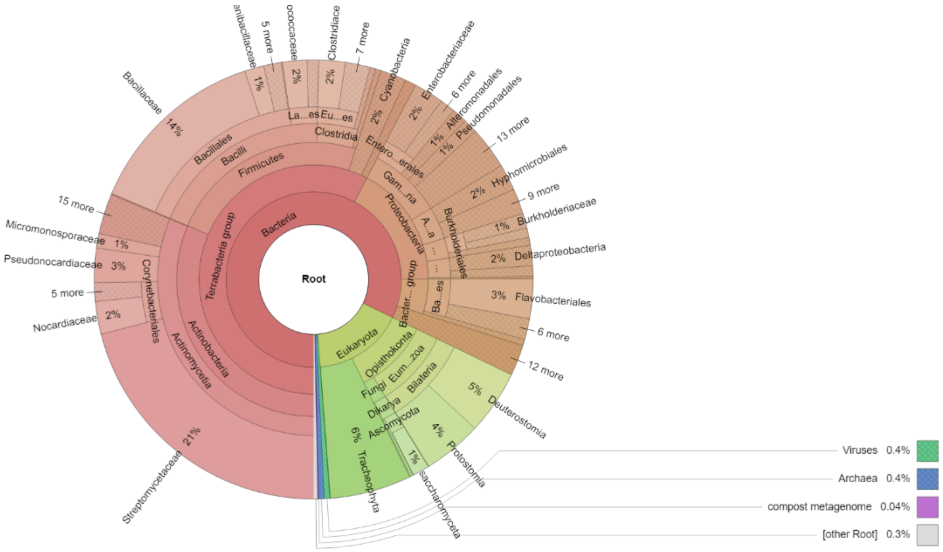
Root
Bacteria
Terrabacteria group
Firmicutes
Bacilli
Bacillales
Bacillaceae
14%
Clostridia
Actinobacteria
Actinomycetia
Corynebacteriales
Streptomycetaceae
21%
Micromonosporaceae
Pseudonocardiaceae
Nocardiaceae
15 more
5 more
Proteobacteria
Cyanobacteria
Enterobacteriaceae
Alteromonadales
Pseudomonadales
Hyphomicrobiales
Burkholderiales
Burkholderiaceae
Deltaproteobacteria
Flavobacteriales
13 more
9 more
6 more
12 more
7 more
Eukaryota
Opisthokonta
Fungi
Dikarya
Ascomycota
Bilateria
Deuterostomia
Protostomia
Tracheophyta
saccharomyceta
Viruses 0.4%
Archaea 0.4%
compost metagenome 0.04%
[other Root] 0.3%

Data S4

Boxplots of the percentages of PUP homologs in genomes of each major phylum

Firmicutes A

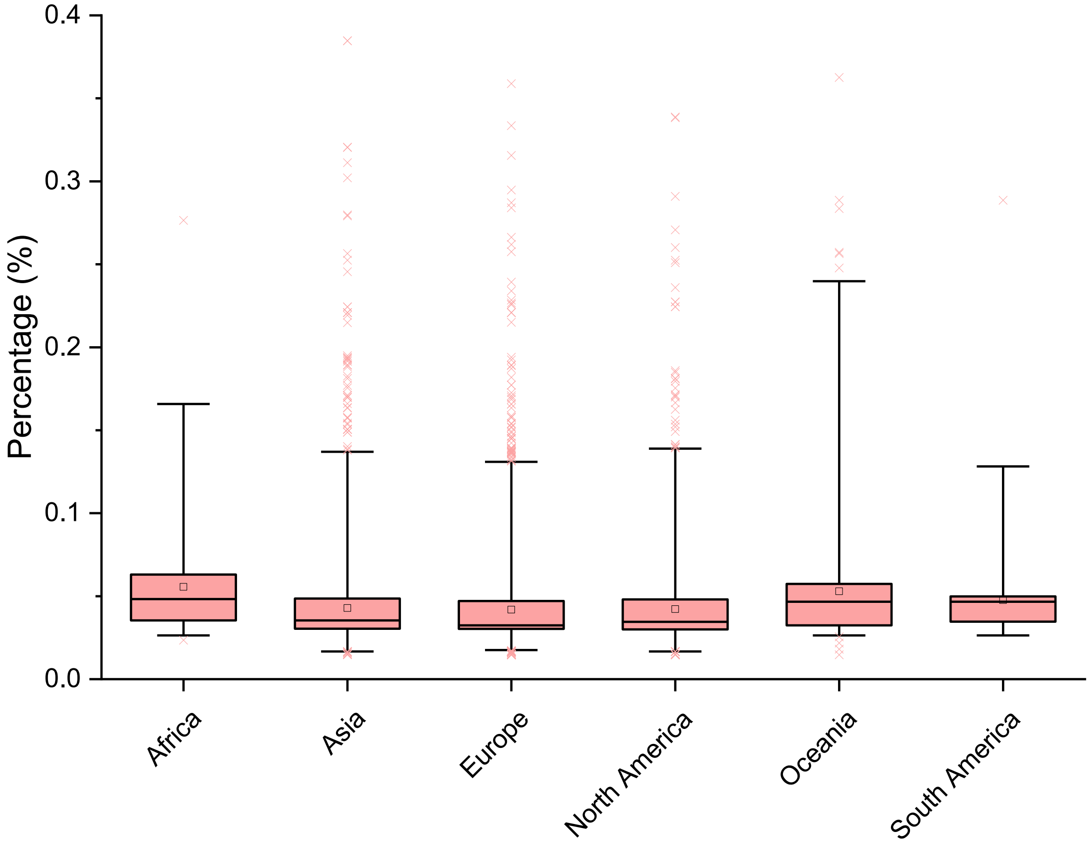

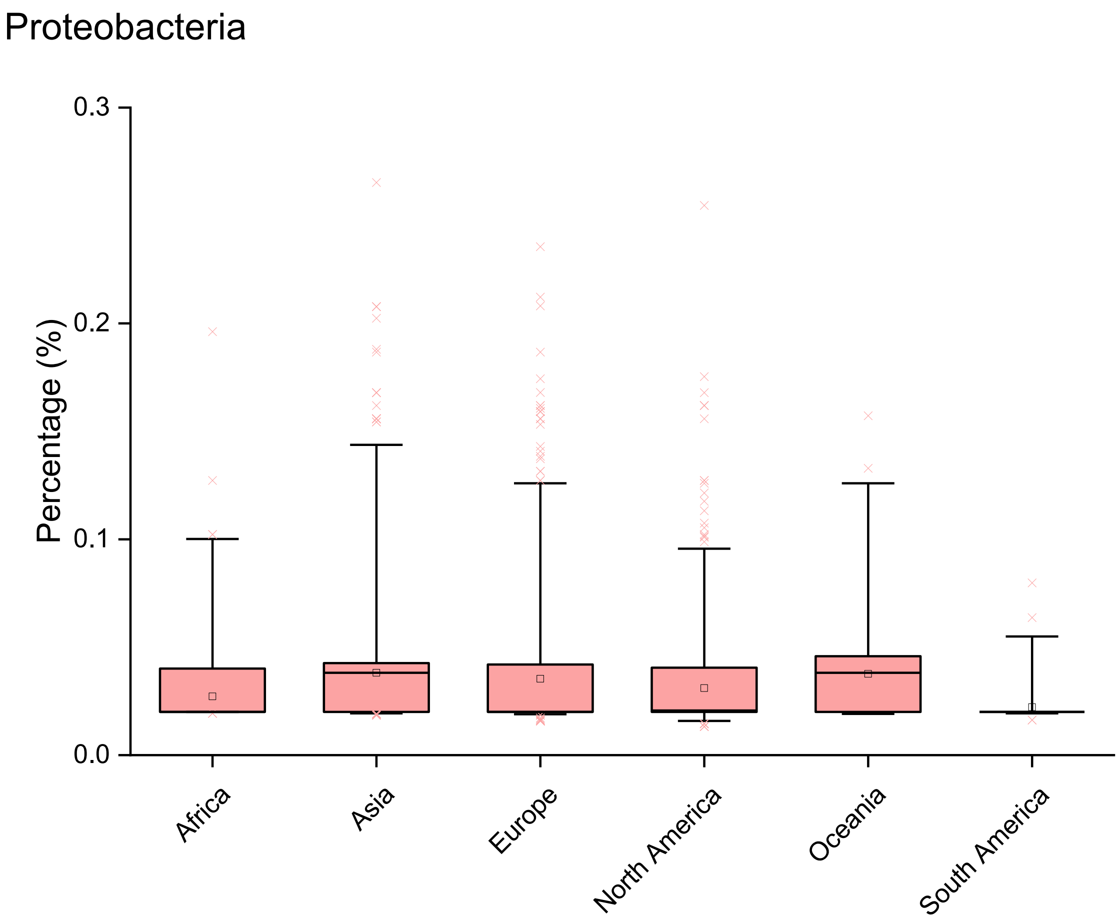
Proteobacteria
Percentage (%)
0.3
0.2
0.1
0.0
Africa
Asia
Europe
North America
Oceania
South America

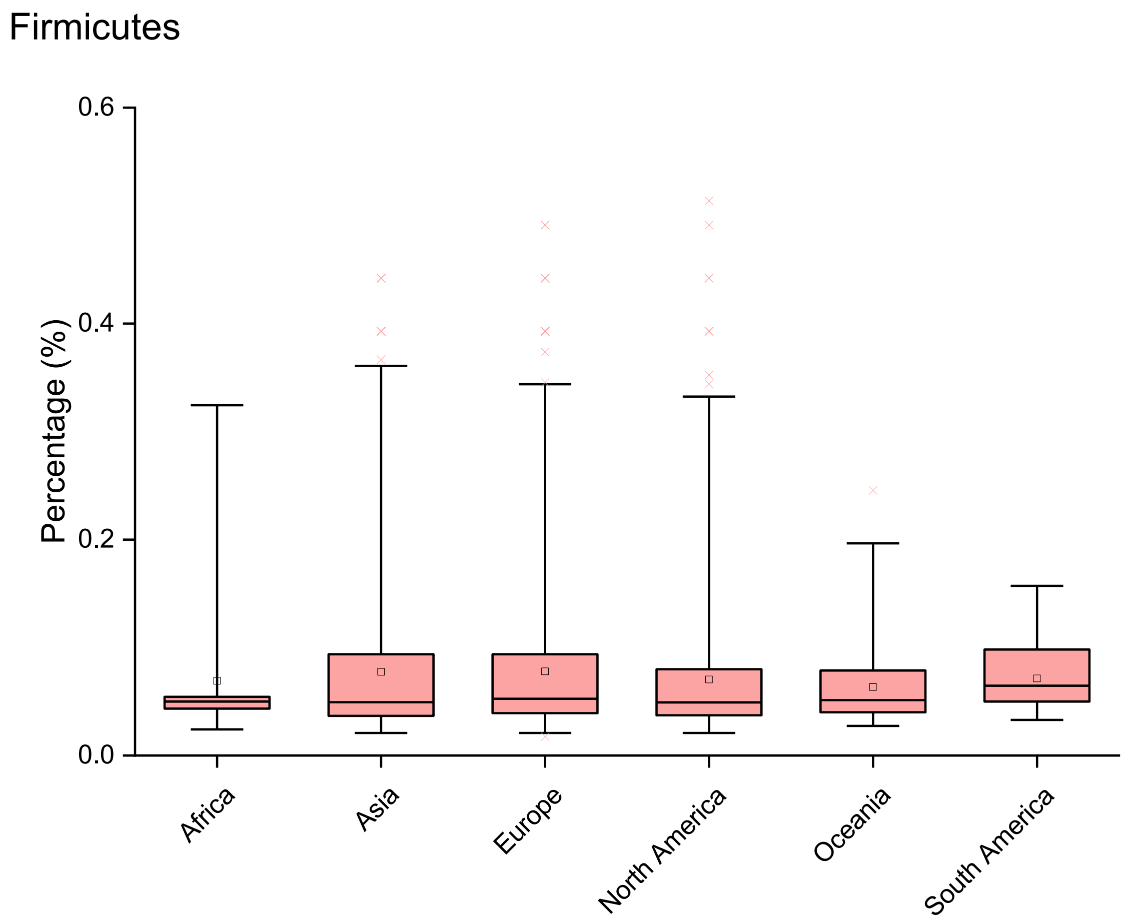
Firmicutes
Percentage (%)
0.6
0.4
0.2
0.0
Africa
Asia
Europe
North America
Oceania
South America

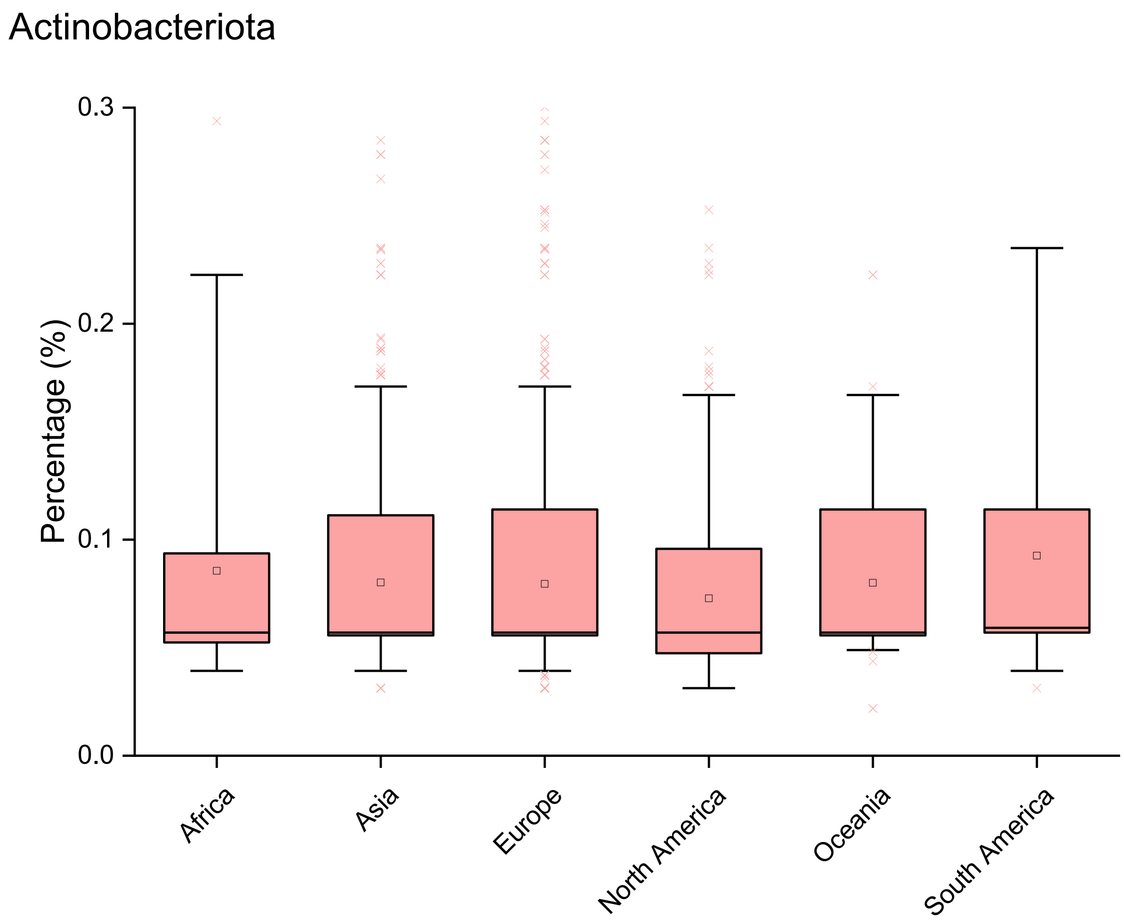
Actinobacteriota
Percentage (%)
0.3
0.2
0.1
0.0
Africa
Asia
Europe
North America
Oceania
South America

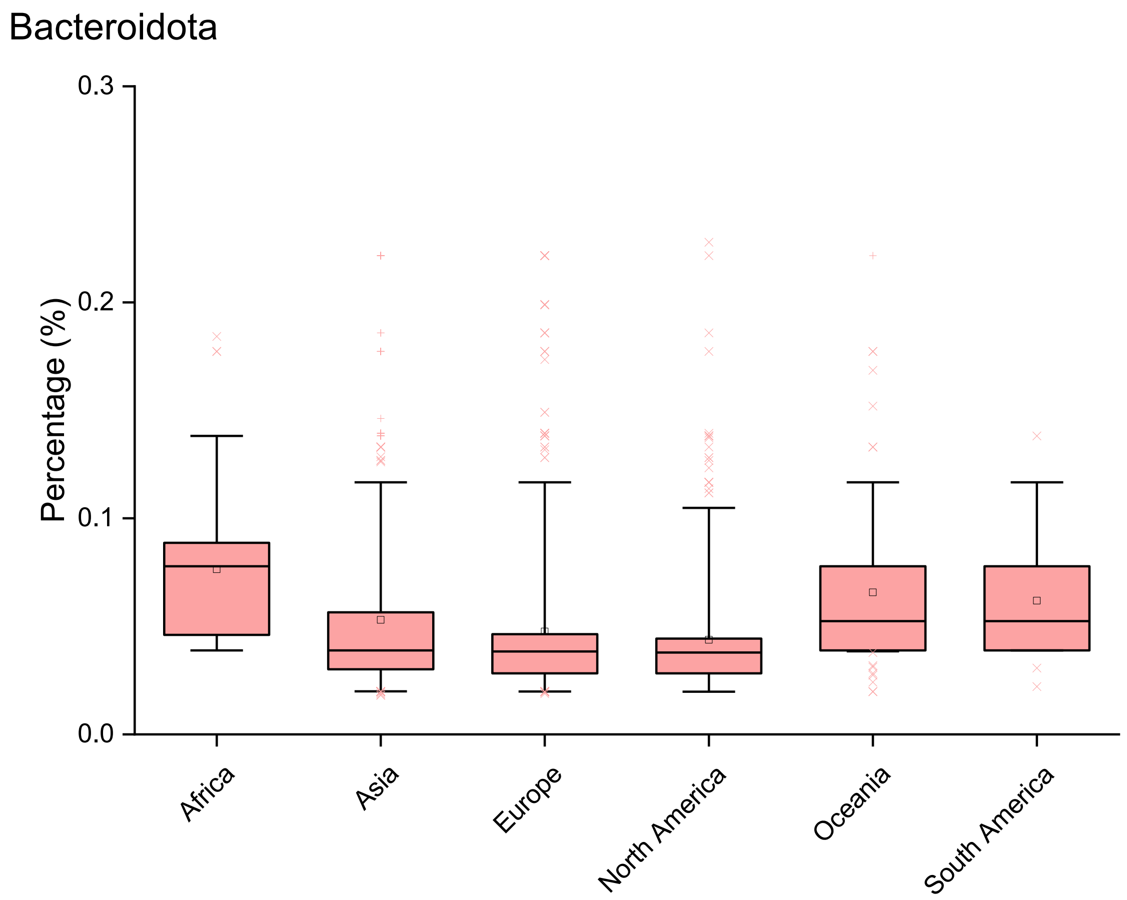

Bacteroidota
Percentage (%)
0.3
0.2
0.1
0.0
Africa
Asia
Europe
North America
Oceania
South America

Firmicutes C

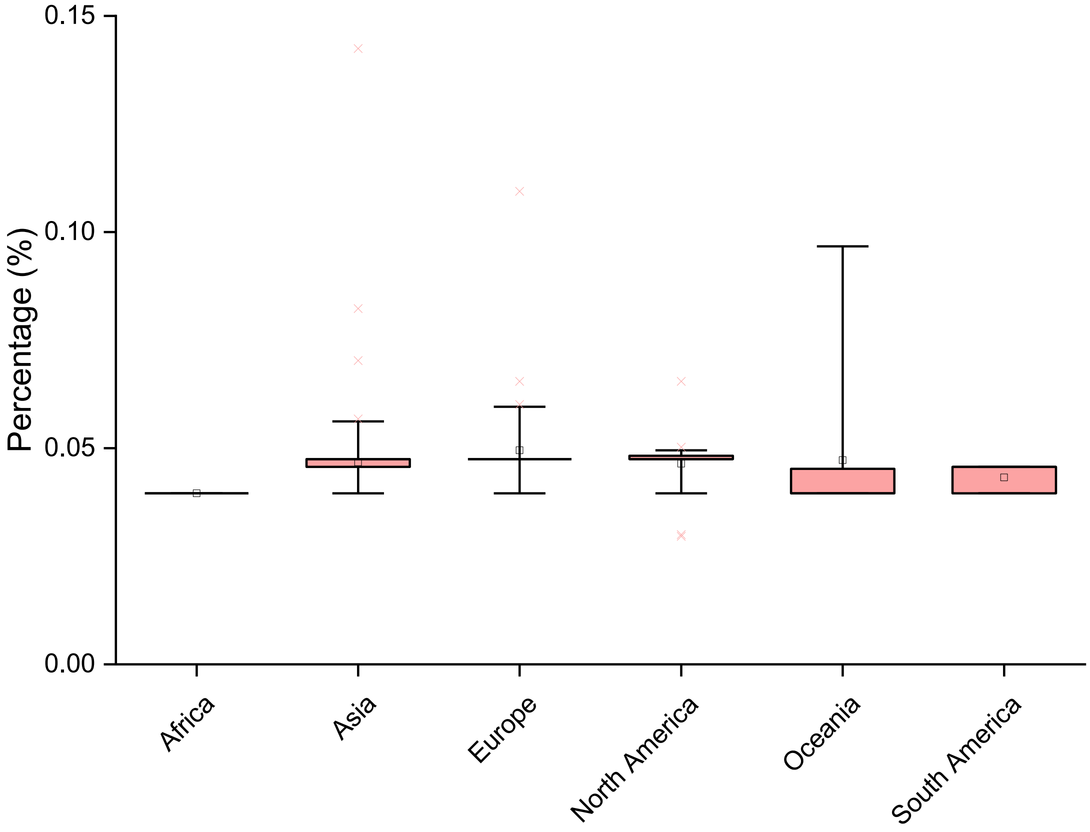

Verrucomicrobiota

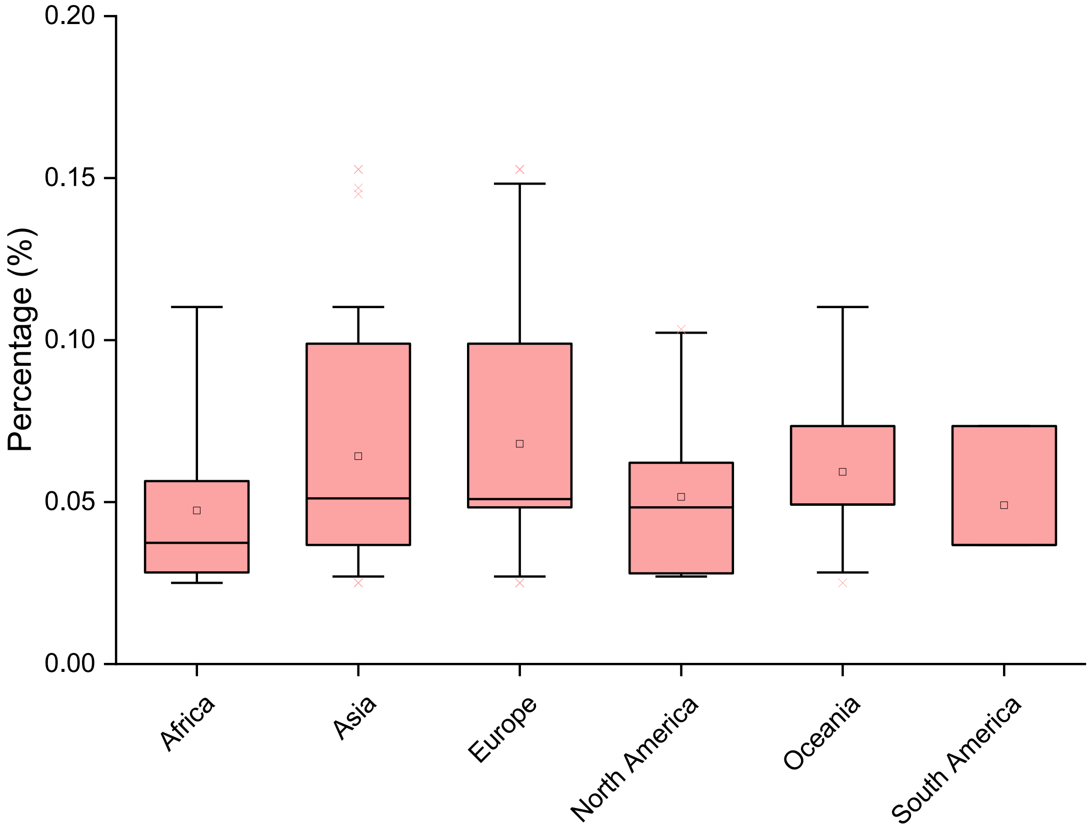

Data S5

Boxplots of the percentages of PGC genes in genomes of each major phylum

Firmicutes

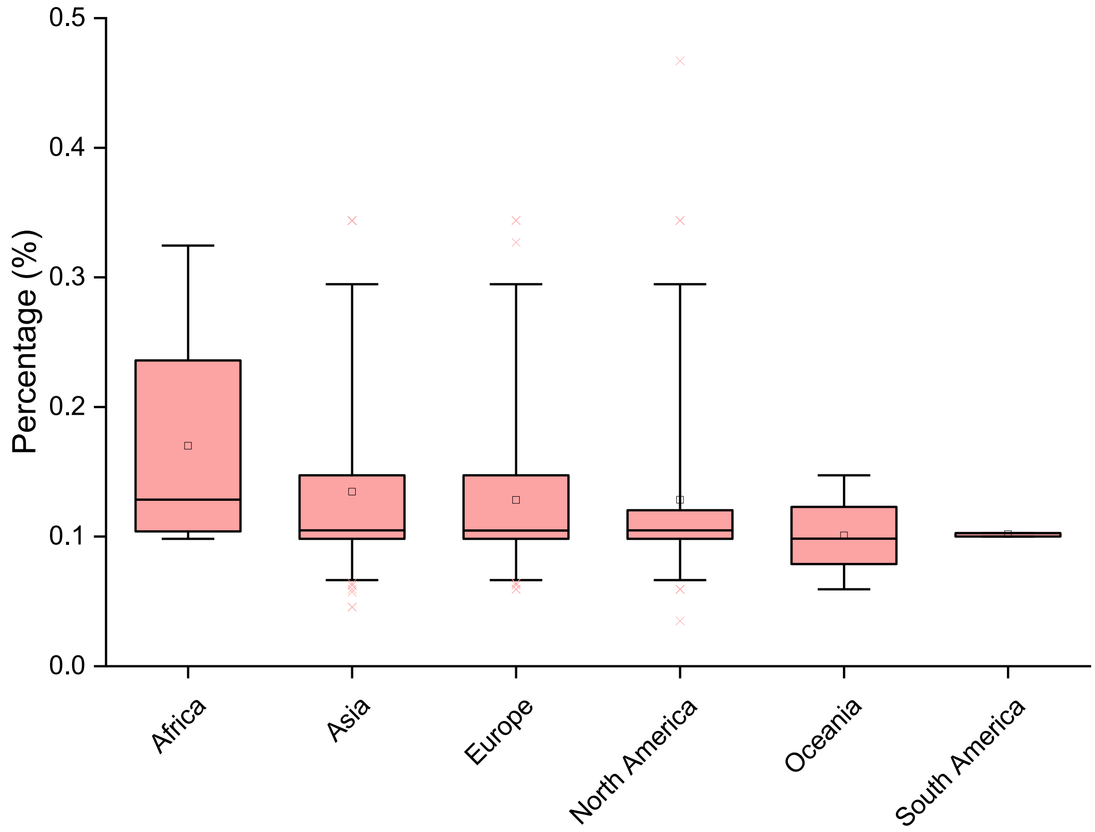

Firmicutes A

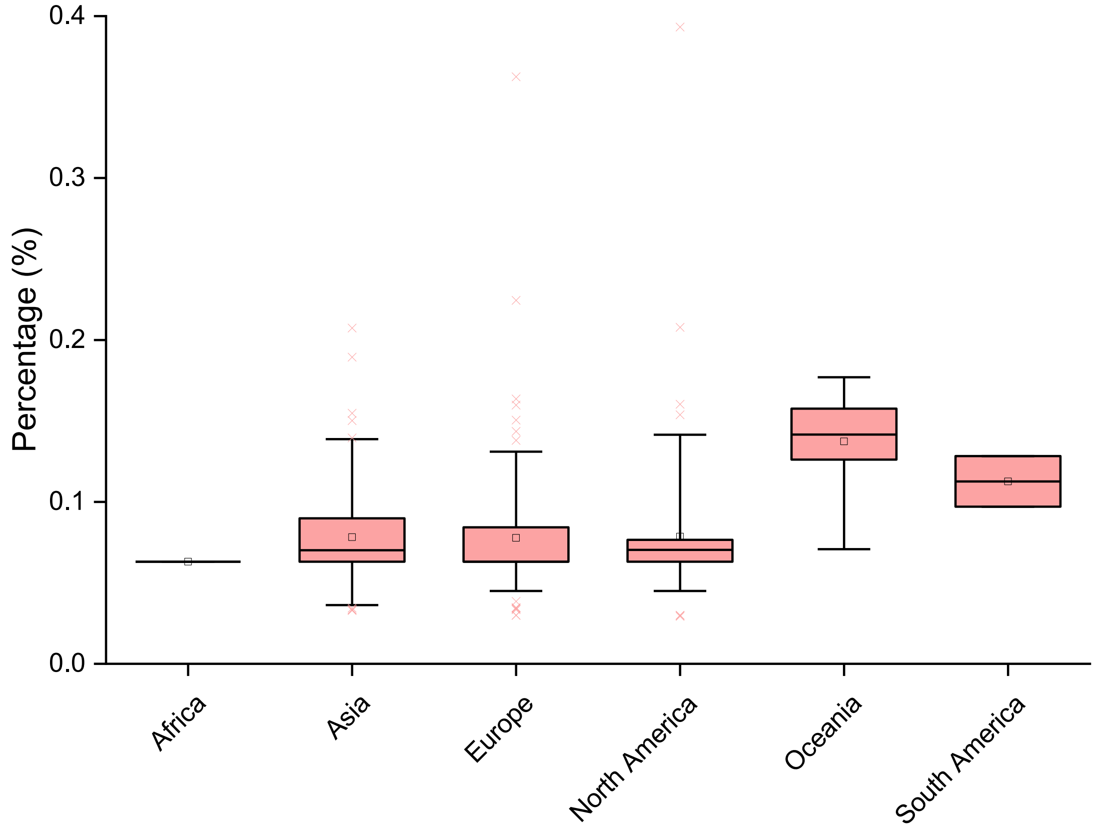

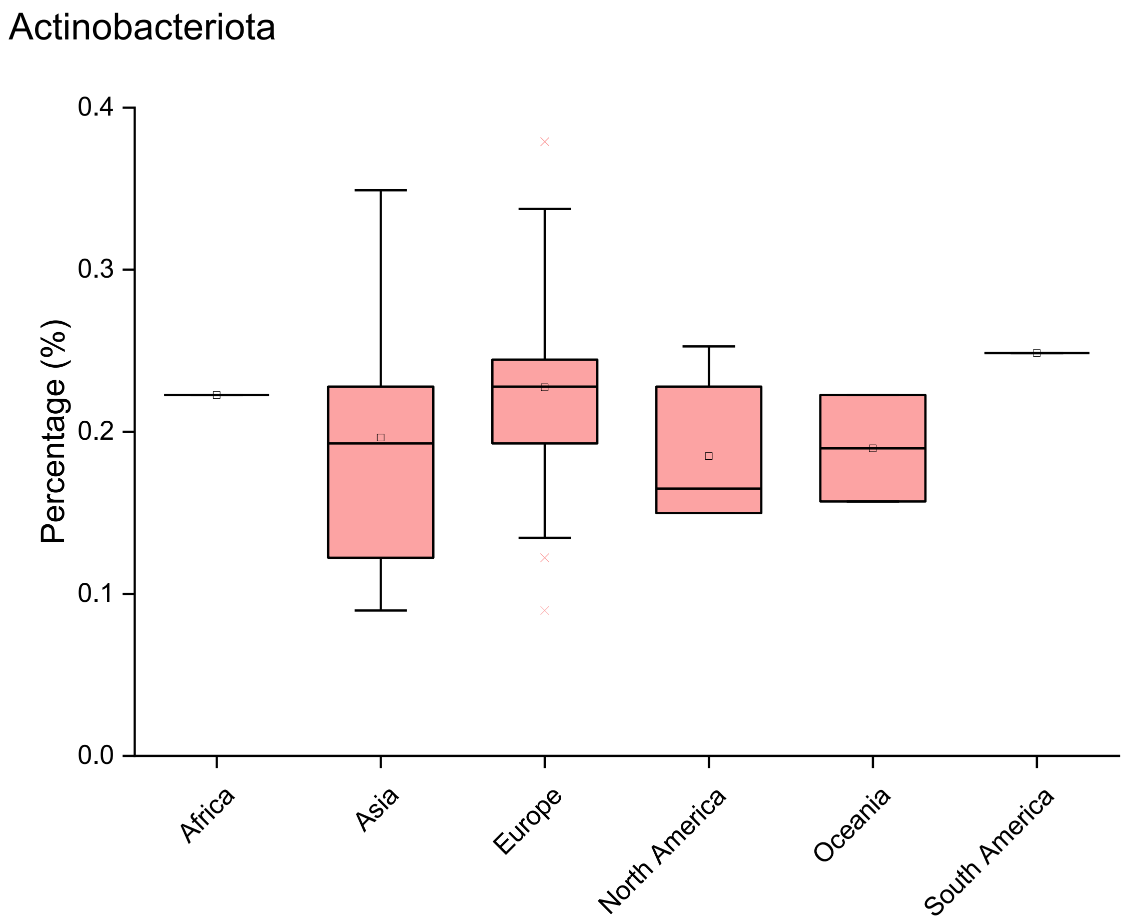

Actinobacteriota
Percentage (%)
0.0
0.1
0.2
0.3
0.4
Africa
Asia
Europe
North America
Oceania
South America

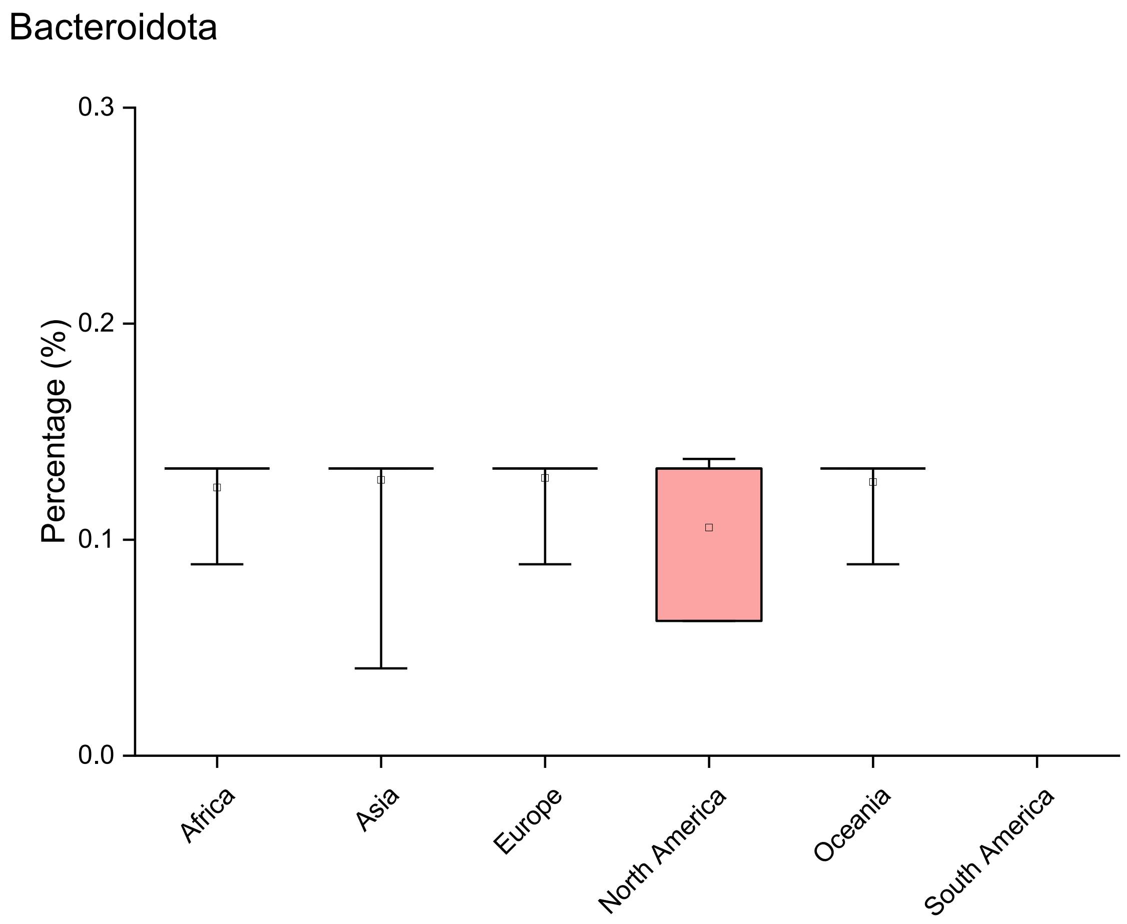

Bacteroidota
Percentage (%)
0.3
0.2
0.1
0.0
Africa
Asia
Europe
North America
Oceania
South America

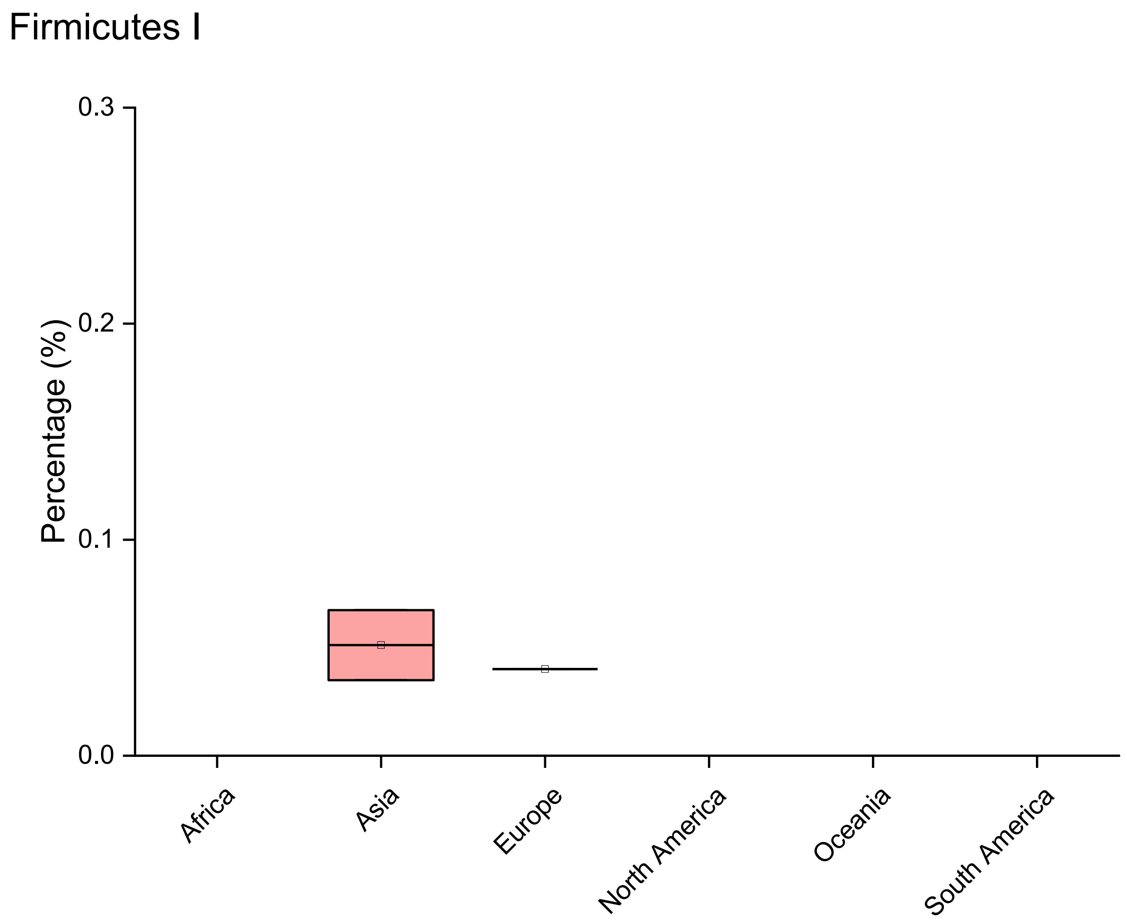
Firmicutes I
Percentage (%)
0.3
0.2
0.1
0.0
Africa
Asia
Europe
North America
Oceania
South America
